# Mutant prion protein enhances NMDA receptor activity, activates PKC, and triggers rapid excitotoxicity in mice

**DOI:** 10.1172/JCI186432

**Published:** 2025-04-04

**Authors:** Joie Lin, Julia A. Callender, Joshua E. Mayfield, Daniel B. McClatchy, Daniel Ojeda-Juárez, Mahsa Pourhamzeh, Katrin Soldau, Timothy D. Kurt, Garrett A. Danque, Helen Khuu, Josephina E. Ronson, Donald P. Pizzo, Yixing Du, Maxwell A. Gruber, Alejandro M. Sevillano, Jin Wang, Christina D. Orrú, Joy Chen, Gail Funk, Patricia Aguilar-Calvo, Brent D. Aulston, Subhojit Roy, Jong M. Rho, Jack D. Bui, Alexandra C. Newton, Stuart A. Lipton, Byron Caughey, Gentry N. Patrick, Kim Doré, John R. Yates, Christina J. Sigurdson

**Affiliations:** 1 Department of Pathology, UCSD, La Jolla, California, USA.; 2 Department of Molecular Medicine, The Scripps Research Institute, La Jolla, California, USA.; 3 Department of Neurosciences, UCSD, School of Medicine, La Jolla, California, USA.; 4 Laboratory of Persistent Viral Diseases, Rocky Mountain Laboratories, National Institute of Allergy and Infectious Diseases (NIAID), NIH, Hamilton, Montana, USA.; 5 Department of Pharmacology, UCSD, La Jolla, California, USA.; 6 Neurodegeneration New Medicines Center and Department of Molecular & Cellular Biology, The Scripps Research Institute, La Jolla, California, USA.; 7 Department of Biology, and; 8 Department of Medicine, UCSD, La Jolla, California, USA.; 9 Department of Pathology, Microbiology, and Immunology, UCD, Davis, California, USA.

**Keywords:** Aging, Neuroscience, Neurodegeneration, Prions, Synapses

## Abstract

Neuronal hyperexcitability precedes synapse loss in certain neurodegenerative diseases, yet the synaptic membrane interactions and downstream signaling events remain unclear. The disordered amino terminus of the prion protein (PrP^C^) has been implicated in aberrant signaling in prion and Alzheimer’s disease. To disrupt neuronal interactions and signaling linked to the amino terminus, we CRISPR-engineered a knockin mouse expressing mutant PrP^C^ (G92N), generating an *N*-linked glycosylation site between 2 functional motifs. Mice developed seizures and necrosis of hippocampal pyramidal neurons, similar to prion-infected mice and consistent with excitotoxicity. Phosphoproteomics analysis revealed phosphorylated glutamate receptors and calcium-sensitive kinases, including protein kinase C (PKC). Additionally, 92N-PrP^C^-expressing neurons showed persistent calcium influx as well as dendritic beading, which was rescued by an *N*-methyl-d-aspartate receptor (NMDAR) antagonist. Finally, survival of *Prnp^92N^* mice was prolonged by blocking active NMDAR channels. We propose that dysregulated PrP^C^-NMDAR–induced signaling can trigger an excitatory-inhibitory imbalance, spongiform degeneration, and neurotoxicity and that calcium dysregulation is central to PrP^C^-linked neurodegeneration.

## Abstract 2

We identify heightened neuron activity, protein kinase C activation, and NMDA receptor phosphorylation linked to rapidly progressive neurodegeneration in a genetic model of PrPC toxicity.

## Graphical abstract

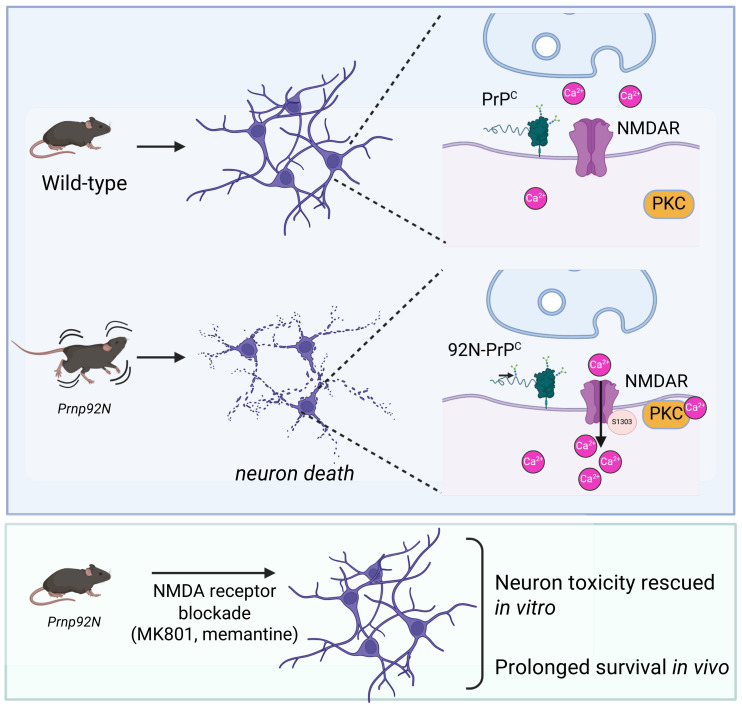

## Introduction

Extracellular l-glutamate is tightly regulated to control inter- and intraneuronal signaling in the CNS (1, 2). Increases in glutamate release or in membrane-bound glutamate receptors lower the threshold for neuronal activity and strengthen circuits. However, persistent neuronal hyperactivity from excessive extracellular glutamate causes elevated intracellular Ca^2+^ and signaling leading to excitotoxicity, which may underlie synaptic atrophy in stroke and neurodegenerative disease, including Alzheimer’s (AD), Parkinson’s (PD), and prion disease (3, 4). In neurodegenerative disease, pathogenic aggregates raise glutamate receptor activity and intracellular Ca^2+^, disrupt homeostatic glutamate signaling, and contribute to synaptic loss (5), the strongest correlate for cognitive decline in patients (6). Thus, a central goal of the field is to identify the synaptic membrane interactions and signaling pathways regulating neuronal activity.

The cellular prion protein PrP^C^ is highly expressed at the synaptic surface and binds amyloid-β (Aβ), α-synuclein (αSyn), and PrP oligomers, inducing aberrant glutamate receptor signaling (7–10). Aβ oligomers (Aβos) and αSyn oligomers bind PrP^C^ and reportedly trigger metabotropic glutamate receptor 5–driven (mGluR5-driven) activation of Fyn kinase, which phosphorylates the *N*-methyl-d-aspartate receptor (NMDAR) 2B subunit (pNR2B-Y1472), exacerbating high cytosolic Ca^2+^ and inducing dendritic spine loss (8, 11, 12). In support of PrP^C^-linked toxicity, PrP^C^-depleted AD mice resist Aβo-induced memory deficits (13), and anti-PrP^C^ antibodies in transgenic AD mice rescue synaptic loss (14). PrP aggregates (PrP^Sc^) also bind PrP^C^, yet trigger NMDARs and α-amino-3-hydroxy-5-methyl-4-isoxazoleproprionic acid receptors (AMPARs) and induce p38 MAPK activation in vitro, suggesting that activation of specific PrP^C^-linked signaling pathways is ligand dependent (9). These PrP^Sc^-activated pathways are also PrP^C^ dependent, as depleting PrP^C^ reverses clinical signs in prion-infected mice (15, 16). Since PrP^C^ has been observed to interact with NMDAR complexes (8, 17–19), PrP^Sc^-bound PrP^C^ may further affect neuronal activity through modulation of NMDAR channels. Collectively, an emerging consensus indicates that PrP^C^ engagement mediates neuronal signaling, yet the essential transmembrane binding partner(s) and downstream consequences in health and disease are unresolved and have not yet been investigated using a phosphoproteomics approach.

The importance of the intrinsically disordered amino (N-) terminus of PrP^C^ (residues 23–120) in neuronal toxicity has been demonstrated, as N-terminal PrP^C^ mutations induce large ionic currents in cultured neurons (20), and large N-terminal deletions or insertions drive neuronal death in vivo (21–23). Furthermore, fewer or additional N-terminal octapeptide repeats (ORs) cause familial prion disease in patients (24–27) and mouse models (28), and deleting the OR domain prevents neurotoxicity in brain slices (29), underscoring a role for the amino terminus in neurotoxicity. An intramolecular interaction between the N- and C-termini of PrP^C^ may function to normally suppress large inward currents, which are observed when the N-terminus is untethered and may be a source of neurotoxicity (30, 31).

To alter PrP^C^ N-terminal interactions and investigate the effect on downstream signaling, we used a genetics approach combined with phosphoproteomics to identify aberrant signaling pathways. To largely preserve the primary sequence, we exploited PrP^C^ as a glycoprotein and knocked in a single residue substitution in PrP^C^, PrP^C^-G92N, introducing an *N*-glycosylation site between the ORs (residues 51–90) and the central lysine cluster (residues 100–109), a segment postulated as crucial for prion infection (32). We predicted that an N-terminal glycan would sterically and electrostatically interrupt N-terminal domain interactions on the membrane (30, 33). Strikingly, young *Prnp^92N/92N^* mice developed a robust, highly penetrant phenotype that included myoclonic tremors, spontaneous seizures, and necrosis of hippocampal pyramidal neurons (CA1). Phosphoproteomics and Western blotting of brain revealed an early increase in phosphorylated GluN2B (pGluN2B) (S1303), a reduction in glutamate transporters (excitatory amino acid transporter 2 [EAAT2]), and an increase in kinase activity, including Ca^2+^/calmodulin-dependent protein kinase II (CaMKII) and protein kinase C (PKC), consistent with increased intracellular Ca^2+^–induced signaling. NMDA stimulation of cultured *Prnp^92N/92N^* neurons induced persistently elevated intracellular Ca^2+^. Survival of *Prnp^92N/92N^* mice was prolonged by treatment with memantine, an NMDAR antagonist. Together, the data support a model in which an N-terminal PrP^C^ mutation can drive persistently increased Ca^2+^-induced signaling events, increased NMDAR channel activity, and excitotoxicity.

## Results

### G92N-PrP^C^–knockin mice develop rapidly progressive neurologic disease.

**Figure 1:**
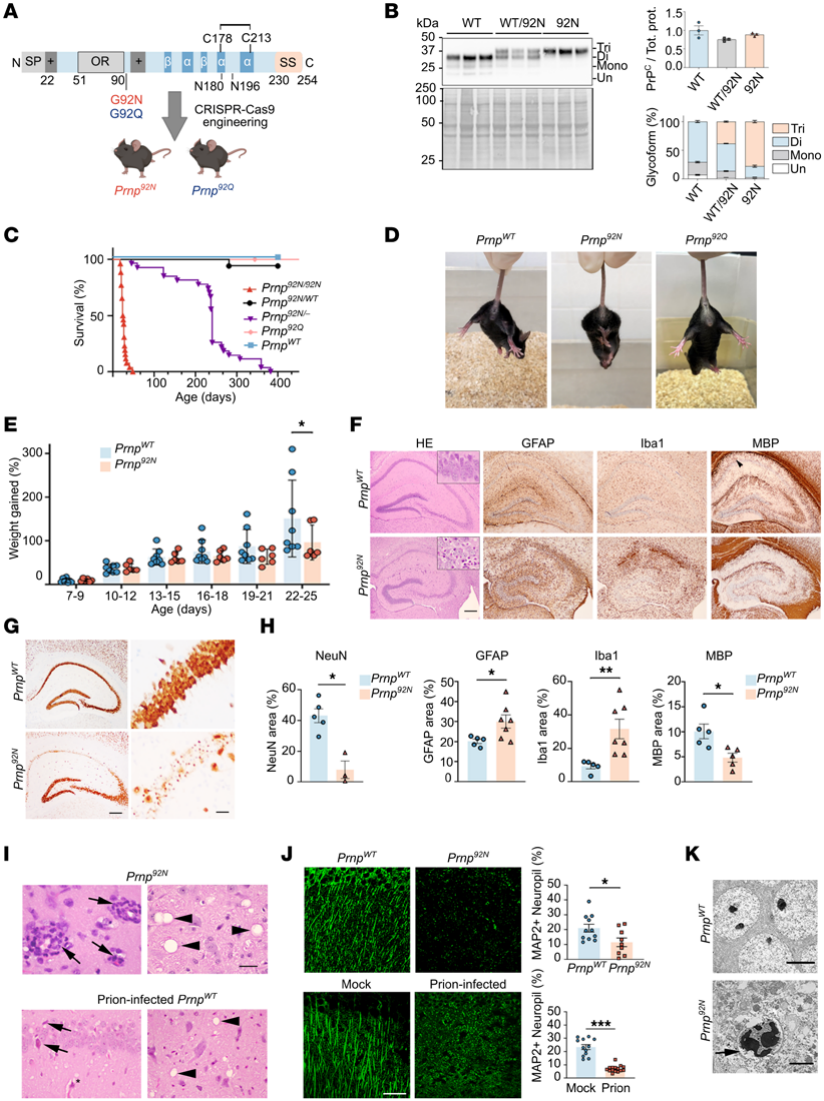
Mice expressing *Prnp^92N^* develop rapidly progressive neurologic disease with necrosis of CA1 pyramidal neurons. (**A**) Schematic diagram of linear PrP^C^ showing N- and C-terminal domains and the location of the G92N (red) or G92Q substitution (control, blue). The G92N substitution results in glycan incorporation into the flexible N-terminal domain. SP, signal peptide; OR, octapeptide repeat; SS, GPI signal sequence. The illustration in **A** was created with BioRender.com. (**B**) Western blot and quantification of PrP^C^ expression and glycoform profile in *Prnp^92N^* mice (P18–P19). Tri, triglycosylated PrP^C^ band; Di, diglycosylated PrP^C^ band; Mono, monoglycosylated PrP^C^ band; Un, unglycosylated PrP^C^ band. (**C**) Survival curve for *Prnp^WT^* (*n* = 11), *Prnp^WT/92N^* (*n* = 16), *Prnp^92N/KO^* (*n* = 28), *Prnp^92N/92N^* (*n* = 18), and *Prnp^92Q^* (*n* = 26) mice. (**D**) Hind limb clasping in a *Prnp^92N^* mouse at P24 not observed in the littermate control or in a *Prnp^92Q^* mouse. (**E**) Graph showing the weight gain in mice from 7 to 25 days of age. (**F**) *Prnp^WT^* and *Prnp^92N^* (P25) hippocampi stained with H&E or immunolabeled for astrocytes (GFAP), microglia (Iba1), or myelin (MBP) and quantification of CA1. Arrowhead indicates the region where myelin is present in *Prnp^WT^* and reduced in the P*rnp^92N^* mice. (**G**) NeuN-immunolabeled hippocampi reveal extensive CA1 neuronal loss in *Prnp^92N^* mice (P29). (**H**) NeuN area quantified from hippocampi of P24 *Prnp^WT^* and *Prnp^92N^* mice. (**I**) H&E-stained images of *Prnp^92N^brain* show extensive perivascular neutrophils in the hippocampus (arrows, left panel) and multiple dark-rimmed and septate vacuoles in the brainstem (arrowheads, right panel) at P25, while images from prion-infected mice (strain ME7) also show neuron death in the hippocampus (arrows) and vacuoles in the brainstem (arrowheads). (**J**) MAP2 labeling of hippocampus shows a similar loss of dendrite structure in *Prnp^92N^* (P25) and in prion-infected mice, as compared with *Prnp^WT^* and mock-brain-inoculated control mice, respectively. (**K**) Representative TEM images of CA1 pyramidal neurons from age-matched *Prnp^WT^* and terminal *Prnp^92N^* mice. Arrow shows condensed chromatin. All results are shown as the mean ± SEM. One- or 2-way ANOVA with Tukey’s multiple-comparison test was performed to determine statistical significance (**B**, for PrP^C^ levels and glycoform, respectively). The statistically significant differences were as follows: unglycosylated *Prnp^WT^* versus *Prnp^92N^*, ***P < 0.001 and versus *Prnp^WT/92N^*, *P < 0.05; monoglycosylated *Prnp^WT^* versus *Prnp^WT/92N^*, and versus *Prnp^92N^*, and *Prnp^WT/92N^* versus *Prnp^92N^*, ****P < 0.0001, ; diglycosylated *Prnp^WT^* versus *Prnp^WT/92N^* and *Prnp^92N^*, and *Prnp^WT/92N^* versus *Prnp^92N^*, ****P < 0.0001. Scale bars: 500 μm (**F** and **G** [left]), 50 μm (**G** [right], **I**, and **J**), and 5 μm and 2 μm (**K**, top and bottom, respectively).

The N-terminus of PrP^C^ is an intrinsically disordered region (IDR) and contains motifs important for protein interactions and cell signaling events (34). Thus, we reasoned that an N-terminal glycan would affect PrP interactions. We modified the IDR with a single amino acid mutation (glycine to asparagine) to code for an *N*-linked glycan at position 92 (sequon amino acid sequence: NGT), following the final octapeptide repeat (Figure 1A), and verified the third glycan addition in RK13 cells (Supplemental Figure 1A; supplemental material available online with this article; https://doi.org/10.1172/JCI186432DS1). Using CRISPR/Cas9, we then generated *Prnp^92N^* mice (C57BL/6NJ × B6SJLF1) backcrossed with C57BL/6J WT mice, and interbred heterozygous mice or bred mice with *Prnp^–/–^* (35) mice to generate *Prnp^92N/92N^*, *Prnp^92N/WT^*, *Prnp^92N/–^*, and *Prnp^WT/WT^* mice. PrP^C^ expression was similar in age-matched *Prnp^92N/92N^* and *Prnp^WT/WT^* brains (hereafter referred to as *Prnp^92N^* and *Prnp^WT^*) (Figure 1B and Supplemental Figure 1, B–D), and the 92N-PrP^C^ showed high glycan occupancy at all 3 glycan sites, as more than 70% of PrP^C^ was triglycosylated (Figure 1B). Following glycan cleavage by peptide-*N*-glycosidase F (PNGase F) digestion, PrP^C^ resolved as a single unglycosylated PrP band (Supplemental Figure 1C). Finally, 92N-PrP^C^ was distributed similarly in the *Prnp^92N^* and *Prnp^WT^* brains (Supplemental Figure 1D), with the highest expression in the hippocampus (CA1). *Prnp^92Q/92Q^*-knockin (referred to hereafter as *Prnp^92Q^*) control mice were also generated, as glutamine is structurally similar to asparagine, yet does not code for glycan attachment (Figure 1, A, C, and D).

Mutations can alter PrP^C^ biosynthesis and trafficking (36, 37), therefore, we next evaluated whether the third glycan affected the cleavage, localization, or degradation of PrP^C^. There was no difference in the ADAM10-cleaved or total PrP^C^ levels in cerebral cortex, suggesting that WT and 92N-PrP^C^ were similarly expressed and cleaved by ADAM10 at the cell surface (Supplemental Figure 1B). To determine whether 92N-PrP^C^ localized to lipid rafts, we isolated detergent-resistant membranes (DRMs) from cerebral cortex and found that WT and 92N-PrP^C^ similarly occupied flotillin-positive DRM fractions (Supplemental Figure 1E). We further assessed PrP^C^ cell-surface localization in cultured cortical neurons using phospholipase C (PIPLC) to cleave the GPI anchor. We detected variable 92N-PrP^C^ levels in the media and low levels in the cell lysate, possibly due to poor viability of *Prnp^92N^* neurons after 18 days in culture (Supplemental Figure 1F). To circumvent any neuronal toxicity effects, we further probed PrP^C^ cell-surface levels in WT-PrP^C^–, 92Q-PrP^C^–, and 92N-PrP^C^–transfected RK13 cells (PrP^C^-deficient) by flow cytometry, and found PrP^C^ levels to be similar (Supplemental Figure 1G). Furthermore, we found no difference in the PrP^C^ degradation kinetics in WT-PrP^C^– and 92N-PrP^C^–expressing RK13 cells treated with cycloheximide (Supplemental Figure 1H). Collectively, the in vitro and in vivo data support the idea that 92N-PrP^C^ was trafficked and processed similarly to WT-PrP^C^.

The *Prnp^92N^* mice appeared clinically indistinguishable from *Prnp^WT^* mice until approximately P22, when the *Prnp^92N^* mice showed reduced weight gain (Figure 1E). Starting from P17 to P25, *Prnp^92N^* mice developed a severe, rapidly progressive neurological phenotype (seizures, hind leg clasp, ataxia, paraparesis, and myoclonic tremors) and were terminal by P26 (26 ± 1 day, mean ± SEM) with 100% penetrance (Figure 1, C and D, and Supplemental Videos 1–3). Hemizygous mice (*Prnp^92N/-^*) survived to approximately P230 (234 ± 15 days) (Figure 1C), indicating a dose effect. The phenotype was rescued by the presence of WT-PrP^C^, as the *Prnp^92N/WT^* mice survived for more than 400 days, suggesting that WT-PrP^C^ was protective against 92N-PrP^C^ effects (Figure 1C). *Prnp^92Q^* mice also survived more than 500 days and showed no clinical signs or brain lesions (Figure 1, C and D, and Supplemental Figure 2), suggesting that the N-terminal glycan, and not loss of G92, played a role in disease development.

### Prnp^92N^ mice lack PrP aggregates and infectious prions yet show severe hippocampal pathology and spongiform degeneration.

To determine whether *Prnp^92N^* formed aggregates, we first assessed the terminal *Prnp^92N^* whole brain for proteinase K–resistant (PK-resistant) or insoluble PrP^C^ following ultracentrifugation. We found that 92N-PrP^C^ was as PK sensitive and soluble as WT-PrP^C^ (Supplemental Figure 3, A and B). To then evaluate whether the 92N-PrP^C^ showed seeding activity or transmissibility to mice, we tested terminal *Prnp^92N^* brain by real-time quaking-induced conversion (RT-QuIC) (38) (Supplemental Figure 3C) and by inoculating *Prnp^92N^* brain homogenate into *Prnp^WT^* mice, respectively, which did not reveal evidence of seeding activity or infectivity. All mice survived for more than 500 days post inoculation (DPI), with no biochemical evidence of prion infection (Supplemental Figure 3, D and E). Thus, *Prnp^92N^* mice developed neurologic disease in the absence of aggregates or infectivity, uncoupling aggregation and neurotoxicity.

To investigate the neuropathologic phenotype in the terminal *Prnp^92N^* mice, we characterized the lesions over time in the brain and spinal cord. At terminal disease, we observed scattered vacuoles in the basal ganglia, cerebral cortex, thalamus, cerebral peduncle, brainstem, and spinal cord. However, the most severe lesions were in the hippocampal CA1 (the region with highest PrP^C^ expression; Supplemental Figure 1D), where the NeuN^+^ pyramidal neurons were shrunken and pale with pyknotic nuclei (Figure 1, F–H) reminiscent of the CA1 neuronal lesions in mice infected with mouse-adapted prions (ME7 strain) (Figure 1I). There were also neutrophils within and surrounding vessel walls, occasional vacuoles, myelin loss, and widespread astrocytic degeneration and reactive microglia (Figure 1, F and I). 

To determine whether synapse numbers were altered, we immunolabeled and quantified presynapses (synaptophysin) and dendritic microtubule–associated protein 2 (MAP2) in P10, P20, and P25 hippocampi (CA1). While synapse numbers were reduced by P20, dendritic MAP2 was not reduced until P25 (Figure 1I and Supplemental Figure 4). Prion-infected mice also showed reduced MAP2 (strain ME7) (Figure 1J), consistent with altered dendrite structure as previously described (39, 40). Thus the amino-terminal PrP substitution induced an excitatory clinical phenotype and an early-onset neurodegeneration characterized by synapse loss, neuronal death, astro- and microgliosis, and spongiform encephalopathy, with the most severe lesions in the hippocampus (CA1), cerebral cortex, thalamus, and brainstem.

Given the seizures and death of CA1 neurons in *Prnp^92N^* mice, we next sought to determine whether hippocampi from terminal *Prnp^92N^* mice showed evidence of excitotoxicity using transmission electron microscopy (TEM). Ultrastructure of the CA1 pyramidal neurons revealed extensive somatodendritic swelling, irregular, condensed, and vacuolated chromatin, and leakage of cytoplasmic contents into the extracellular space in most CA1 neurons (Figure 1K), indicative of neuronal necrosis and consistent with excitotoxic cell death or ischemia (41). No WT neurons in CA1 showed these phenotypes (Figure 1KJ).

To test whether loss of inhibitory interneurons was contributing to the excitatory phenotype, we quantified parvalbumin-positive (PV-positive) and somatostatin-positive (SST-positive) inhibitory interneurons in the hippocampus and motor cortex. However, we observed no differences at P20 or P25 (Supplemental Figures 5 and 6), indicating that these PV and SST interneurons that gate synaptic plasticity remained preserved. Interestingly, by P20, there was an increase in vesicular GABA transporter–positive (VGAT^+^) immunostained area (presynaptic). Additionally, the gephyrin-positive (GEPH^+^) area (postsynaptic) was significantly increased, suggestive of a compensatory response (Supplemental Figure 7).

### 92N-PrP^C^ toxicity is neuronally driven, cell autonomous, and degenerative.

**Figure 2:**
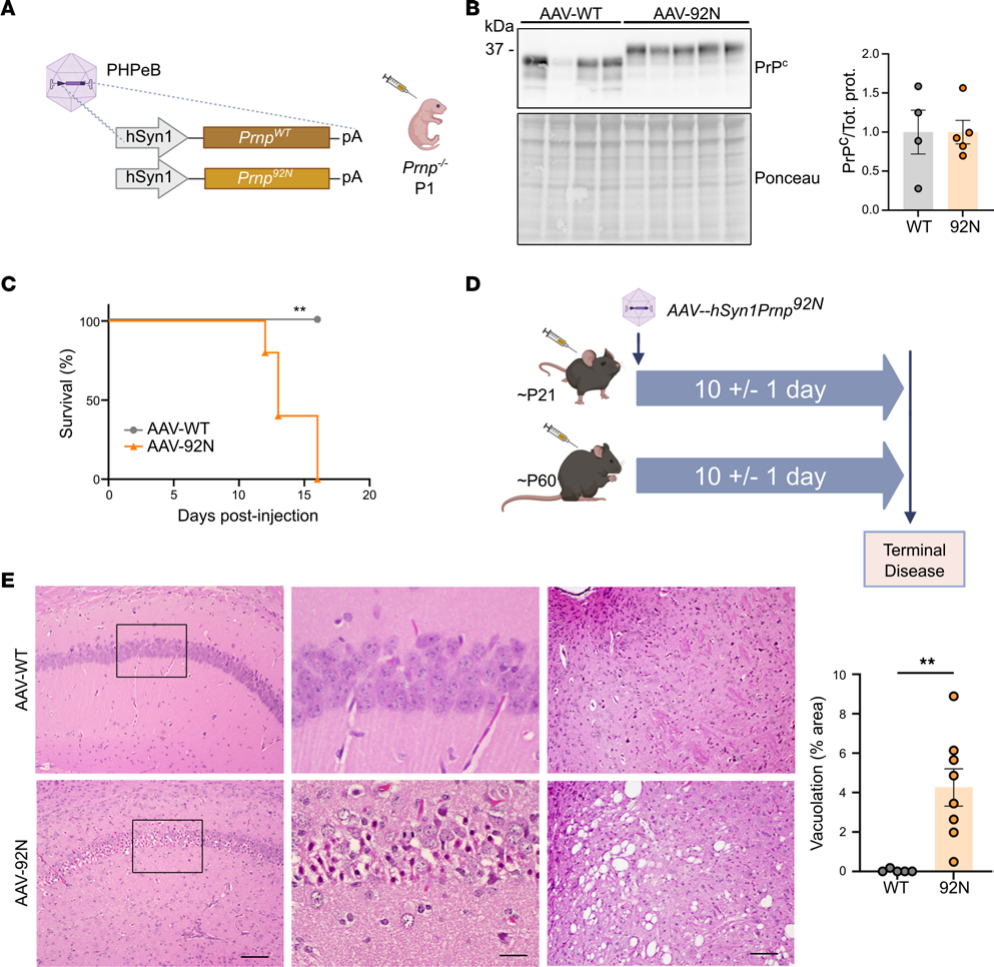
Targeting *Prnp^92N^* exclusively to neurons induces rapid neurodegeneration in mice. (**A**) Experimental design of the AAV constructs and i.v. injection into the superficial facial vein in P1 *Prnp^–/–^* mice. (**B**) Western blot and quantification of 92N-PrP^C^ and WT-PrP^C^ in brain lysates. (**C**) Survival curve for 92N-PrP^C^– and WT-PrP^C^–expressing mice (*n* = 4 and 5 for AAV-WT and AAV-92N, respectively). (**D**) Approximate age and survival of juvenile and adult *Prnp^–/–^* mice transduced with AAV-*hSyn1Prnp^92N^* (*n* = 4 and 3 per genotype for the juvenile and adult groups, respectively). Mice transduced with AAV-*hSyn1Prnp^WT^* did not show clinical signs. (**E**) Representative images of H&E-stained sections of hippocampus (CA1) (left and middle panels) and brainstem (right) show CA1 hippocampal necrosis and spongiform changes in 92N-PrP^C^–expressing mice. The boxed region in CA1 is shown at higher magnification (middle panel). Scale bars: 200 μm (left, right) and 50 μm (middle). Bar graphs show the mean ± SEM. **P < 0.01, by unpaired, 2-tailed *t* test with Welch’s correction (**B**), log-rank (Mantel-Cox) test (**C**), and Mann-Whitney *U* test (**E**). Illustrations in **A** and **D** were created with BioRender.com.

PrP^C^ is ubiquitously expressed, and whether mutant PrP^C^ expression in neurons drives the disease, as shown in prion infection (15), or requires expression by other cells is unclear. To determine whether the neurotoxicity proceeds in a cell-autonomous manner, we transduced neonatal (P1) *Prnp^–/–^* mice with adeno-associated virus (AAV) *Prnp^92N^* or AAV-*Prnp^WT^* under a neuron-specific synapsin 1 promoter (AAVhSyn1-92NPrP^C^ or AAVhSyn1-WTPrP^C^) (Figure 2, A and B). All 92N-PrP^C^–expressing, but not WT-PrP^C^–expressing, mice developed behavioral arrest, myoclonic tremors, and kyphosis, indicating terminal disease, at 14 DPI (± 2 days) (Figure 2C).

To next determine whether mutant PrP^C^ induced a degenerative or developmental disease, we injected AAV-*Prnp^92N^* into juvenile or adult mice (P21–P22 and P49–P66). Remarkably, all AAVhSyn1-92NPrP^C^–inoculated mice developed terminal neurotoxicity at approximately 10 DPI (±1 day) (Figure 2D, Supplemental Figure 8, and Supplemental Videos 4 and 5). There was severe focal spongiform degeneration in the brainstem and in some cases hippocampal or midbrain neuronal necrosis (Figure 2E), indicating that 92N-PrP^C^ expression exclusively in neurons was sufficient for neurotoxicity. Additionally, toxicity occurred independent of age.

Since astrocytes express PrP^C^ and could be a driver of or contributor to neuronal toxicity, we also tested AAV-*Prnp^92N^* or AAV-*Prnp^WT^* under an astrocyte-specific GfaABC1D promoter (42) in adult mice (approximately P60). All mice survived with no clinical signs to 150 DPI, suggesting that astrocyte expression of 92N-PrP^C^ did not induce neurologic signs, although PrP^C^ expression may have been lower (Supplemental Figure 9). Thus, 92N-PrP^C^ caused a cell-autonomous, neurodegenerative disease driven by neuronal expression of mutant PrP^C^.

### Addition of a third glycan in the PrP N-terminus significantly alters brain phosphoproteome.

**Figure 3:**
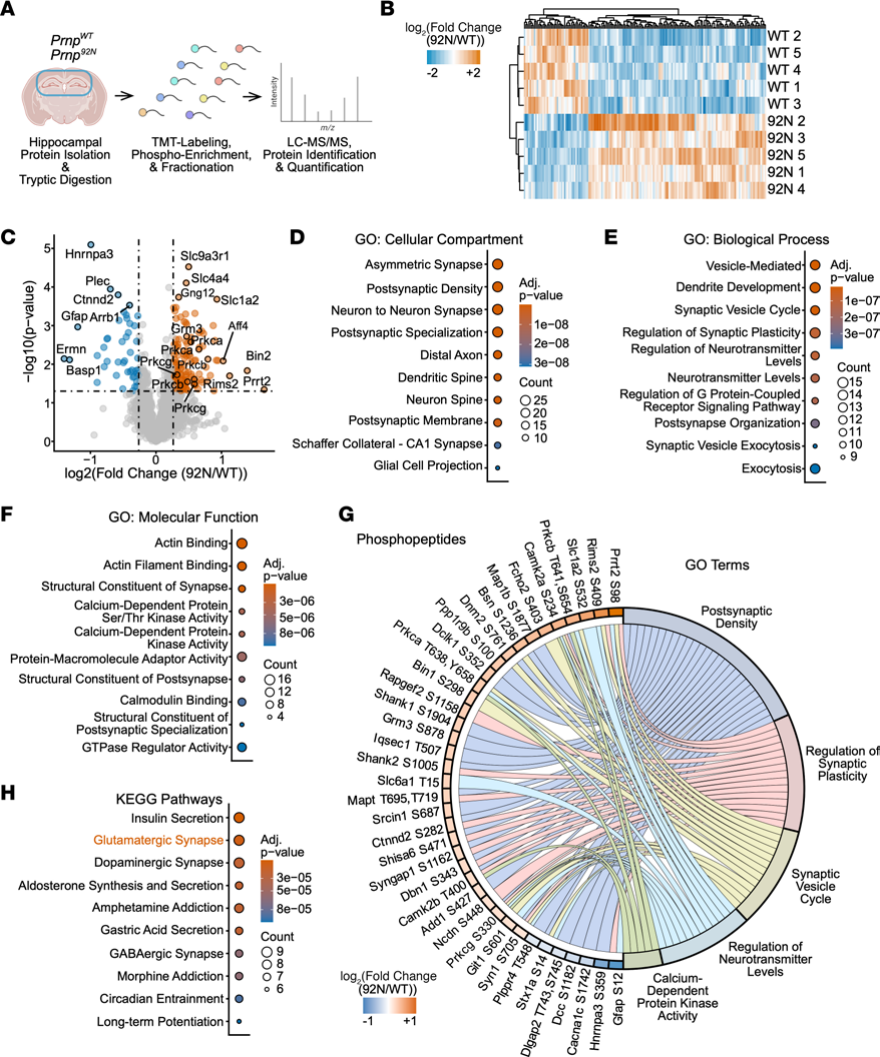
Phosphoproteomics analysis reveals altered glutamatergic signaling peptides in *Prnp^92N^* hippocampus. (**A**) Experimental design of phosphoproteomics analysis of hippocampus (*n* = 5 per genotype). (**B**) Clustered heatmap of significantly altered peptides identified in *Prnp^92N^* versus *Prnp^WT^* hippocampus. (**C**) Volcano plot of all detected phosphopeptides. Dashed lines indicate fold change (>1.2) and *P* value cutoffs (*P* < 0.05). Select altered peptides are indicated by the protein’s gene symbol. (**D**–**F**) GO enrichment analysis shows the top 10 most enriched cellular component, biological process, or molecular function ontologies. Circle color and size indicate the significance and gene count, respectively. (**G**) Chord plot for selected GO terms and associated phosphopeptides. Gene symbols and phosphorylation sites are indicated. (**H**) Top 10 affected KEGG pathways.

Given that the *Prnp^92N^* mice develop a rapidly progressive clinical phenotype and widespread neuronal necrosis, we next assessed mice for disrupted cell signaling by analyzing the phosphoproteome and total proteome in preclinical mice (P20) using tandem mass tag (TMT) mass spectrometry (all groups, *n* = 5 each). We measured phosphorylated and total protein levels in 3 tissue subsets: globally in the whole brain, regionally in the hippocampus, and focally in the neuronal synaptosome (total proteome only). For all data subsets, we used the criteria of a fold change of greater than 1.2 and a *P* value of less than 0.05. WT and 92N data clustered based on differences in phosphopeptides (Figure 3, A and B, Supplemental Figures 10–13, and Supplemental Tables 1 and 2).

In whole-brain samples, we identified a total of 1,084 unique phosphopeptides, 48 of which were differentially abundant from 46 proteins, the majority of which were hypophosphorylated (14 hyperphosphorylated and 34 hypophosphorylated). *Prnp^92N^* and *Prnp^WT^* groups clustered separately, with good reproducibility among the replicates (Supplemental Figure 10). A Gene Ontology (GO) enrichment analysis revealed the top GO terms as structural constituents of postsynaptic density (molecular function) and postsynaptic density (cellular compartment) (Supplemental Figure 10). Notably, there was altered phosphorylation of discs large scaffold proteins Dlg2 and Dlg4 and discs large associated protein Dlgap1, which bind to NMDARs and AMPARs in the postsynaptic density (PSD) and contribute to synaptic scaling. Dlg4 (also known as PSD95) interacts with the cytoplasmic tail of NMDAR (43). Phosphorylation of NMDAR subunit 2B at the C-terminal serine 1303 (S1303) increases NMDAR current (44, 45) or reduces NMDAR desensitization (46), but did not differ significantly (*P* = 0.07, normalized to total). Among the differentially altered unmodified peptides (37 proteins: 20 increased and 17 decreased), the glutamate uptake proteins EAAT1 (*Slc1a3*) and EAAT2 (*Slc1a2*) were among the most significantly reduced in the *Prnp^92N^* mice (Supplemental Figure 11).

Given that regional phosphoprotein signatures may be diluted in a whole-brain analysis, we next performed a hippocampal phosphoproteome (P20) analysis, which revealed 2,200 unique phosphopeptides, 169 (from 126 different proteins) of which were differentially abundant (Figure 3B). The majority (73%) of phosphoprotein changes were due to hyperphosphorylation (124 hyper- and 45 hypophosphorylated peptides) (Figure 3C). In the GO analysis, PSD was among the most significant cellular compartments indicated (Figure 3D), and 2 of the most significant biological processes altered were dendrite development and regulation of synaptic plasticity, with regulation of neurotransmitter levels, neurotransmitter transport, and postsynapse organization also among the top 10 (Figure 3E). Notably, calmodulin binding and Ca^2+^-dependent protein kinase activity (Figure 3F) were among the top 10 molecular functions altered. Prominently altered Ca^2+^-dependent kinases included CaMKIIα and CaMKIIβ as well as PKCα, PKCβ, and PKCγ, all of which were hyperphosphorylated (Figure 3G). Finally, Kyoto Encyclopedia of Genes and Genomes (KEGG) pathway analysis revealed the glutamatergic synapse as the second most significant pathway (insulin secretion was first) (Figure 3H). Thus, the hippocampal phosphoproteome changes indicated glutamatergic synapses and Ca^2+^ kinases as being among the most significant. Proteins with significantly altered phosphorylation sites also interrelate through prominent ontologies, suggesting 92N-PrP^C^ impinges upon these processes through a suite of interrelated proteins that include Ca^2+^-dependent kinases (genes: *Prkca*, *Prkcb*, *Prkg*, *Camk2a*) as well as proteins that regulate neurotransmitter levels and synaptic plasticity at the PSD (genes: *Shank1*, *Syngap1*, *Syn1*, *Slc6a1*) (Figure 3G).

Over 4,086 unmodified proteins were identified in the hippocampal dataset, with 870 unique proteins (including splice variants) being significantly differentially expressed and the majority increased in the *Prnp^92N^* group (636 increased and 234 decreased) (Supplemental Figure 12). Notably, there were again major reductions in the glutamate transporters EAAT1 (*Slc1a3*) and EAAT2 (*Slc1a2*) (top 30 by significance). GluN1, PKCα, PKCβ, and PKCγ peptides were reduced in the *Prnp^92N^* hippocampus, whereas GluN2B and CaMKIIα and β peptides were unchanged (*P* > 0.05).

With the glutamatergic synapse and Ca^2+^ kinases as top phosphoproteome hits, we further interrogated the synapse by purifying the cortical synaptosomes and analyzing the proteome for alterations in synaptic receptors or other transporters. There were 3,587 unique proteins identified, with 293 differentially expressed proteins (100 increased and 193 decreased) (Supplemental Figure 13). The GO analysis revealed that the most altered molecular function was active transmembrane transporter activity, reflecting a reduction in EAAT1 and EAAT2 that were reduced by approximately 70%, as well as sodium (slc) transporters. Notably, there were no differences in the abundance of PrP or glutamate receptor peptides at the synapse, including GluN1, GluN2A, GluN2B, GluN2D, GluA1, GluA2, and mGluR5, or in CaMKIIα, CaMKIIβ, PKCα, PKCβ, and PKCγ, suggesting that protein trafficking to the synapse was unaltered. Together, the proteomics data revealed striking differences in phosphorylated and unmodified peptides, including glutamate receptors, glutamate transporters, and Ca^2+^ kinases, and suggest that the most significant differences were in Ca^2+^-mediated excitatory synaptic signaling at the postsynapse.

### Prnp^92N^ mice exhibit alterations in proteins that mediate glutamate signaling.

**Figure 4:**
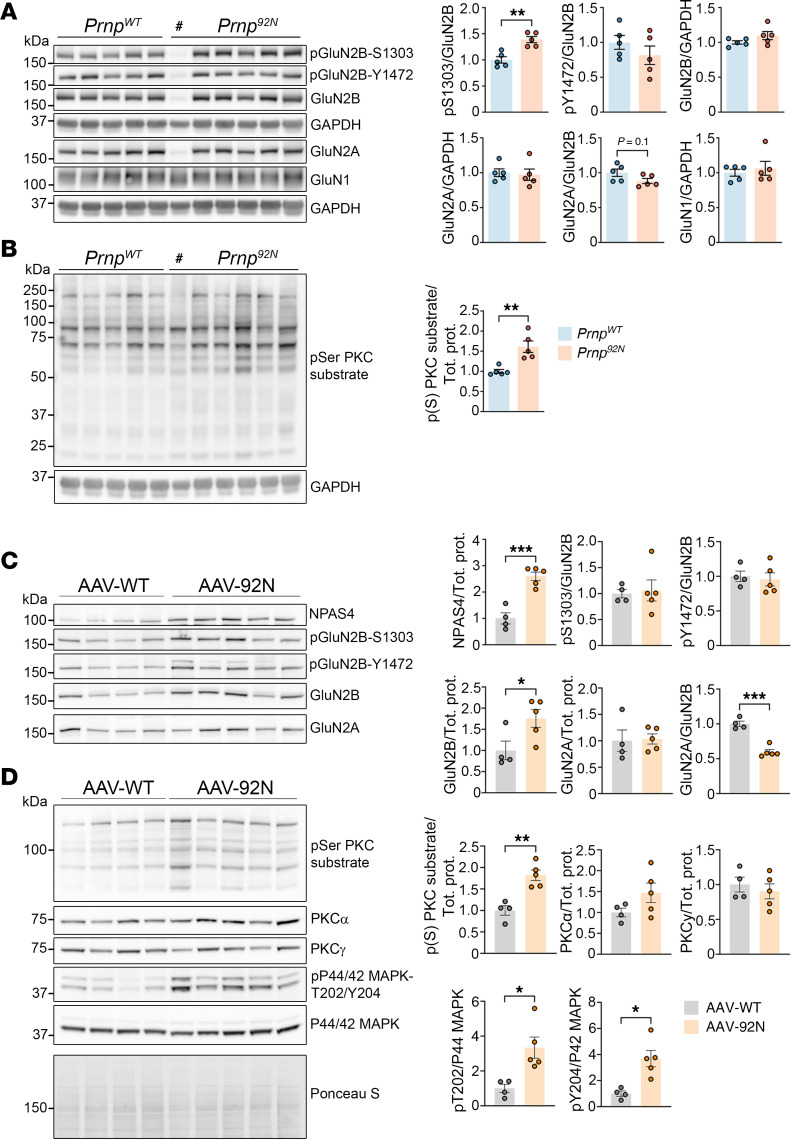
Increased neuronal activity and glutamatergic signaling proteins in *Prnp^92N^* and AAV-92NPrP–transduced hippocampus. (**A**) Western blots and quantification of pGluN2B, NMDAR subunits and (**B**) PKC substrates in *Prnp^WT^* and *Prnp^92N^* hippocampus (P20, male and female mice). (**C**) Western blots and quantification of AAV-*hSyn1Prnp^WT^* and -*hSyn1Prnp^92N^* hippocampus (AAV.PHPeB.hSyn1-WT and AAV.PHPeB.hSyn1–92N-PrP) for Npas4, pGluN2B, NMDAR subunits, and (**D**) PKC phosphorylated substrates and calcium-sensitive protein kinases. Pound symbol indicates that the mouse was moribund, had severe hippocampal necrosis, and was an outlier and was therefore omitted from the data in the graphs. Groups of *n* = 5 *Prnp^WT^* and *Prnp^92N^* mice, and *n* = 4 and 5 AAV-WT and AAV-92N, respectively. Data represent the mean ± SEM. *P < 0.05, **P < 0.01, and ***P < 0.001, by unpaired, 2-tailed *t* test with Welch’s correction.

Given that the phosphoproteomics data showed increased CaMKII and PKC phosphorylation at P20, we next evaluated synaptic receptors and kinases in the hippocampus over time, starting at P10–P11, when microgliosis was evident but mild (Supplemental Figure 14). pGluN2B-S1303 levels showed no difference at P10 (*P* = 0.08), however, they were significantly increased by P15–P16 and remained increased at P20, suggestive of increased Ca^2+^ flux through the NMDARs starting in young, preclinical mice (Figure 4A and Supplemental Figures 15 and 16). pGluN2B-Y1472 levels were unaltered (Figure 4A and Supplemental Figures 15 and 16). Levels of NMDAR subunits showed variability with age; GluN2A was increased at P10 and P15, but unchanged at P20. There were no changes in the GluN2A/GluN2B ratio over time (Figure 4A and Supplemental Figures 15 and 16).

GluN2B is phosphorylated at S1303 by the CaM kinase family members CaMKII or death-associated protein kinase 1 (DAPK1) (45, 47–49), or by PKC (50). PKC and CaMKII were differentially abundant by liquid chromatography/mass spectrometry (LC/MS), therefore, we next measured phosphorylation of pan-PKC substrates as well as pCaMKII (α and β) (T286/287) as a proxy for activity. While pCaMKIIα and -β levels were unchanged, pPKC substrates were increased at P10 and at P20, suggesting heightened activity of the Ca^2+^ sensitive kinase PKC starting from an early age (Figure 4B and Supplemental Figures 15 and 16). pERK is downstream of PKC, and was also increased at P10 and P15, suggesting activation of a signaling cascade (Supplemental Figures 15 and 16).

Concurrent with the increase in pGluN2B, the astrocytic glutamate transporter EAAT2 was markedly reduced (by approximately half) by P15, suggestive of an early reduction in glutamate transporters (Supplemental Figure 16). The microglial response was increased starting at P10, as measured by ionized calcium–binding adaptor molecule 1 (Iba1) (Supplemental Figures 15 and 16), consistent with the histologic findings (Supplemental Figure 12).

The AAVhSyn1-92NPrP^C^– or AAVhSyn1-WTPrP^C^–transduced mice (P1) also showed markedly altered divergent hippocampal protein levels. Neuronal PAS domain protein 4 (Npas4), an inducible early gene and excitation-coupled, Ca^2+^-dependent transcription factor, was markedly increased (2.6-fold). GluN2B (Y1472) phosphorylation was not altered, yet total GluN2B was significantly increased (Figure 4C). Notably, pPKC substrates and pERK (pMAPK44/42) were increased by 1.8-fold and 3.3-fold, respectively (Figure 4D). Collectively, the data suggest that exclusive neuronal 92N-PrP^C^ expression induced excitation and increased Ca^2+^ sufficient to activate PKC and drive ERK phosphorylation.

To compare these findings with infectious prion disease, we inoculated mice with prions (strain 22L) and collected brains at a late but preterminal stage (80% of the incubation period). Here, we found that PKC substrates were elevated in the cerebral cortex, indicating for the first time heightened PKC activity in prion disease (Supplemental Figure 17). Additionally, pGluN2B (S1303) levels were elevated (Supplemental Figure 17). Together, these results suggest an overlap in Ca^2+^-sensitive kinase activation in 92N-PrP^C^– and PrP^Sc^-mediated toxicity.

### PrP^C^ and NMDAR localize to detergent resistant membranes.

**Figure 5:**
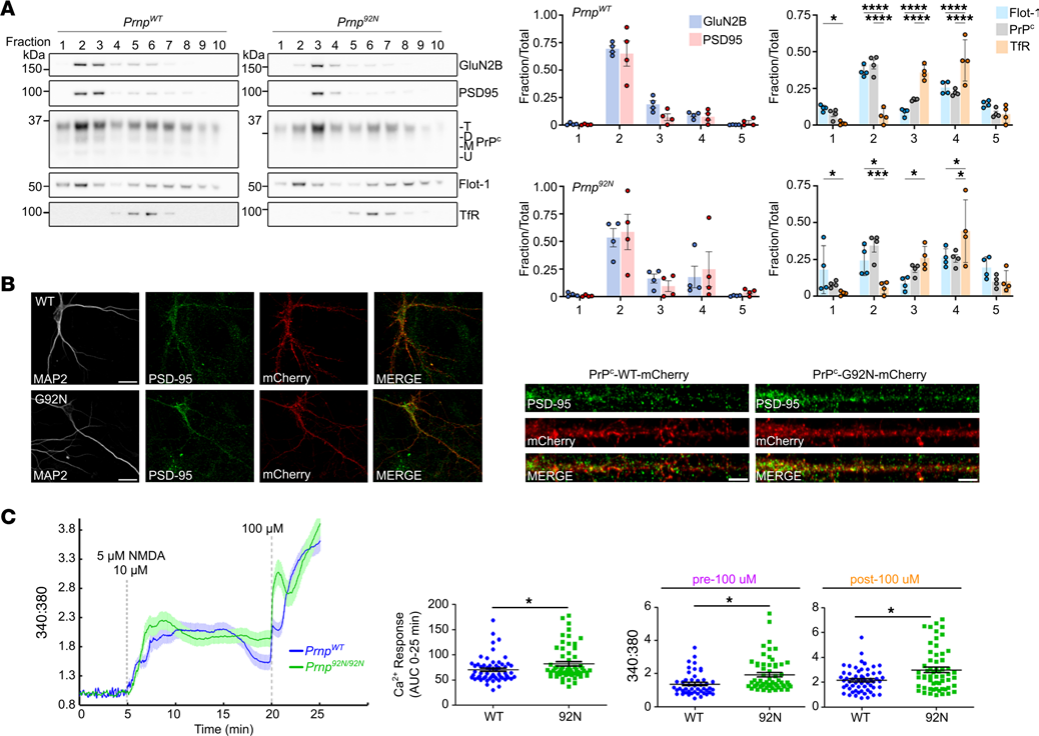
PrP^C^ and NMDAR localization and calcium response to NMDA in primary cortical neurons. (**A**) Western blot of step gradient fractions from least to most dense (fraction 10) for GluN2B, PSD95, PrP^C^, as well as flotillin 1 (Flot-1) to identify fractions containing DRMs and the transferrin receptor (TfR) as a non-DRM protein control. Fraction 1 is denoted as the first fraction with a detectable amount of flotillin 1 (higher fractions are not shown). Graph *x*-axis labels for fractions 2–5 indicate the sum of the fraction blot intensity: fractions 2 and 3 (“2”), 4 and 5 (“3”), 6 and 7 (“4”), and 8 and 9 (“5”). *n* = 4 per genotype. (**B**) Primary rat neurons expressing mCherry-tagged WT-PrP^C^ or 92N-PrP^C^ immunolabeled for MAP2 (dendrites) and PSD95 (postsynaptic density) show newly expressed PrP^C^ along dendrites. Straightened dendrites (right) show PSD95 and WT-PrP^C^ or 92N-PrP^C^ on spines. Scale bars: 20 μm (neurons) and 5 μm (dendrites). (**C**) Cortical neurons (DIV 14–16) loaded with Fura-2 AM were stimulated with low NMDA (5 μM) followed by saturating NMDA (100 μM). Graph (left) depicts the mean ± SEM for relative changes in cytosolic calcium concentration (normalized 340:380 nm ratio) over time. Quantifications show the AUC from 0–25 minutes (middle) or the mean 340:380 ratio ± SEM (right). *n* = 58 or 47 cells from 6 (*Prnp^92N^*) or 7 (*Prnp^WT^*) mice, respectively, from 4 (*Prnp^WT^*) to 5 (*Prnp^92N^*) separate experiments. *P < 0.05, ***P < 0.001, and ****P < 0.0001, by 2-way ANOVA with Tukey’s multiple-comparison test (**A**) and unpaired, 2-tailed *t* test with Welch’s correction (**C**).

PrP^C^ may closely interact with the NMDAR and modulate channel activity. To first determine whether PrP^C^ and NMDAR similarly localize within DRMs, we isolated DRMs from *Prnp^WT^* and *Prnp^92N^* brains. We found that WT-PrP^C^ and 92N-PrP^C^, PSD95, and GluN2B localized to DRMs, while the transferrin receptor (negative control) localized to detergent-soluble fractions (Figure 5A).

To further assess the neuronal localization of 92N-PrP^C^, we transfected rat neurons with mCherry-tagged *Prnp^WT^* or *Prnp^92N^* (PrP^C^-mCherry). Both WT-PrP^C^ and 92N-PrP^C^ were distributed throughout the neuron, in both soma and dendritic compartments. Strikingly, PrP^C^ clustered as puncta at or near synapses, as indicated by the juxtaposition to PSD95 (Figure 5B). Together, these data indicate that synaptic WT-PrP^C^ and 92N-PrP^C^ co-resided in lipid rafts together with the NMDAR and PSD95.

### Increased Ca^2+^ response in NMDA-stimulated PrP^92N^-expressing cortical neurons.

Given the evidence for excitotoxicity and a Ca^2+^-linked kinase pathway, together with the elevated pGluN2B (S1303) levels, we next directly tested NMDA-mediated Ca^2+^ influx in *Prnp^92N^* and *Prnp^WT^* control mouse neurons using live-cell imaging. Cortical neurons (14 days in vitro [DIV]) in magnesium-free buffer were loaded with Fura-2, and the relative NMDA-induced cytosolic Ca^2+^ response was measured. Low concentrations of NMDA and glycine were used to avoid receptor internalization (51–53). The addition of NMDA (5 μM) and glycine (10 μM) led to approximately 50% NMDA receptor activation in both *Prnp^WT^* and *Prnp^92N^* neurons (Figure 5C). After 15 minutes, the *Prnp^WT^* neurons exhibited a decay in cytosolic Ca^2+^ not observed in the *Prnp^92N^* neurons, consistent with tonically open NMDARs in the *Prnp^92N^* neurons (Figure 5C, before 100 μM NMDA; *P* = 0.002). After exposure to high concentrations of NMDA (100 μM), the *Prnp^92N^* neurons showed a larger Ca^2+^ spike than did *Prnp^WT^* neurons, collectively suggestive of increased NMDAR-mediated cytosolic Ca^2+^ (Figure 5C, after 100 μM NMDA; *P* = 0.001). The overall Ca^2+^ response to NMDA stimulation, measured as the AUC for the entire experiment (0–25 minutes), revealed a significant increase in NMDA-induced cytosolic Ca^2+^ in *Prnp^92N^* neurons compared with *Prnp^WT^* neurons (*P* = 0.03).

### Prnp^92N^ neurons show normal AMPA- and NMDA-evoked responses, but a faster NMDA current decay time.

**Figure 6:**
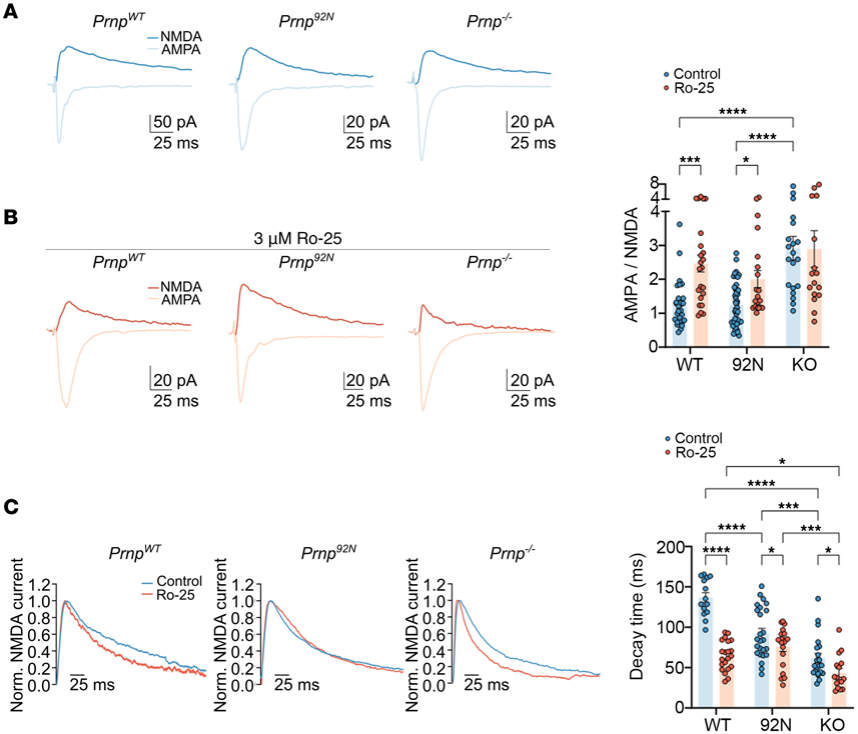
*Prnp^92N^* neurons are insensitive to a GluN2B allosteric modulator on evoked AMPAR and NMDAR currents. (**A** and **B**) The evoked AMPAR and NMDAR excitatory postsynaptic currents (EPSCs) of *Prnp^WT^*, *Prnp^92N^*, and *Prnp^–/–^* CA1 pyramidal neurons in organotypic hippocampal slices in the absence and presence of Ro-25. AMPAR EPSCs and NMDAR EPSCs were induced by synaptic stimulation at the holding potential of –60 and +40 mV, respectively. Bar graph shows the ratio of AMPAR EPSCs to NMDAR EPSCs. (**C**) Normalized NMDAR EPSCs of *Prnp^WT^*, *Prnp^92N^*, and *Prnp^–/–^* CA1 pyramidal neurons show the effect of Ro-25 on the decay time of NMDAR currents. Data represent the mean ± SEM. (**A**) *n* = 29, 43, and 20 *Prnp^WT^*, *Prnp^92N^*, and *Prnp^–/–^*, respectively; (**B**) *n* = 23, 20, and 16 *Prnp^WT^*, *Prnp^92N^*, and *Prnp^–/–^*, respectively; (**C**) *n* = 14, 25, and 21 (untreated) and *n* = 21, 19, and 16 (Ro-25 treated) *Prnp^WT^,* *Prnp^92N^* and *Prnp^–/–^*. *P < 0.05, **P < 0.01, ***P < 0.001, and ****P < 0.0001, by 2-way ANOVA with Tukey’s multiple-comparison test.

To next determine whether 92N-expressing neurons show functional differences in neuronal activity, we conducted electrophysiology experiments on organotypic brain slices from *Prnp^92N^* and *Prnp^WT^* littermates as well as from *Prnp^–/–^* mice, measuring evoked AMPAR and NMDAR currents. We found that *Prnp^–/–^* mice had a high AMPA/NMDA ratio at baseline, yet there were no differences between *Prnp^92N^* and *Prnp^WT^* mice (Figure 6A), suggesting that the magnitude of the synaptic NMDAR function was markedly altered with PrP deficiency, whereas 92N-PrP^C^ fulfilled a normal function of WT-PrP^C^. We found no evidence for an excessive synaptic contribution of GluN2B, consistent with the unaltered GluN1, GluN2A, and GluN2B levels in the hippocampus. Upon exposure of slices to the selective GluN2B antagonist Ro-25-6981 (Ro-25), the *Prnp^WT^* and *Prnp^92N^* receptors were sensitive to Ro-25, showing a relative increase in the AMPA/NMDA ratio, whereas *Prnp^–/–^* neurons completely lacked sensitivity to Ro-25 (Figure 6B). Additionally, the *Prnp^92N^* and *Prnp^–/–^* neurons showed an accelerated decay time, suggestive of fewer GluN2B subunits localized to the PSD (Figure 6C). The addition of Ro-25 accelerated the decay time in *Prnp^WT^*, *Prnp^92N^*, and *Prnp^–/–^* neurons. The rapid NMDA current decay time suggests that *Prnp^92N^* induced (a) a structural change in GluN2B that alters current dynamics, (b) an increase in triheteromeric receptors at the synapse, or (c) a shift of GluN2B containing the NMDAR to extrasynaptic sites.

### The Prnp^92N^ dendritic beading phenotype is rescued by NMDAR inhibition.

**Figure 7:**
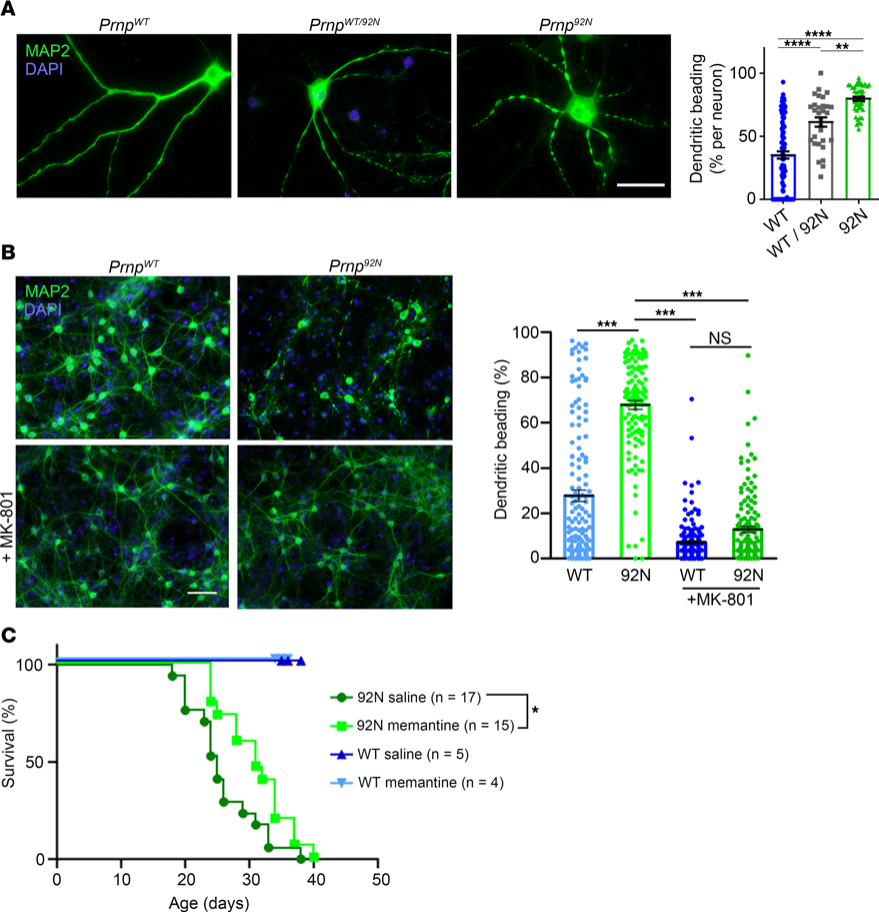
Reversible dendritic beading and prolonged survival of *Prnp^92N^* primary neurons and mice treated with an NMDA antagonist. (**A**) MAP2-labeled hippocampal neurons (DIV 21–28) from *Prnp^92N^* or *Prnp^WT^* littermates. Quantification indicates the mean ± SEM. *n* = 102 (*Prnp^WT^*), 30 (*Prnp^WT/92N^*), and 46 (*Prnp^92N^*) neurons from 3–4 mice per genotype. Scale bar: 100 μm. (**B**) MAP2-labeled cortical neurons (DIV 21–28) treated with vehicle (top) or the NMDAR antagonist MK-801 for 72 hours (bottom). Quantification indicates the average ± SEM. *n* = 145 (*Prnp^WT^*), 117 (*Prnp^92N^*), 165 (MK801-treated *Prnp^WT^*), and 138 (MK801-treated *Prnp^92N^*) neurons from 5 mice per genotype. Scale bar: 200 μm. (**C**) Survival curves for mice treated with memantine or vehicle (saline). *n* = 17 and 15 *Prnp^92N^* saline-treated and memantine-treated mice, respectively; *n* = 5 and 4 *Prnp^WT^* saline-treated and memantine-treated mice, respectively. *P < 0.05, **P < 0.01, ***P < 0.001, and ****P < 0.0001, by 1-way ANOVA with Tukey’s multiple-comparison test (**A** and **B**). A log-rank test was used to determine survival results of *Prnp^92N^* memantine-treated versus saline control in **C**.

We then evaluated primary hippocampal neurons from *Prnp^92N^*, *Prnp^92N/WT^*, and *Prnp^WT^* littermates for phenotypic changes. MAP2-labeled dendrites in *Prnp^92N^* neurons (21 DIV) showed a striking and extensive beading phenotype (Figure 7A), suggestive of excitotoxic or ischemic stress, while *Prnp^92N/WT^* neurons showed moderate dendritic beading, indicating that 1 WT allele was sufficient for a partial rescue.

Similar to hippocampal neurons, *Prnp^92N^* cortical neurons displayed dendritic beading. To test how Ca^2+^ flux through the NMDAR affected the beading phenotype, cultured cortical neurons isolated from *Prnp^92N^* or *Prnp^WT^* littermates were treated with the noncompetitive NMDAR antagonist MK-801 (10 μM) or a vehicle control for 72 hours (Figure 7B). Strikingly, MK801 treatment fully rescued the neuronal phenotype, as the MK801-treated *Prnp^92N^* and *Prnp^WT^* neurons were indistinguishable, suggesting that the marked dendritic beading in the *Prnp^92N^* neurons was due to Ca^2+^ flux through the NMDAR channel.

### An NMDAR antagonist prolongs the survival of Prnp^92N^ mice.

Finally, to determine whether PrP^C^-NMDAR interactions are causal in the excitotoxicity, *Prnp^92N^* and *Prnp^WT^* mice were treated with the noncompetitive NMDAR antagonist memantine (10 mg / kg) or vehicle, i.p. twice daily, starting from P7–P8. Memantine selectively blocks open NMDAR channels by binding within a hydrophobic ring in the channel (54) and is used to treat chronic Ca^2+^ influx through the NMDAR (55). Strikingly, we found that the memantine-treated *Prnp^92N^* mice survived significantly (*P* = 0.01) longer than the control mice (Figure 7C), suggesting heightened NMDAR channel activity and chronically elevated Ca^2+^ as the mechanism underlying the excitotoxicity.

Collectively, these in vitro and in vivo data support a model of PrP^C^ as an important contributor to the regulation of NMDAR channel activity and PKC activity. Dysregulated PrP^C^-NMDAR interactions, through a genetic mutation in the N-terminus of *Prnp*, or potentially by PrP^Sc^ binding, may induce heightened NMDAR channel activity, tonically open Ca^2+^ channels, and phosphorylation of GluN2B at a PKC or CaMKII site, potentiating Ca^2+^ influx in a chronic feed-forward mechanism and inducing neuronal necrosis via runaway excitotoxicity. Given that mouse models of AD and prion-infected mice also show evidence of elevated neuronal activity (56) and dysregulated Ca^2+^-dependent kinase signaling (57–59), including PKC as shown here, these data suggest that other protein oligomers may also dysregulate the PrP^C^/NMDAR/PKC axis and drive excitotoxicity.

## Discussion

The balance between excitatory (E) and inhibitory (I) neurons is dynamic and critical for tuning neuronal circuit activity while avoiding pathological hyperexcitability (60, 61). Here, we provide evidence that PrP^C^ functions at the postsynapse to regulate Ca^2+^ influx through NMDAR channels, affecting PKC activity, which further tuned the NMDAR current via GluN2B phosphorylation. Specifically, we found that disrupting the PrP^C^ N-terminal structure through introduction of a glycan caused elevated PKC activity, NMDAR phosphorylation at GluN2B-S1303, seizures, and rapidly progressive, fatal excitotoxicity. Blocking NMDAR channels reversed dendritic beading in vitro and prolonged the survival of *Prnp^92N^* mice. Given that pathologic Aβ and α-Syn oligomers also bind PrP^C^ (7, 8, 10, 12) and induce GluN2B phosphorylation (8, 11, 12, 62), these findings raise the possibility that perturbing PrP^C^ interactions with the NMDAR or other receptors at the postsynapse may have a broader role in disrupting the E/I balance in neurodegenerative disorders.

Phosphoproteomics was used for the first time to our knowledge to understand PrP^C^-linked signaling and revealed disrupted Ca^2+^ signaling pathways in the *Prnp^92N^* brain, including elevated PKC activity. Ca^2+^, whether derived from external sources or internal stores, activates conventional PKC isozymes (63). Remarkably, exclusively neuronal 92N-PrP^C^ expression was sufficient to heighten PKC activity. Here, we also found evidence of increased PKC activity in bonafide prion disease. These findings revealed PKC as a potentially important driver of PrP^C^-associated disease, in addition to AD (57) and spinocerebellar ataxia (64). A role for PKC in positive modulation of the NMDAR has been recognized for decades (65). Specifically, PKC directly phosphorylates GluN2B at S1303 and thereby acts as an intracellular control for GluN2B/GluN1 receptor currents (44), increasing NMDAR localization to the cell surface as well as the NMDAR opening rate, trafficking, and gating (66, 67), which potentiate Ca^2+^ influx and Ca^2+^-driven kinase activity (44, 66).

Astrocytes highly express PrP^C^ and the NMDAR and are markedly reactive in *Prnp^92N^* mice and in prion infection, yet the trigger of the astrocyte response is unclear. Remarkably, mice with 92N-PrP^C^ expressed exclusively in astrocytes showed no phenotype, whereas neuron-specific expression led to increased neuronal activity (indicated by Npas4), PKC activity, reactive astrocytes, and rapid lethality, suggesting neuronal PrP^C^-mediated signaling as causal for excitotoxicity and the massive glial response. We found reduced EAAT2 levels in mice expressing neuronal 92N-PrP^C^, indicating that neither aggregates nor astrocyte-specific PrP^C^ were required for astrocyte dysfunction and that hyperactive neurons may underlie the reduced EAAT2 levels. Low EAAT2 levels would be expected to hamper glutamate clearance in the synaptic cleft and further exacerbate excitotoxicity.

Similar to the *Prnp^92N^* model, prion-infected mice show evidence of increased neuronal activity, with high levels of Arc, markedly enlarged spines, and elevated pGluA1 (S845) levels in the cortex (68, 69), as well as dendritic varicosities (39, 40). We found that PKC substrates and pGluN2B (S1303) levels were also increased in prion-infected mice, suggesting that prion aggregates may similarly drive a chronic, intensifying neuronal hyperactivity and E/I imbalance. A mouse model of Creutzfeldt-Jakob disease (CJD) also showed heightened susceptibility to focal hippocampal seizures (70).

In patients with CJD, clinical signs are also consistent with cortical hyperexcitability (71) and can include myoclonus (72), seizures (73), and hyperekplexia (exaggerated startle response) (74), as well as periodic sharp wave complexes on EEG (75), some of which overlap with anti-NMDAR encephalitis (76). Human brain organoids and induced pluripotent stem cell–derived (iPSC-derived) neurons harboring human *PRNP* mutations also show disrupted neuronal activity (77) and poor colocalization of the NMDAR and PSD95 (78), respectively. Further studies are needed to understand the relationship between neuronal activity, PKC, and prion disease progression.

PrP^Sc^ has been proposed to induce neuronal toxicity by (a) inducing neuroinflammation (79); (b) perturbing the extracellular matrix (80); (c) disrupting the phospholipid bilayer and inducing ionic currents (20, 81); (d) activating the unfolded protein response (82, 83); or (e) unleashing an intracellular signaling cascade (9, 83). Our data support a signaling cascade initiated by PrP^C^ and the NMDAR that drives runaway excitotoxicity. Whether PrP^Sc^ also dysregulates NMDAR current is unknown, however a report that memantine partially rescues the disease phenotype in prion-infected mice (84) indicates a common mechanism of excitotoxicity involving Ca^2+^ influx through the NMDAR. Nevertheless, the 92N mutation may not mimic all aspects of infectious prion models.

Similar to prion-infected mice (73, 85, 86), *Prnp^92N^* mice develop seizures, hippocampal neuronal toxicity, vacuolation, and hippocampal (CA1) dendrite and synaptic degeneration. The transgenic mouse models reported by Shmerling et al. and others that express an N-terminal internal deletion develop a different phenotype, characterized by ataxia together with cerebellar granule cell degeneration and white matter pathology, which is rescued by the coexpression of WT PrP^C^ (21, 22, 87). Our *Prnp^92N^* model builds on important findings revealed from these noninfectious N-terminal mutation models, however, differs in being a knockin mouse expressing full-length PrP^C^ with 1 residue substitution (more than 99% of sequence conserved) that more closely resembles the clinical and pathologic phenotype of infectious prion disease models and is suggestive of heightened neuronal activity.

Given the high PrP^C^ expression at the synapse (88), together with the powerful signaling response unleashed in glutamatergic neurons, our results indicate that PrP^C^ is a potential pivotal regulator of Ca^2+^ influx through the NMDAR. How PrP^C^ regulates the NMDAR and potentially other receptors at the PSD as well as the full downstream signaling cascade remains to be seen, yet may have implications for other diseases of E/I imbalance, including stroke, epilepsy, and neurodegenerative disease. These data also underscore how signaling in neurons promotes rapidly progressive neurodegenerative disease and highlight the importance of advancing therapies to thwart NMDAR-driven pathogenic signaling cascades.

## Methods

Additional details on methods are provided in Supplemental Methods.

### Sex as a biological variable.

Both male and female animals were used in this study. Sex was not considered as a biological variable, as both males and females were similarly affected with neurodegenerative disease.

### Knockin Prnp^92N^ and Prnp^92Q^ mouse generation using the CRISPR/Cas9 system.

Knockin mice were generated by the UC Irvine Mouse Genetics core by microinjection of Cas9 ribonucleoprotein (PNA Bio) into B6SJLF1 × C57BL/6NJ zygotes. Briefly, Cas9 (20 ng/μL), gRNA-1 (20 ng/μL), and ssDNA HDR template (10 ng/μL) were mixed in injection buffer (10 mM Tris, 0.1  mM EDTA) and incubated on ice for 10 minutes, as per the manufacturer’s instructions. The Cas9 mixture was microinjected into the pronucleus of single-cell zygotes isolated from superovulated females. All founders and select progeny were genotyped by Sanger sequencing of genomic DNA from proteinase K digested tail tissue. Thirteen founder mice carried the mutated *Prnp^92N^* allele, and 10 founders carried the *Prnp^92Q^* allele. Select lines were backcrossed with C57BL/6J mice.

*Prnp^92N^* heterozygous mice were interbred to generate *Prnp^92N/92N^*, *Prnp^92N/WT^*, and *Prnp^WT/WT^* littermate controls and were also bred with *Prnp^–/–^* (*ZH3*) mice (35) to generate *Prnp^92N/–^* mice. *Prnp^–/–^* (*ZH3*) mice were a gift from Adriano Aguzzi, University of Zurich (Zurich, Switzerland). *Prnp^92Q^* heterozygous mice were initially interbred to generate *Prnp^92Q/92Q^* mice, which were subsequently bred as a homozygous line.

All animals were maintained under pathogen-free conditions on a 12-hour light/12-hour dark cycle. Mice had access to standard laboratory chow and water ad libitum.

### Genotyping and zygosity.

Genotyping and zygosity were determined from PK digested tissue samples by quantitative PCR using primers for *Prnp* (forward: TGGGGACAACCTCATGGTGGT; reverse: TGCCACATGCTTGAGGTTGGTT); a mutant *Prnp^92N^* probe (TGGATGGGGCCAAGGAAACGGTACCC) (Hex555/ZEN/Iowa Block FQ); and a *Prnp^WT^* probe (TGGATGGGGCCAAGGAGGGGGT) (FAM520/Black Hole Quencher1) together with Kapa Probe Fast Universal Master Mix (Kapa Biosystems, KK4701). Samples were analyzed on a Bio-Rad CFX 96 using Bio-Rad CFX Manager 3.1 software. Reaction conditions were 10 μL reaction volume with an initial enzyme activation at 95°C for 3 minutes, followed by 39 cycles of 95°C for 3 seconds and 60°C for 20 seconds.

### Mouse clinical characterization and brain collection.

Starting at P15, mice were monitored daily for the development of neurological signs, including ataxia, circling, stiff (straub) tail, hind leg clasp, hind leg paraparesis or paraplegia, behavior arrest, tremors, neck jerks, hyperactivity, opisthotonos, seizures, and nonspecific signs of disease (for example, abnormal mentation, anorexia or hyporexia, slow movement, kyphosis, weight loss, rough coat, blepharospasm, or ocular discharge). Select cohorts were monitored for body weight. Mice were euthanized at the onset of terminal disease, determined as severe kyphosis, ataxia, immobility, rapid weight loss, and/or status epilepticus. The brain was halved along the longitudinal fissure, and 1 hemisphere was immediately fixed in formalin. The contralateral hippocampus and cerebral cortex were snap-frozen in liquid nitrogen.

For prion infections, *Prnp^WT^* mice were anesthetized with ketamine and xylazine, inoculated into the left parietal cortex with 30 μL 1% prion-infected brain homogenate prepared from terminally ill mice (strain ME7) and euthanized at the onset of terminal prion disease.

### Study approval.

All animal studies were performed following procedures to minimize suffering and were approved by the IACUC at UCSD. Protocols were performed in strict accordance with good animal practices, as described in the NIH’s *Guide for the Use and Care of Laboratory Animals* (National Academies Press, 2011).

### Statistics.

Animals were randomly assigned to groups, with consideration of maintaining a similar male/female balance. Pooled neurons were randomly divided and plated for the treatment and control groups. A 1-way or 2-way ANOVA with Tukey’s multiple-comparison post hoc test was used to compare normally distributed continuous data (PrP^C^ expression and glycoforms, excitatory postsynaptic currents, soluble and insoluble PrP in brain lysates from uninfected and prion-infected *Prnp^WT^* mice, in vitro cortical and hippocampal neuronal beading, flotation assay). A 2-way ANOVA with Šidák’s multiple-comparisons test was used to compare the weights of Prnp WT and *Prnp^92N^* mice. A log-rank (Mantel-Cox) test was used to assess the survival differences between AAV-transduced mice and memantine-treated and vehicle-treated mice. The means of 2 groups were compared using an unpaired, 2-tailed *t* test (Fura-2 experiments [cytosolic Ca^2+^ concentrations, the AUC, and the 340:380 ratio] and RK13 cells expressing mutant or WT *Prnp*), with Welch’s correction added when group sizes varied (protein levels by Western blotting, IHC, and immunofluorescence staining). Data (summary) are shown as the mean ± SEM. For mass spectrometry data, a protein or peptide was considered significantly different if it had an absolute log_2_(fold change) greater than log_2_(1.2) and an unadjusted *P* value less than 0.05 as assessed by Welch’s t test. Statistical analysis was performed using GraphPad Prism 10 (GraphPad Software). For all analyses, A *P* value of 0.05 or less was considered significant.

### Data availability.

The mass spectrometry analysis results may be found in the Supplemental tables. The mass spectrometry raw files are available on ProteomeXchange (accession code PXD051060). Values for all data points in graphs are reported in Supporting Data Values file. Contact the corresponding author for other original data or detailed protocols.

## Author contributions

CJS and TDK conceptualized and designed the CRISPR-knockin mice. JL, JAC, JEM, DOJ, TDK, SAL, JDB, GNP, KD, and JRY contributed to the experimental design. JL, JAC, DOJ, MP, TDK, GAD, HK, KS, JER, DPP, YD, MAG, AMS, JW, CDO, JC, GF, PAC, JDB, and BDA performed experiments and analyzed the data. DBM performed all mass spectrometry, and JEM analyzed the mass spectrometry data. SR, JMR, ACN, and BC contributed to method design and data analysis and interpretation. The manuscript was written by CJS, with input from all authors. The order of the 3 first authors’ names was determined by joint decision of the authors.

## Supplementary Material

Supplemental data

Unedited blot and gel images

Supplemental table 1

Supplemental table 2

Supplemental video 1

Supplemental video 2

Supplemental video 3

Supplemental video 4

Supplemental video 5

Supporting data values

## Version 1 04/04/2025

In-Press Preview

## Version 2 05/15/2025

Electronic publication
