# Supplementary material for: Mutant prion protein enhances NMDA receptor activity, activates PKC, and triggers rapid excitotoxicity in mice
Source: J Clin Invest. 2025 Apr 4;135(10):e186432. doi: 10.1172/JCI186432 (PMC12077891; doi:10.1172/JCI186432)
Supplement: Supplemental data [file jci-135-186432-s206.pdf]

Supplemental Figures

Mutant prion protein enhances NMDA receptor activity, activates PKC, and triggers rapid excitotoxicity in mice

Joie Lin[1*], Julia A. Callender[1*], Joshua E. Mayfield[1*], Daniel B. McClatchy[2], Daniel Ojeda-Juárez[1], Mahsa Pourhamzeh[1], Katrin Soldau[1], Timothy D. Kurt[1], Garrett A. Danque[1], Helen Khuu[1], Josephina E. Ronson[1], Donald Pizzo[1], Yixing Du[3], Maxwell A. Gruber[1], Alejandro M. Sevillano[1], Jin Wang[1], Christina D. Orrú[4], Joy Chen[1], Gail Funk[1], Patricia Aguilar-Calvo[1], Brent Aulston[1], Subhojit Roy[1,3], Jong M. Rho[3], Jack Bui[1], Alexandra Newton[5], Stuart A. Lipton[6], Byron Caughey[4], Gentry N. Patrick[7], Kim Doré[3], John R. Yates III[2], Christina J. Sigurdson[1,8,9]

[1]Department of Pathology, University of California at San Diego, La Jolla, CA, USA
[2]Department of Molecular Medicine, The Scripps Research Institute, La Jolla, CA, USA
[3]Department of Neurosciences, University of California at San Diego, La Jolla, CA, USA
[4]Laboratory of Persistent Viral Diseases, Rocky Mountain Laboratories, NIAID, NIH, Hamilton, MT, USA
[5]Department of Pharmacology, University of California at San Diego, La Jolla, CA, USA
[6]Department of Molecular Medicine and Neuroscience, Neurodegenerative New Medicines Center, The Scripps Research Institute, La Jolla, CA, USA
[7]Department of Biology, University of California at San Diego, La Jolla, CA, USA
[8]Department of Medicine, University of California at San Diego, La Jolla, CA, USA
[9]Department of Pathology, Immunology, and Microbiology, University of California at Davis, Davis, CA 95616, USA
*Equal contribution

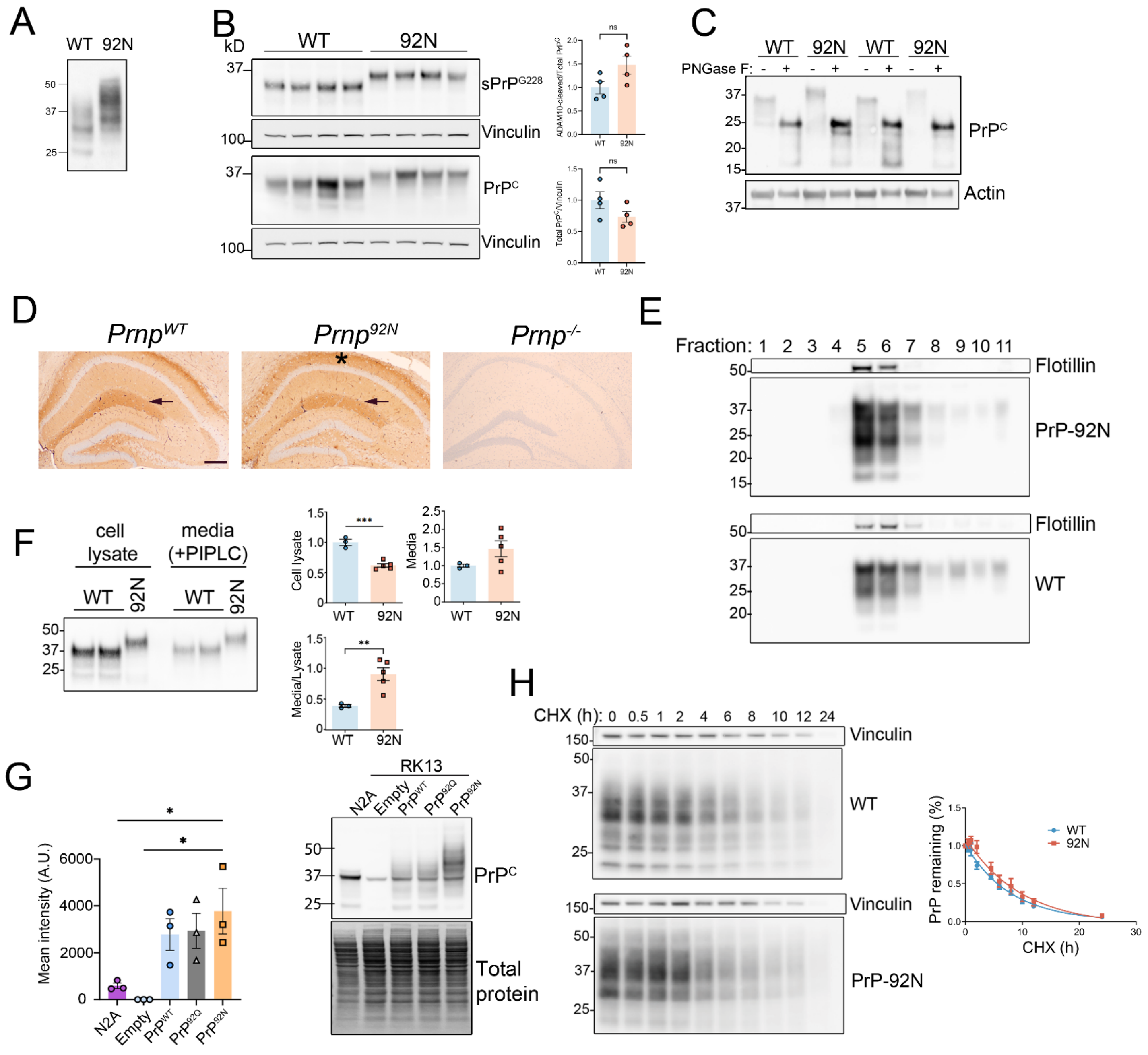

Supplemental Figure 1. Addition of a third glycan to the N-terminal domain of PrP^{C} does not affect localization to the cell surface, distribution in the brain, localization to lipid rafts, or degradation kinetics. (A) PrP^{C} western blot of RK13 cells transfected with WT- or 92N-PrP^{C} encoding plasmids shows mobility shift and the tri-, di-, mono-, and un-glycosylated PrP^{C} bands. (B) Western blot and quantification of ADAM10-cleaved (sPrP^{G228}) and total PrP^{C} in P20 *Prnp*^{WT} or *Prnp*^{92N} cortex. (C) Western blotting for PrP^{C} reveals a single primary band following glycan digestion with PNGase F. (D) PrP^{C} immunostaining in the hippocampus reveals intense PrP^{C} expression in the stratum lacunosum moleculare (arrow) in *Prnp*^{WT} and *Prnp*^{92N} mice (P20). Asterisk denotes where PrP^{C} staining is more intense in the stratum oriens of the *Prnp*^{92N} mouse. *Prnp*$^{-/-}$ mice are shown as a negative control. (E) Western blots of flotillin-1-containing DRM fractions reveal similar localization of WT- and 92N-PrP^{C} and GluN2B to DRMs. (F) Western blots depict PIPLC cleavage of surface PrPC protein from live primary neurons cultured from *Prnp*^{WT} or *Prnp*^{92N} littermates. (G) Surface levels of PrP^{C} detected using flow cytometry of RK13 cells transfected with empty vector, WT-PrP^{C}, 92Q-PrP^{C}, or 92N-PrP^{C}. Neuro2A cells were used as a positive control. (H) Immunoblotting of RK13 and Neuro2A showing PrP^{C} following transfection. (I)

Western blot analysis of cycloheximide chase experiment reveals the half-time for degradation of WT- or 92N-PrPC overexpressed in RK13 cells. For panels B, F and I, graphs show mean ± SEM from at least three separate biological replicates. Unpaired, two-tailed t-test [panels B, F, I (comparing half-life)]. For panel G, graph show mean ± SEM from three separate biological replicates. One-way ANOVA with Tukey multiple comparison test.

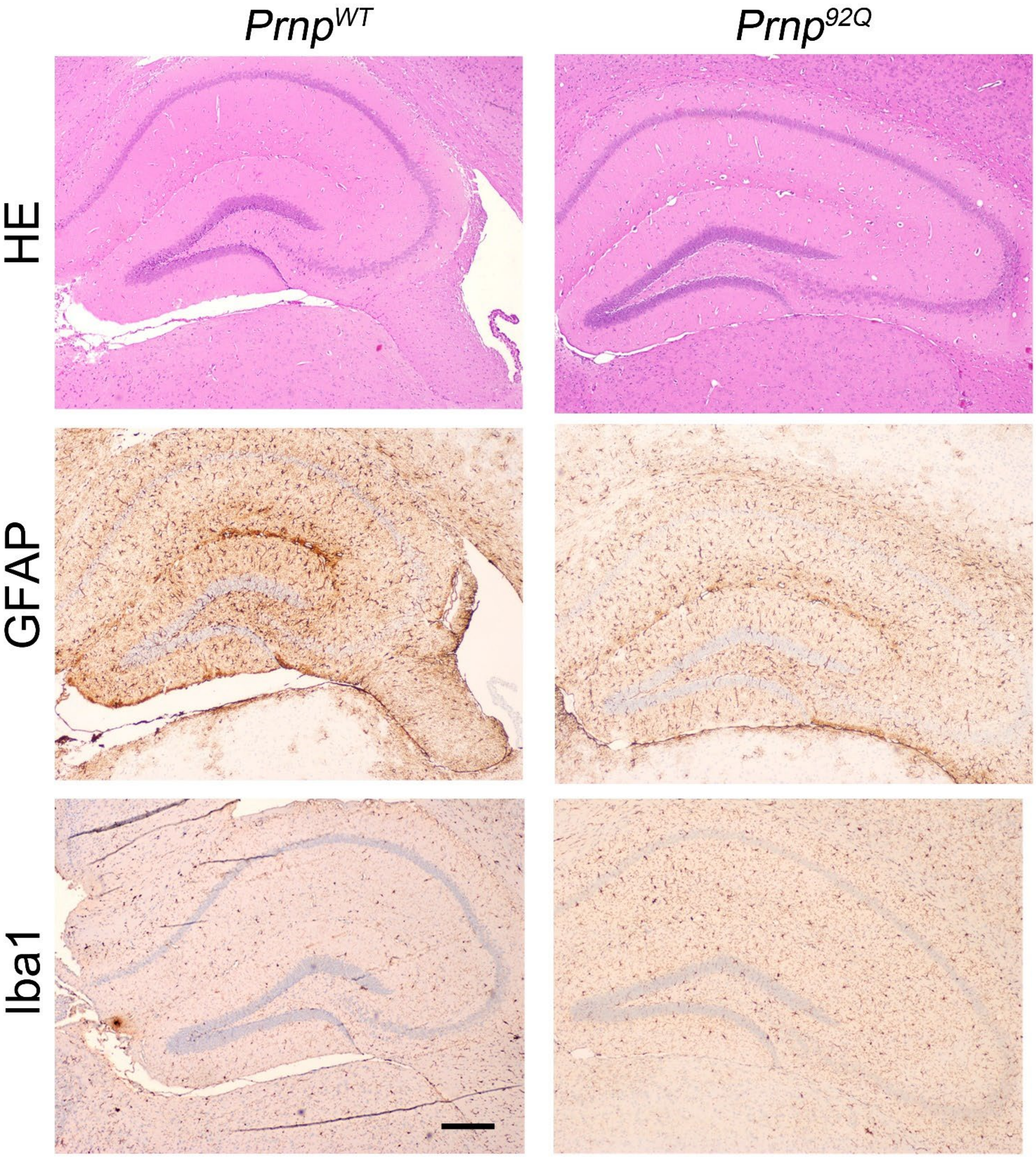

Supplemental Figure 2. Glutamine substitution at position 92 (G92Q) does not induce neurotoxicity. Representative HE stain and GFAP- and Iba1-immunolabelled images of the hippocampus of homozygous *Prnp*^{92Q} knockin mice show no lesions or astrocyte reactivity, similar to the *Prnp*^{WT} control. *Prnp*^{92Q} mouse: 533 days old; *Prnp*^{WT} mouse: 388 days old. Scale bar = 500 mm.

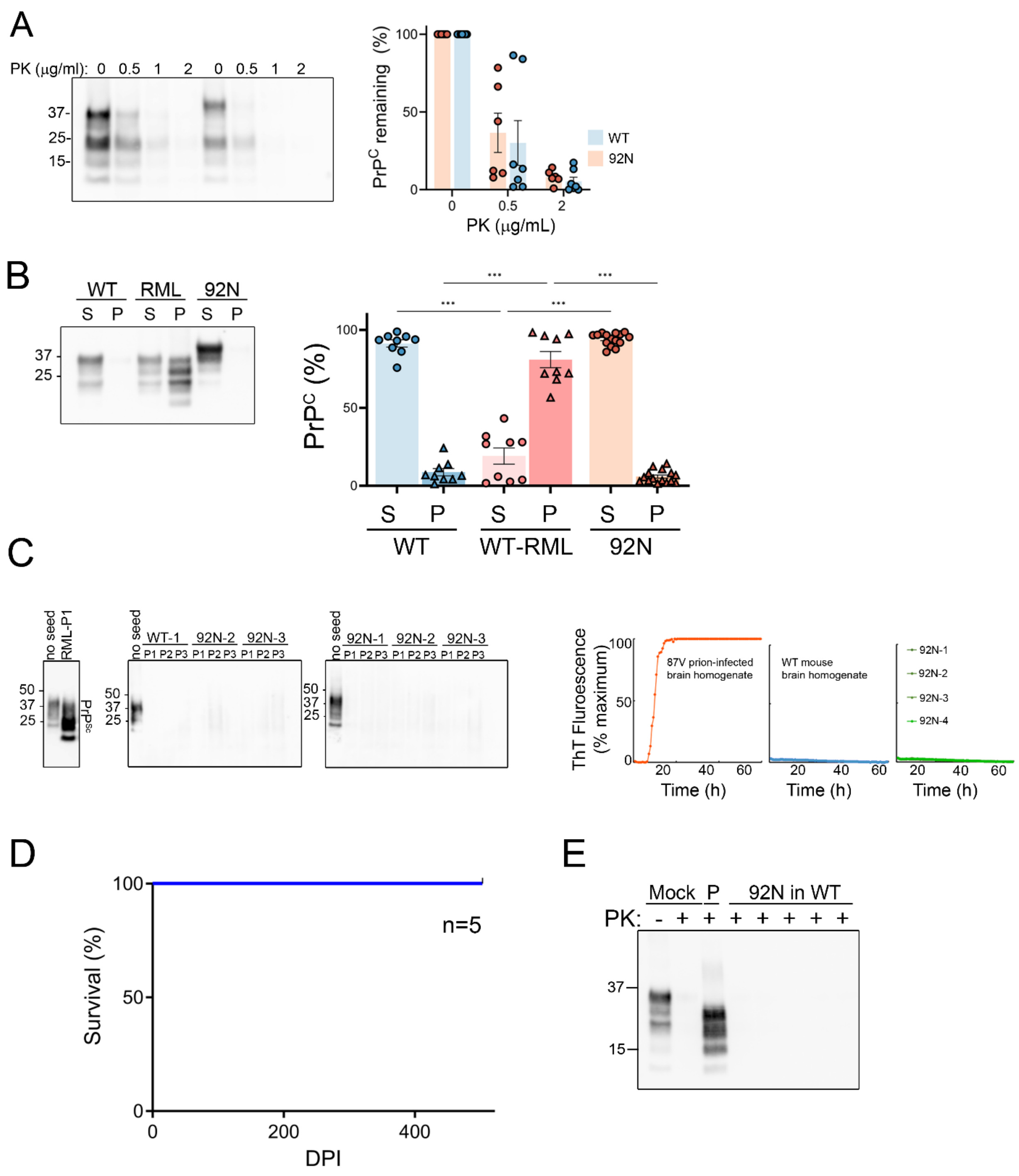

Supplemental Figure 3. 92N-PrP^{C} is highly soluble, sensitive to proteases, and lacks seeding activity and infectivity. (A) Western blot and quantification of proteinase K (PK)-digested brain lysate from *Prnp^{WT}* and *Prnp^{92N}* mice. N = 6 (*Prnp^{WT}*) and n = 7 (*Prnp^{92N}*). (B) Western blot of soluble (S) and insoluble (P) PrP^{C} after high speed centrifugation of brain lysate from *Prnp^{92N}* mice and uninfected and prion-infected *Prnp^{WT}* mice as negative and positive controls for insoluble PrP^{Sc}, respectively. Prion strain was RML. N = 9 (*Prnp^{WT}* and RML) and n = 15 (*Prnp^{92N}*). (C) RT-QuIC analysis of brain lysates from *Prnp^{92N}* or from prion-infected (strain 87V) or -uninfected mice (positive and negative controls) for seeding activity (n = 1 each). (D) Survival curves of *Prnp^{WT}* inoculated intracranially with *Prnp^{92N}* brain (n = 5). (E) Western blots of brains from *Prnp^{WT}* mice inoculated with *Prnp^{92N}* brain homogenate show no detectable PK-resistant PrP^{Sc} (right) (n = 5). Two-way ANOVA with Bonferroni multiple comparisons test. ***P < 0.001.

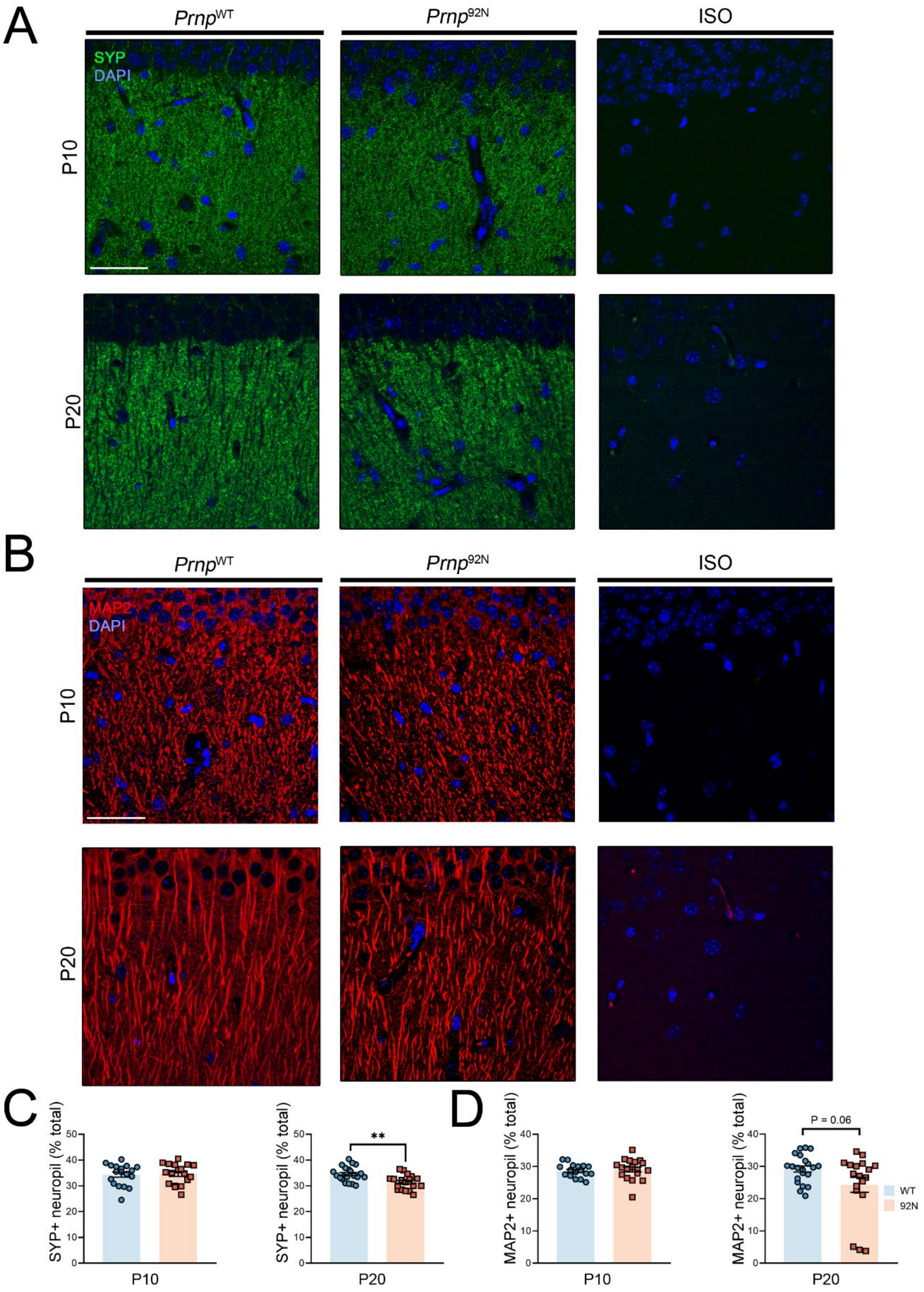

Supplemental Figure 4. Synapse numbers are reduced in *Prnp^{92N}* mice at P20. (A-B) Representative images and quantification of *Prnp^{WT}* or *Prnp^{92N}* hippocampi immunolabelled for synaptophysin (SYP), dendrites (MAP2), and nuclei (DAPI) at P10 and P20. The stratum radiatum was quantified. The P20 isotype control images are from the same field, imaged in different channels. N = 6-7 mice per group, and n = 3 fields per mouse. Scale bar = 50 µm. Bar graphs represent the mean ± SEM. Unpaired, two-tailed t-test with Welch's correction.

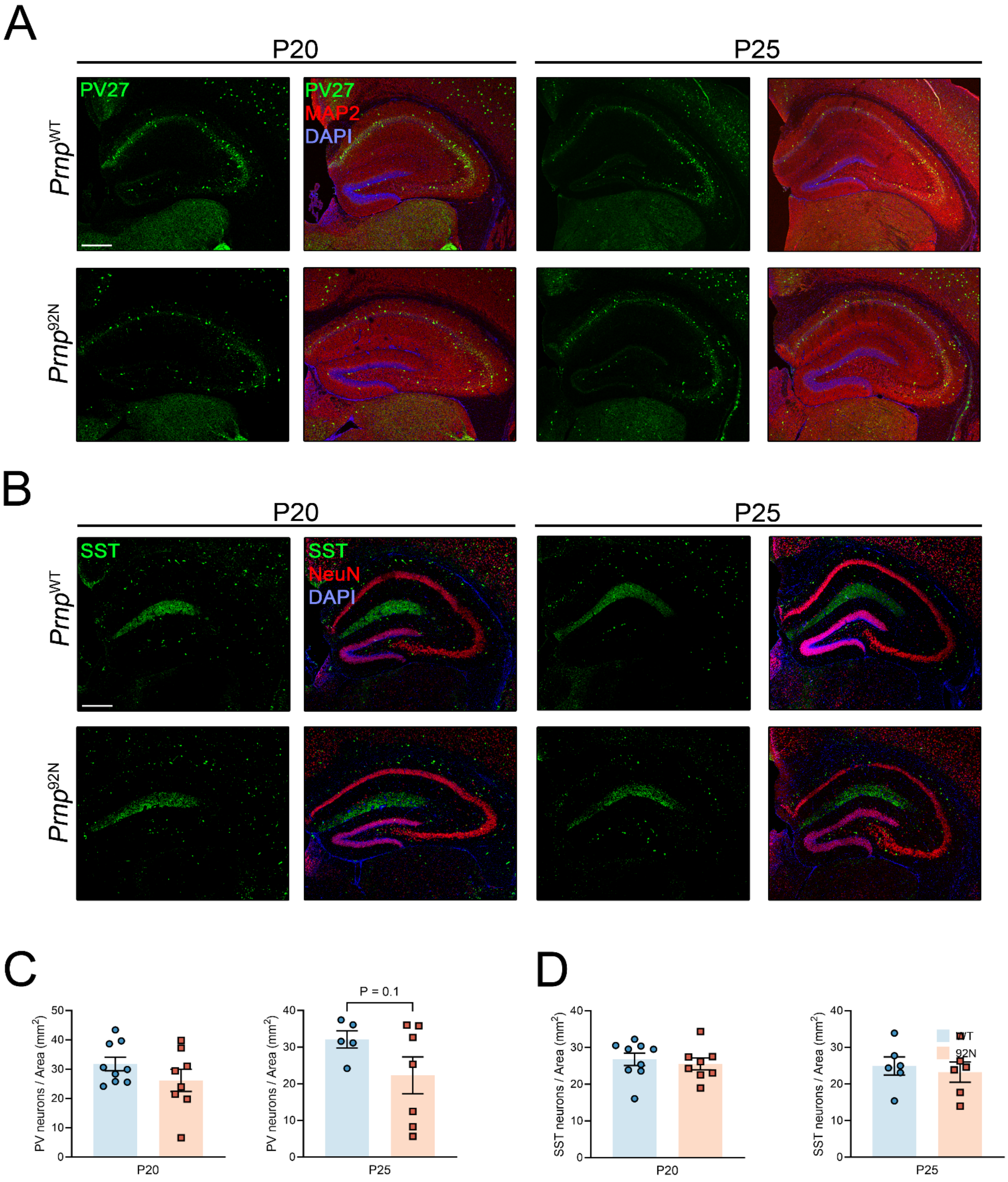

Supplemental Figure 5. Parvalbumin and somatostatin inhibitory neuron numbers are equivalent in *Prnp^WT^* and *Prnp^92N^* hippocampi. (A-B) Representative images and quantification of *Prnp*^{WT} or *Prnp*^{92N} hippocampi immunolabelled for parvalbumin (PV), dendrites (MAP2), and nuclei (DAPI), or (C-D) for somatostatin (SST), NeuN (neuronal nuclei), and DAPI. N = 9 and 8, P20 *Prnp*^{WT} and *Prnp*^{92N} PV and SST, respectively, n = 5 and 7, P25 *Prnp*^{WT} and *Prnp*^{92N} PV, respectively, and n = 6, P25 *Prnp*^{WT} and *Prnp*^{92N} SST. Scale bar = 500 μm. Bar graphs represent the mean ± SEM. Unpaired, two-tailed t-test with Welch's correction.

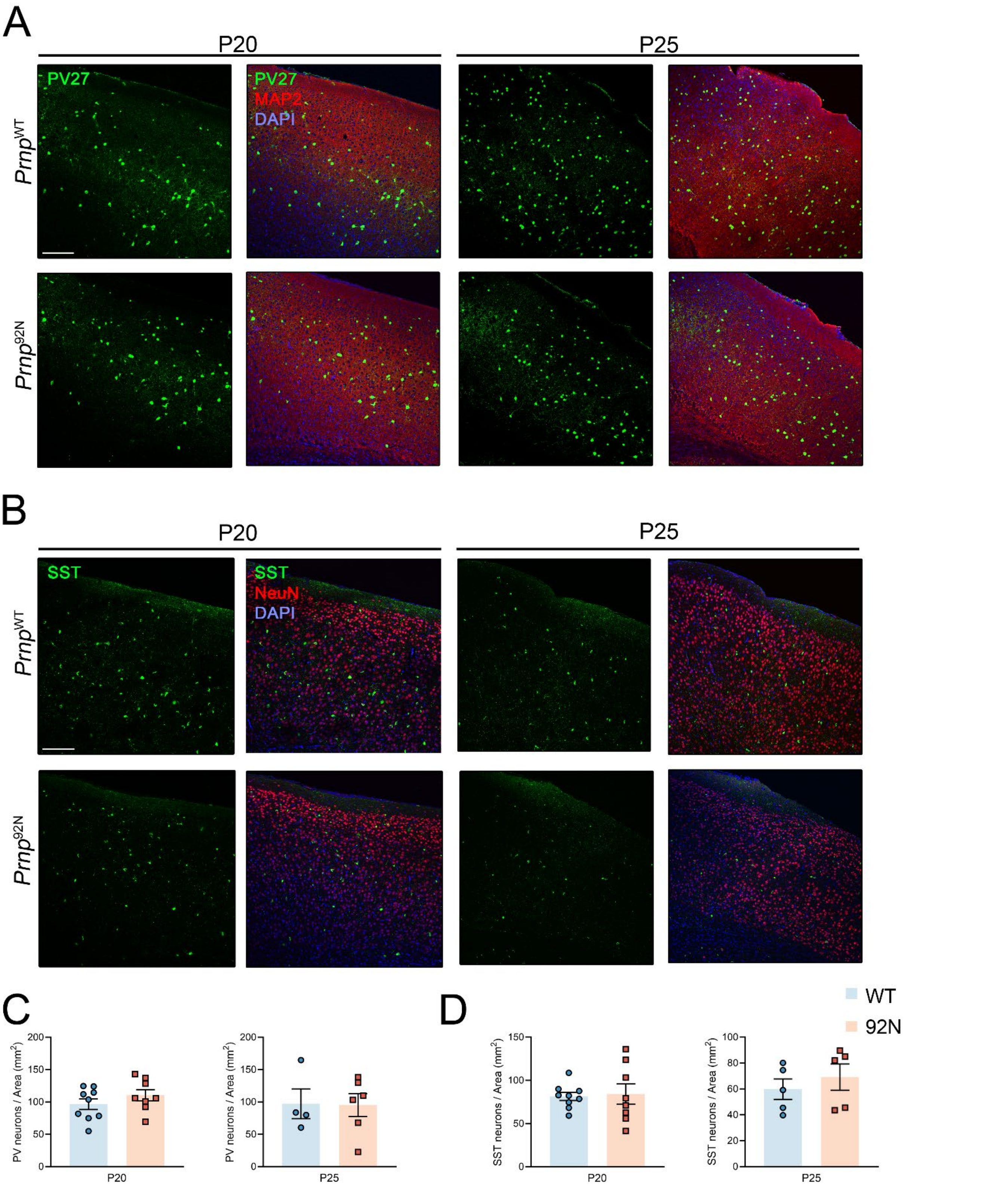

Supplemental Figure 6. Parvalbumin and somatostatin inhibitory neuron numbers are equivalent in *Prnp^WT^* and *Prnp^92N^* motor cortex. (A-B) Representative images and quantification of *Prnp^WT^* or *Prnp^92N^* hippocampi immunolabelled for parvalbumin, dendrites, and nuclei, or (C-D) for somatostatin, neuronal nuclei, and DAPI. N = 9 (P20 *Prnp^WT^* PV and SST), n = 8 (P20 *Prnp^92N^* PV and SST), 4 (P25 *Prnp^WT^* PV), n = 6 (P25 *Prnp^92N^* PV), n = 5 (P25 *Prnp^WT^* SST), and n = 5 (P25 *Prnp^92N^* SST). Scale bar = 200 µm. Bar graphs represent the mean ± SEM. Unpaired, two-tailed t-test with Welch's correction.

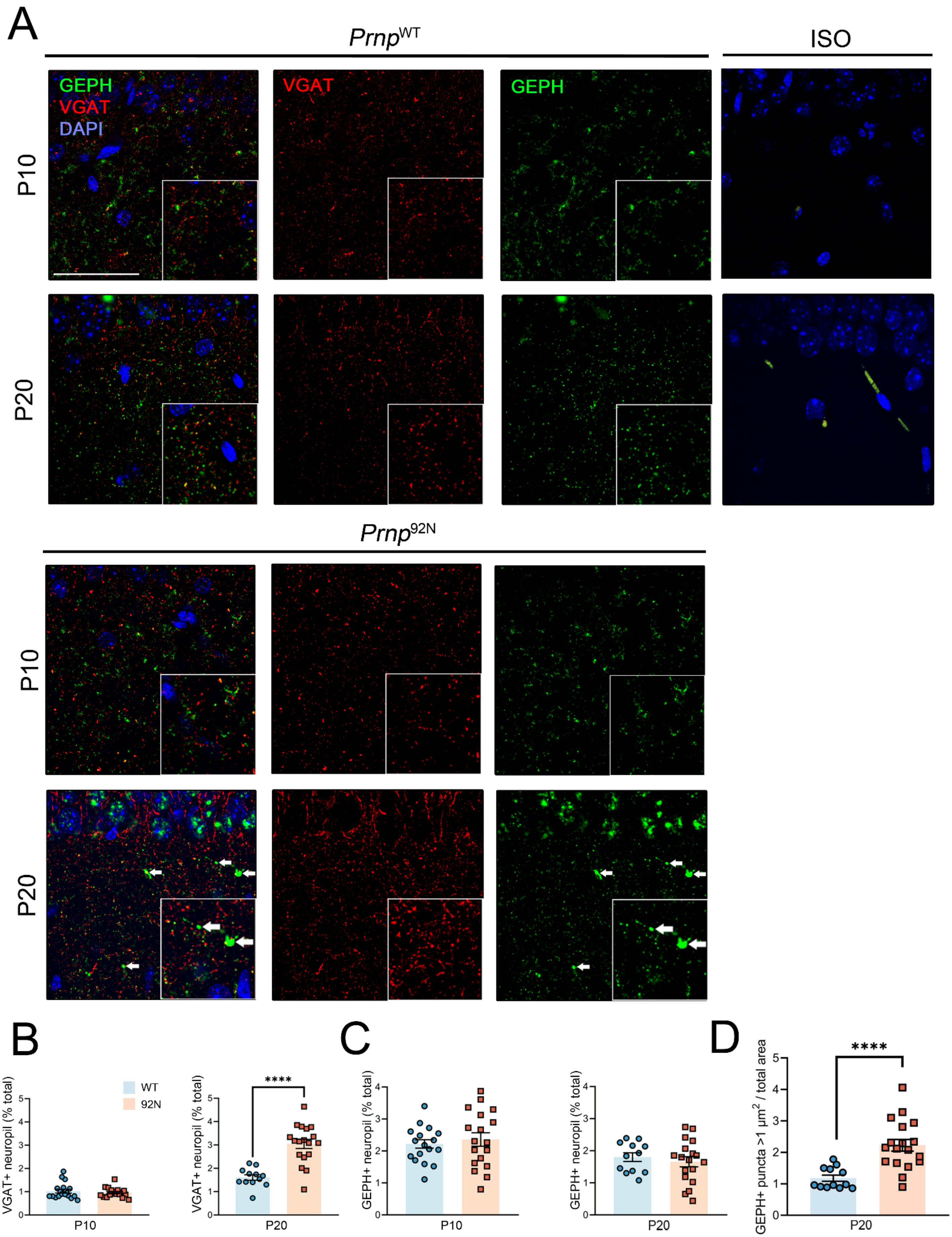

A
Prnp^WT
ISO
GEPH
VGAT
DAPI
VGAT
GEPH
P10
P20
Prnp^92N
P10
P20
B
WT
92N
VGAT+ neuropil (% total)
P10
P20
****
C
GEPH+ neuropil (% total)
P10
P20
D
****
GEPH+ puncta >1 μm² / total area
P20

Supplemental Figure 7. Pre- and post- inhibitory synapses are unaltered at P10, but increased at P20. (A-D) Representative images and quantification of *Prnp*^{WT} or *Prnp*^{92N} hippocampi immunolabelled for VGAT, gephyrin (GEPH), and nuclei (DAPI) at P10 and P20. An isotype control is also shown. Insets show a digital zoom of the VGAT+ synaptic density and GEPH+ post-synapses (white arrows). The stratum radiatum was quantified. N = 4-6 mice per group, n = 3 fields per mouse Scale bar = 50 µm. Bar graphs represent the mean ± SEM. Unpaired, two-tailed t-test with Welch's correction. **** P ≤ 0.0001.

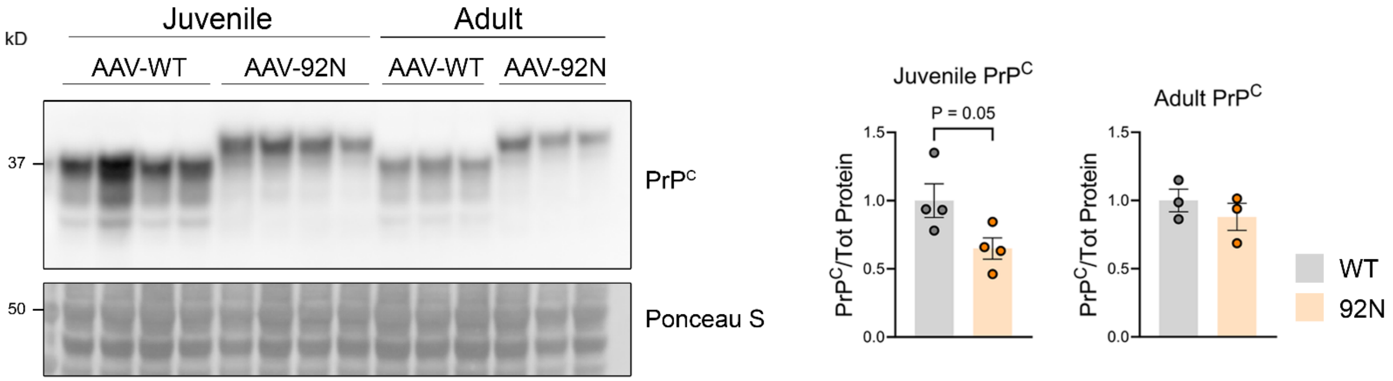

Supplemental Figure 8. PrP^C levels in *Prnp$^{-/-}$* mice transduced with *AAV-hSyn1PrnpWT* or -*hSyn1Prnp92N*. Western blot and quantification of 92N- or WT-PrP^C in hippocampal lysates of juvenile or adult mice (approximately P21 - P22 and P49 - P66 days old). N = 4 per genotype for juveniles and n = 3 per genotype for adults. Unpaired, two-tailed t-test.

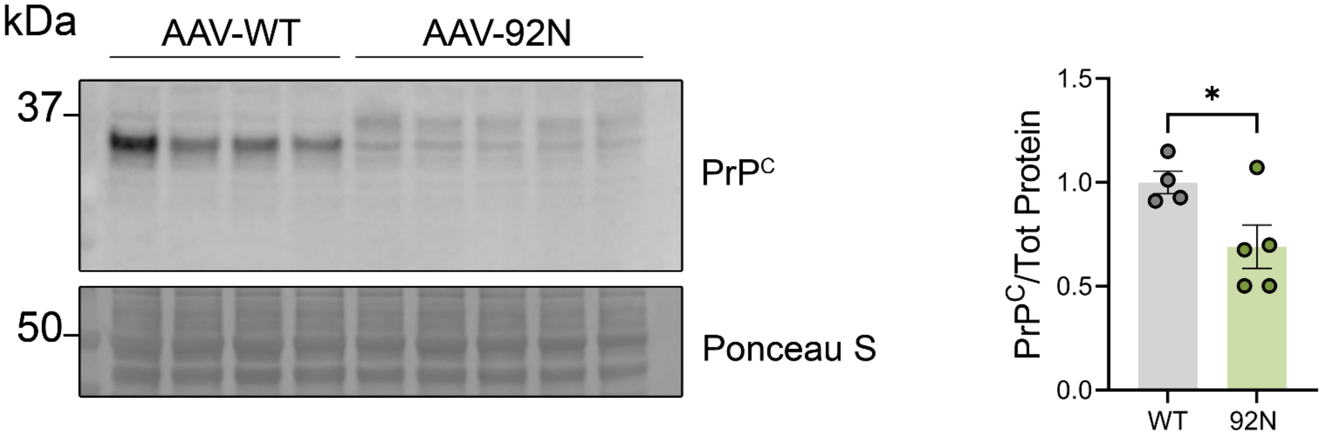

Supplemental Figure 9. PrP^{C} levels in *Prnp$^{-/-}$* mice transduced with *AAV-gfaABC1DPrnp92N* or *-gfaABC1DPrnpWT*. Western blot and quantification of 92N- or WT-PrP^{C} in hippocampal lysates of mice at 16 days post-transduction (injected with AAV at P2). N = 4 for AAV-WT and n = 5 for AAV-92N. Unpaired, two - tailed t-test with Welch's correction. *P < 0.05.

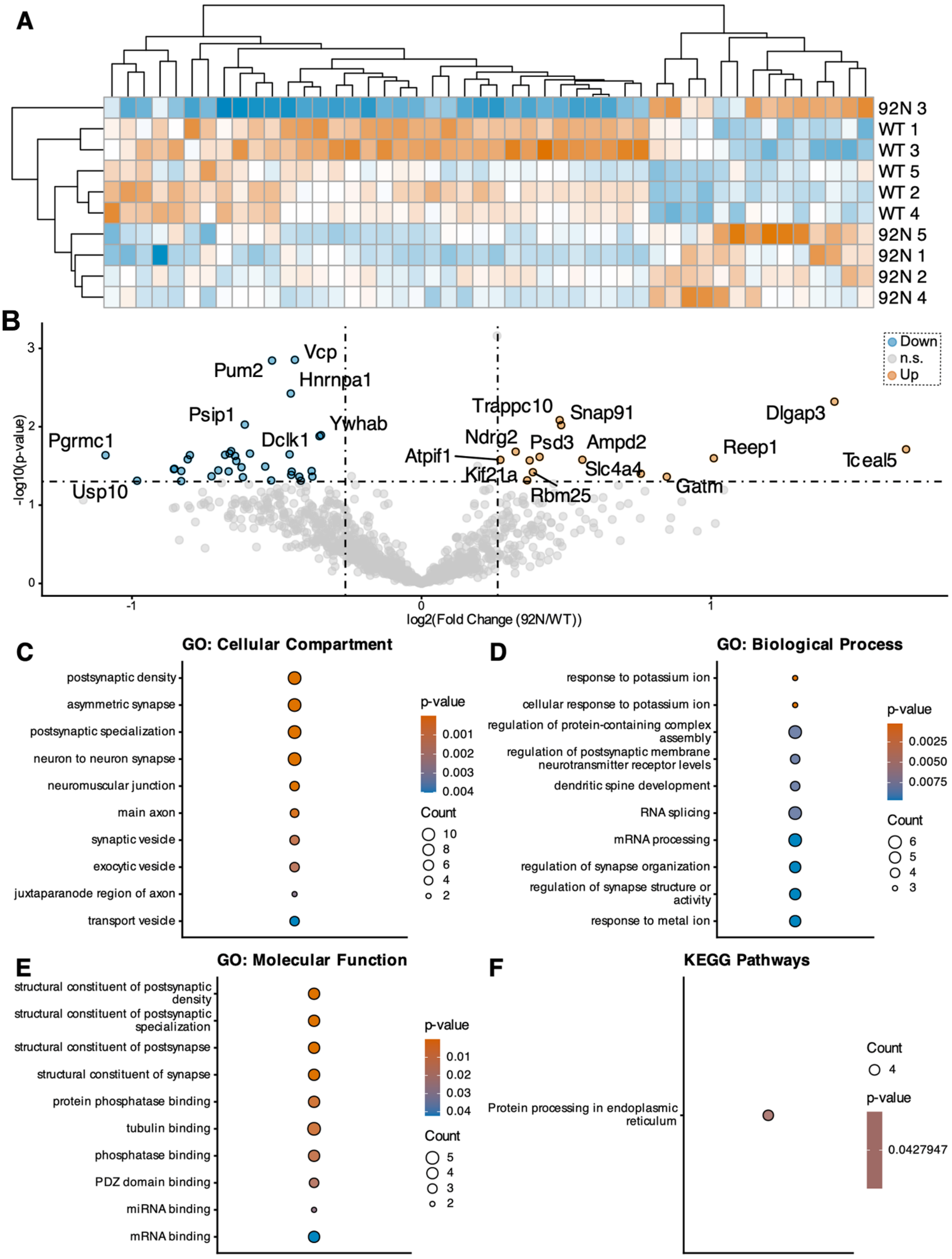

A
92N 3
WT 1
WT 3
WT 5
WT 2
WT 4
92N 5
92N 1
92N 2
92N 4
B
Down
n.s.
Up
Vcp
Pum2
Hnrnpa1
Psip1
Ywhab
Pgrmc1
Dclk1
Usp10
Trappc10
Snap91
Dlgap3
Ndrg2
Psd3
Ampd2
Reep1
Atpif1
Tceal5
Slc4a4
Kif21a
Gatm
Rbm25
-log10(p-value)
log2(Fold Change (92N/WT))
C
GO: Cellular Compartment
postsynaptic density
asymmetric synapse
postsynaptic specialization
neuron to neuron synapse
neuromuscular junction
main axon
synaptic vesicle
exocytic vesicle
juxtaparanode region of axon
transport vesicle
p-value
Count
D
GO: Biological Process
response to potassium ion
cellular response to potassium ion
regulation of protein-containing complex assembly
regulation of postsynaptic membrane neurotransmitter receptor levels
dendritic spine development
RNA splicing
mRNA processing
regulation of synapse organization
regulation of synapse structure or activity
response to metal ion
p-value
Count
E
GO: Molecular Function
structural constituent of postsynaptic density
structural constituent of postsynaptic specialization
structural constituent of postsynapse
structural constituent of synapse
protein phosphatase binding
tubulin binding
phosphatase binding
PDZ domain binding
miRNA binding
mRNA binding
p-value
Count
F
KEGG Pathways
Count
Protein processing in endoplasmic reticulum
p-value
0.0427947

Supplemental Figure 10. ***Prnp**^{92N}* **mutation alters the whole brain phosphoproteome.** (A) Clustered heatmap and volcano plot depict significantly altered phosphopeptides in whole brain lysates from *Prnp*^{WT} or *Prnp*^{92N} mice (P20) analyzed by TMT LC-MS/MS (n = 5 per genotype). Dashed lines indicate fold change and p-value cut-offs. Significantly altered peptides indicated with protein gene symbol. (C-E) Top ten most enriched "Cellular Components", "Biological Process", and "Molecular Function" gene ontology terms based on significantly altered phosphopeptides. Dot fill indicates significance of GO term and dot size indicates gene count. (F) Top ten KEGG pathways for proteins displaying differentially abundant phosphopeptides.

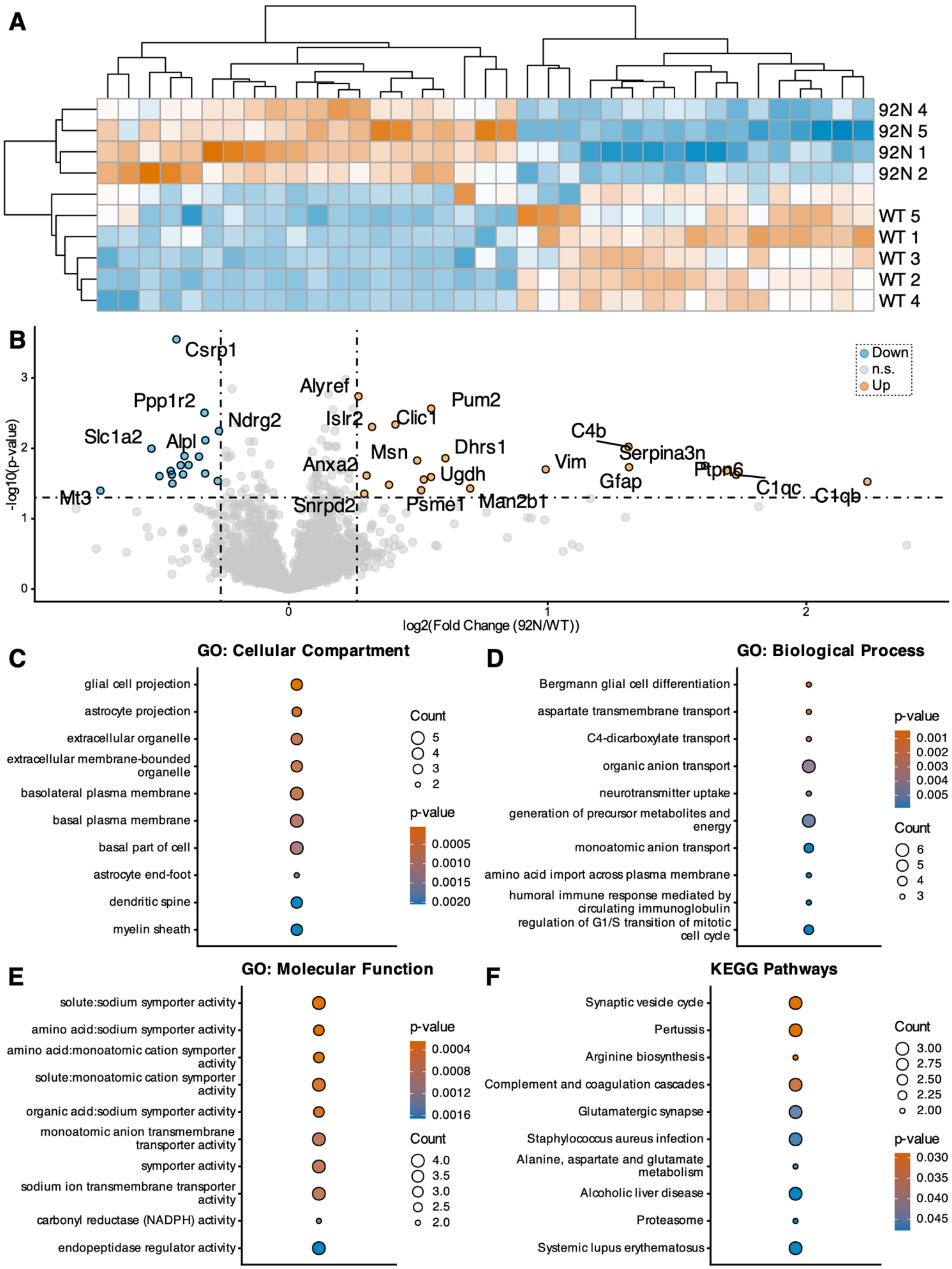

Supplemental Figure 11. *Prnp*^{92} mutation alters the whole brain proteome. (A) Clustered heatmap and volcano plot depict significantly altered peptides in whole brain lysates from *Prnp*^{WT} or *Prnp*^{92N} mice (P20)

analyzed by TMT LC-MS/MS. Dashed lines indicate fold change and p-value cut-offs. Select altered peptides indicated with protein gene symbol. (C-E) Top ten most enriched “Cellular Components”, “Biological Process”, and “Molecular Function” gene ontology terms based on significantly altered peptides. Dot fill indicates significance of GO term and dot size indicates gene count. (F) Top ten KEGG pathways for proteins displaying differentially abundant peptides.

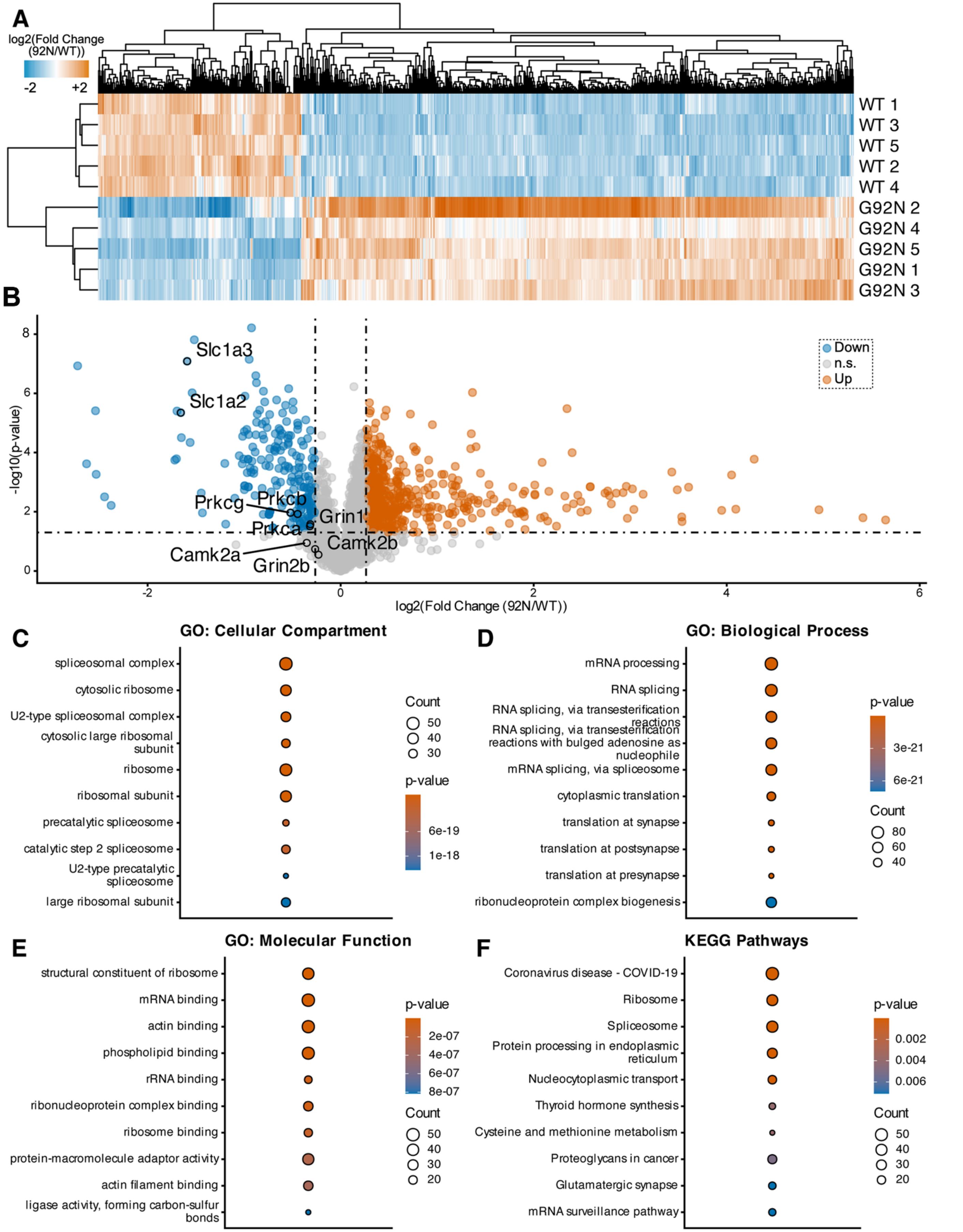

Supplemental Figure 12. *Prnp*^{92} mutation alters the hippocampal proteome. (A) Clustered heatmap and volcano plot depict significantly altered peptides in hippocampal lysates from *Prnp*^{WT} or *Prnp*^{92N} mice (P20)

analyzed by TMT LC-MS/MS. Dashed lines indicate fold change and p-value cut-offs. Select altered peptides indicated with protein gene symbol. (C-E) Top ten most enriched “Cellular Components”, “Biological Process”, and “Molecular Function” gene ontology terms based on significantly altered peptides. Dot fill indicates significance of GO term and dot size indicates gene count. (F) Top ten KEGG pathways for proteins displaying differentially abundant peptides.

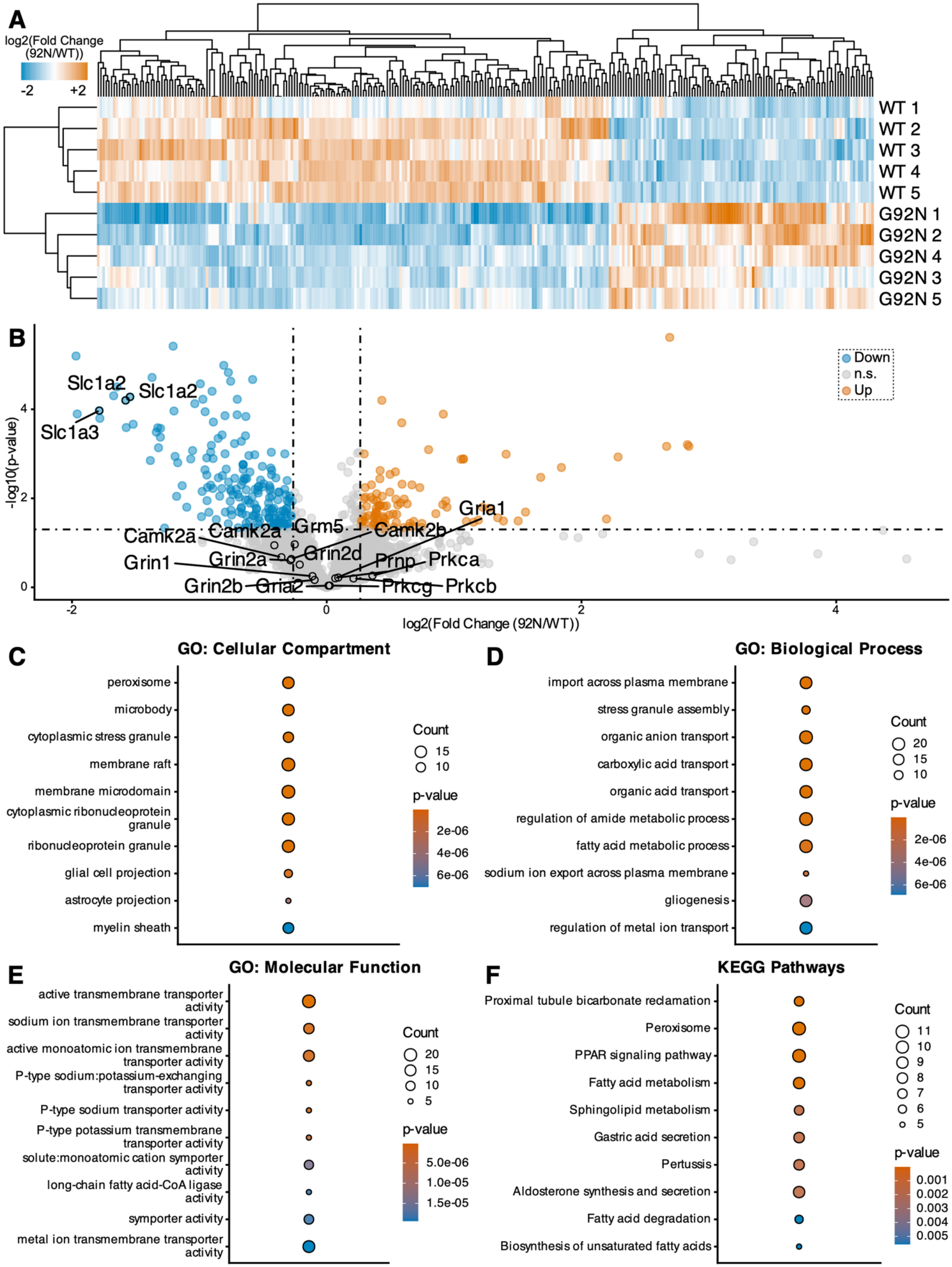

Supplemental Figure 13. *Prnp*^{92} mutation alters the synaptosomal proteome. (A) Clustered heatmap and

volcano plot depict significantly altered peptides in synaptosomes from $Prnp^{WT}$ or $Prnp^{92N}$ mice (P20) analyzed by TMT LC-MS/MS. Dashed lines indicate fold change and p-value cut-offs. Select altered peptides indicated with protein gene symbol. (C-E) Top ten most enriched “Cellular Components”, “Biological Process”, and “Molecular Function” gene ontology terms based on significantly altered peptides. Dot fill indicates significance of GO term and dot size.

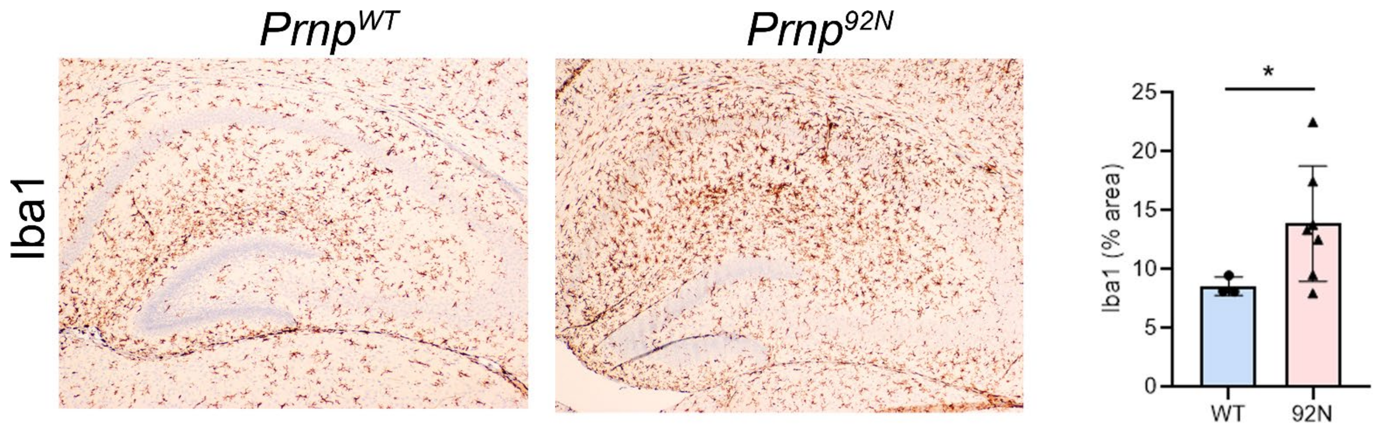

Supplemental Figure 14. Microglial reactivity evident in the hippocampus of Prnp92N mice by P10-P11. Representative immunohistochemical labeling for Iba1 in the hippocampus of *Prnp*^{WT} and *Prnp*^{92N} mice. N = 3 *Prnp*^{WT} and 7 *Prnp*^{92N}. Unpaired, two-tailed t-test with Welch's correction. *P < 0.05.

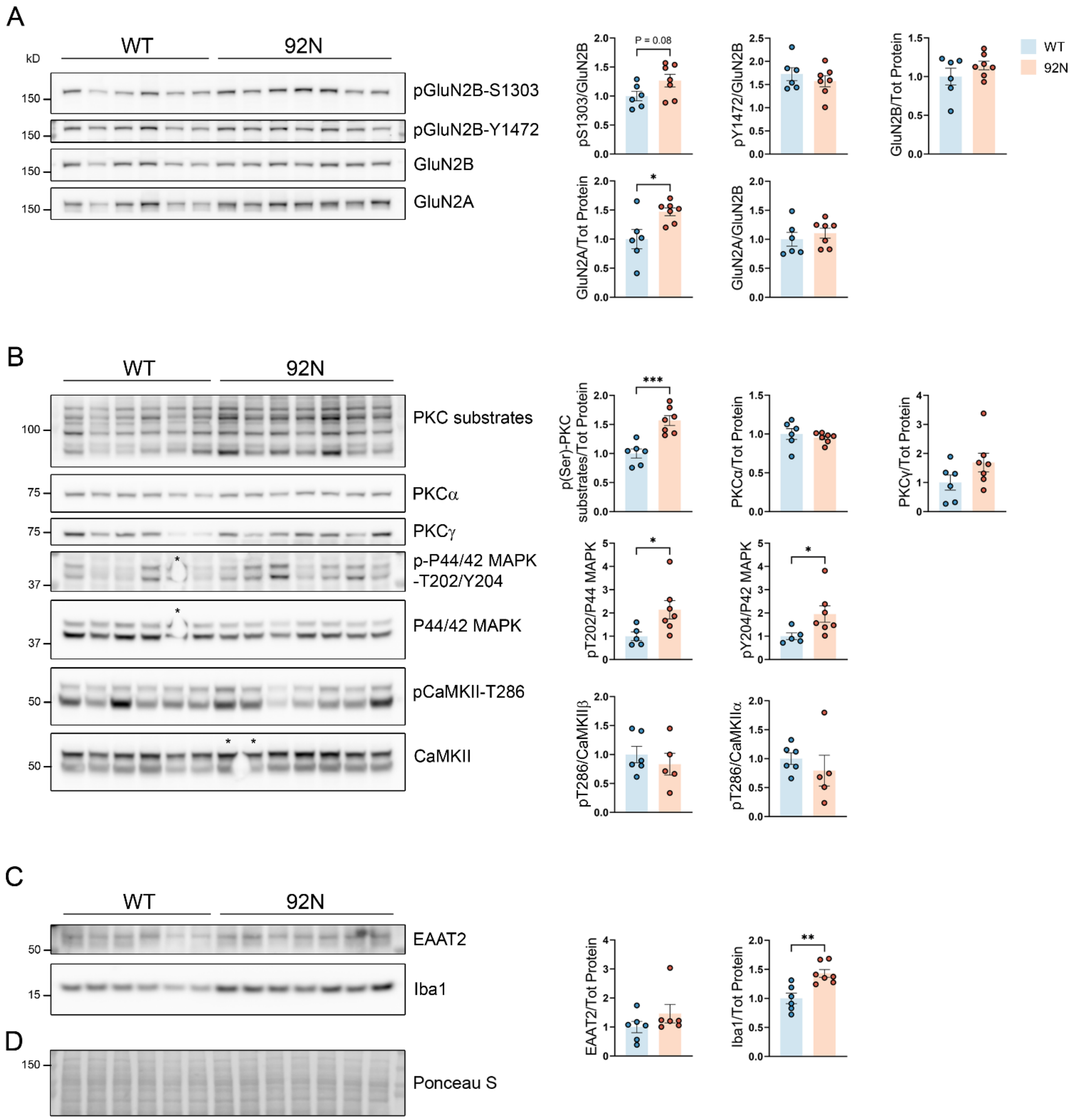

Supplemental Figure 15. Evidence of calcium sensitive kinase activity as an early alteration in the hippocampus of mice, age P10-P11. (A) Representative western blots of phosphorylated GluN2B and total NMDAR subunits, (B) phosphorylated PKC substrates, PKCα and PKCɣ levels, phospho-P44/42 MAPK, pCaMKII (C) EAAT2 and Iba1, and (D) total protein (Ponceau S). *Samples not quantified. N = 6 *Prnp*^{WT} and 7 *Prnp*^{92N}. Unpaired, two-tailed t-test with Welch's correction. *P < 0.05, **P < 0.01, and ***P < 0.001.

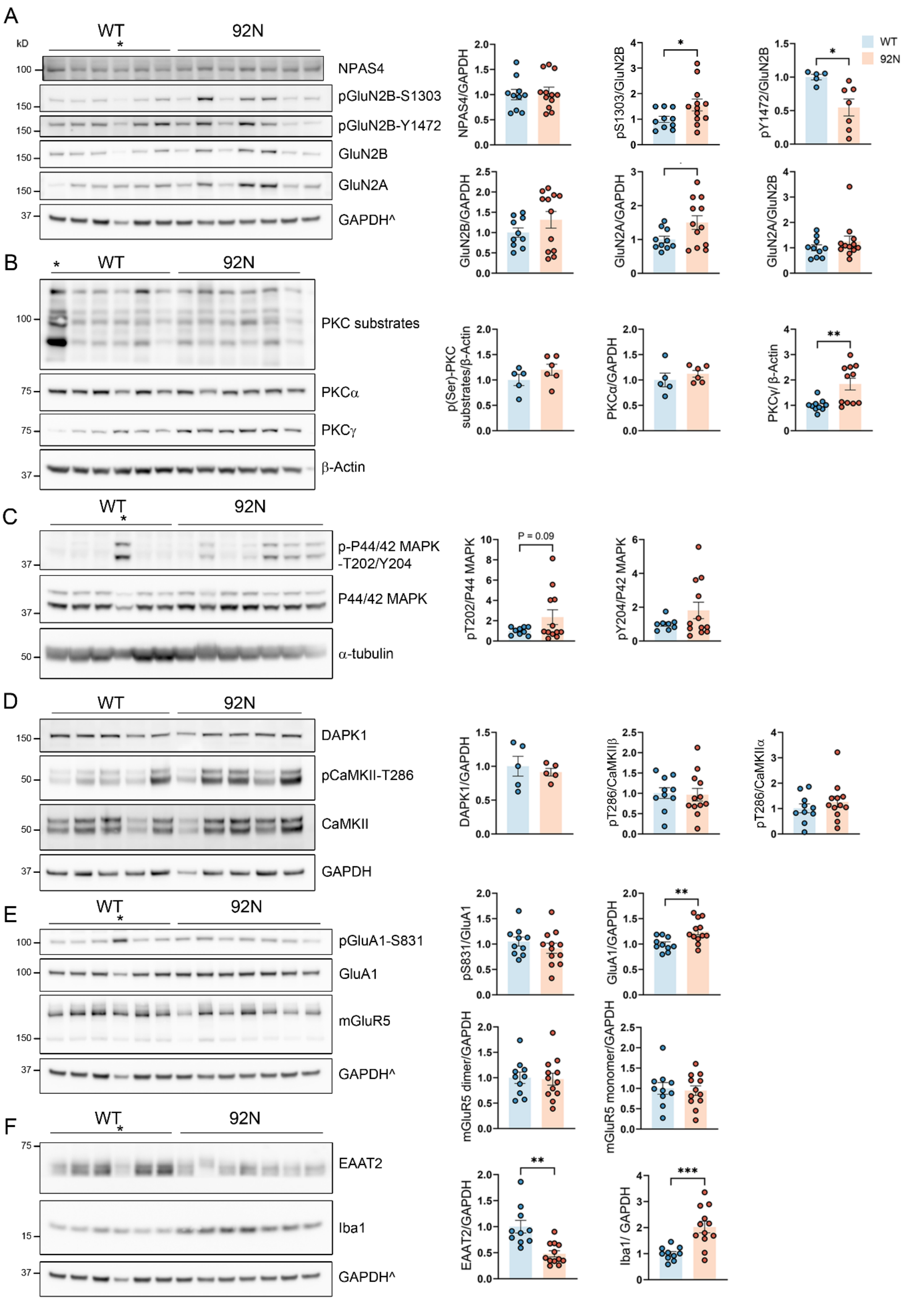

Supplemental Figure 16. Evidence of calcium sensitive kinase activity as an early alteration in the hippocampus of mice, age P15-P16. (A) Representative western blots of NPAS4, phosphorylated GluN2B and total NMDAR subunits, (B) phosphorylated PKC substrates, PKCα and PKCɣ levels, (C) phospho-P44/42 MAPK,

(D) DAPK1 and pCaMKII, (E) pGluA1-S831, GluA1, mGluR5, and (F) EAAT2 and Iba1. *This mouse was an outlier and was excluded. Blots shown are from male (A, B, C, E, F) and female (D) mice. ^GAPDH blots images are derived from the same blot as the membrane was stripped and re-probed for additional proteins. N = 5 or 10 *Prnp*^{WT} and 5 - 12 *Prnp*^{92N} male and female.

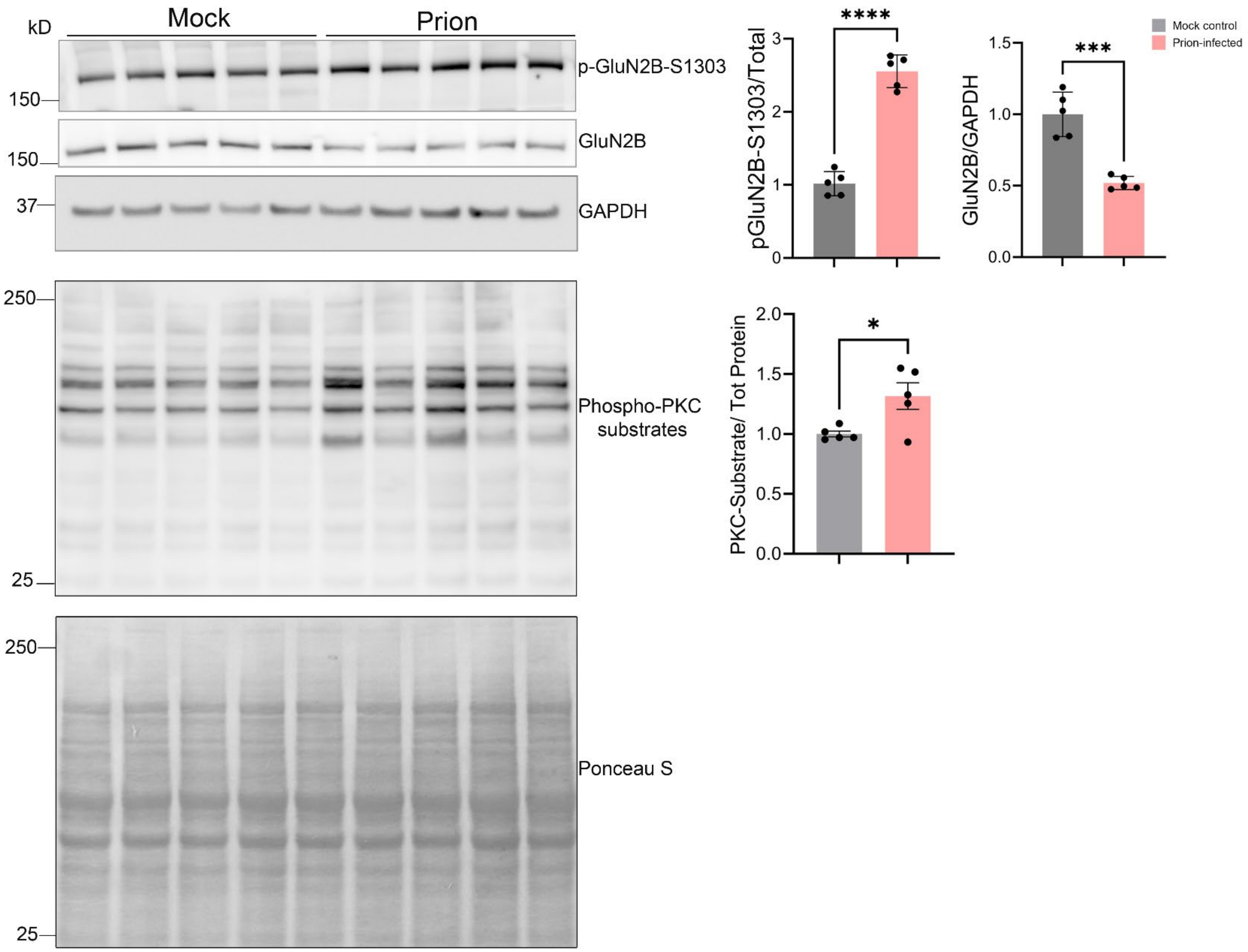

Supplemental Figure 17. Western blots of cerebral cortex from mock- or prion-inoculated (strain 22L) mice collected at approximately 80% of the incubation period (pre-terminal).

Supplemental Methods

Histopathology and immunohistochemical stains

Five micron sections were cut onto positively charged silanized glass slides and stained with hematoxylin and eosin (HE), or immunostained using antibodies against PrP (SAF84, epitope in the globular domain at the amino acids 160–170 of the mouse PrP), astrocytes (glial fibrillary acidic protein, GFAP), microglia (Iba1), myelin basic protein (MBP), and neurons (NeuN). GFAP (Dako/Agilent Z0334; 1:6000), Iba1 (Wako 019-19741; 1:3000), MBP (CST 78896; 1:100), NeuN (1:1000; Abcam ab177487) and PrP^{Sc} (Cayman Chemical; 1:1200) immunohistochemical labelling was performed on an automated tissue immunostainer (Ventana Discovery Ultra, Ventana Medical Systems). Each epitope had independently optimized retrieval parameters to yield the maximal signal to noise ratio. For PrP^{Sc}, slides were incubated in protease 2 for 20 minutes followed by antigen retrieval in CC1 (Tris-EDTA based; pH 8.5; Ventana) for 64 minutes at 95°C. For GFAP, protease antigen retrieval (P2, Ventana) was used for 16 minutes. Iba1 and NeuN retrieval consisted of CC1 for 40 minutes at 95°C. Following retrieval, antibodies were incubated on the tissue for 32 minutes at 37°C. The secondary antibody (HRP-coupled goat anti-rabbit or anti-mouse; OmniMap system; Ventana) was incubated on the sections for 12 minutes at 37°C. The primary antibody was visualized using DAB as a chromogen followed by hematoxylin as a counterstain. Slides were rinsed, dehydrated through alcohol and xylene and coverslipped.

Immunofluorescence staining of neurons

For parvalbumin (PV), somatostatin (SST), MAP2, synaptophysin, and NeuN immunolabeling, formalin-fixed, paraffin-embedded brain sections (5 µm) were deparaffinized, heated in citrate buffer (Sigma, C9999) containing 0.05% Tween20 in a pressure cooker for 30 min, quenched in 3% hydrogen peroxide in methanol for 15 min, and blocked in TNB buffer [0.5% TSA Blocking Reagent (PerkinElmer) in 100 mM Tris-HCl buffer (pH 7.5) with 150 mM NaCl] at room temperature for 1 h. Slides were then incubated in primary antibodies, anti-PV (rabbit) (Swant, PV27, 1:500), anti-MAP2 (mouse) (Sigma, MAB378; 1:200), anti-synaptophysin (Cell Signaling Technologies, D35E4, 1:250), anti-SST (mouse) (Santa Cruz, sc-55565, 1:500), and anti-NeuN (rabbit) (Cell Signaling Technologies, 24307S, 1:250) overnight at 4 °C. The PV and SST antibodies were visualized using biotin-conjugated anti-rabbit IgG and biotin-conjugated anti-mouse IgG (Jackson ImmunoResearch, 111-066-047 and 106-165-003) for 30 min, respectively, then streptavidin-HRP (Jackson ImmunoResearch, 016-030-084, 1:500) for 30 min, followed by tyramide-Alexa488 (Invitrogen, B40953). The MAP2 and NeuN antibodies were visualized using Cy3-conjugated anti-mouse IgG and Cy3-conjugated anti-rabbit IgG (Jackson ImmunoResearch, 115-165-062; 1:500 and 115-066-072; 1:1000) respectively. All slides were incubated in DAPI (Sigma, 10236276001; 1:400) and mounted using ProLong Gold (ThermoFisher Scientific, P36930).

Fluorescence photomicrographs of PV, SST, MAP2, and NeuN immunolabeled hippocampal sections were acquired with the Keyence BZX-810 fluorescence microscope using a Nikon Plan Apo λ 4x NA 0.20 air objective. All imaging functions were integrated into the Keyence BZ-X800 Analyzer software. ImageJ software was then used to hand count PV and SST labeled cells and to determine MAP2 and NeuN immunolabeled hippocampus and motor cortex areas. PV and SST counts were normalized to their respective hippocampus and motor cortex

areas in Microsoft Excel. Statistical analysis of normalized counts was performed in GraphPad Prism 8.01 (GraphPad Software, San Diego, California). Representative photomicrographs of PV, SST, MAP2, and NeuN immunolabeled hippocampal sections for figures were acquired using the Eclipse Ti2-E (Nikon) microscope. Images were acquired using the laser scanning confocal mode (A1R HD, Nikon) and a Nikon Plan Apo λ 4x NA 0.20 air objective. All imaging functions were integrated into the NIS elements software (version 5.42.02: High Content Analysis package).

Ultrastructure

Brains from *Prnp*^{92N} and *Prnp*^{WT} mice were fixed in modified Karnovsky's fixative (2.5% glutaraldehyde and 2% paraformaldehyde in 0.15 m sodium cacodylate buffer, pH 7.4) following transcardial perfusion. CA1 hippocampal sections were immersed in 1% osmium tetroxide in 0.15 m cacodylate buffer for 1 h and stained in 2% uranyl acetate for 1 h. Samples were dehydrated in ethanol, embedded in Durcupan epoxy resin (Sigma-Aldrich), sectioned at 50–60 nm on a Leica UCT ultramicrotome, and placed on Formvar and carbon-coated copper grids. Sections were stained with 2% uranyl acetate for 5 min and Sato's lead stain for 1 min. Grids were viewed using a JEOL 1200EX II (JEOL) transmission electron microscope and photographed using a Gatan digital s.

AAV transduction

To reach adequate anesthetic depth for intravenous AAV injections, P1 *Prnp*$^{-/-}$ mice were anesthetized via an ice-chilled aluminum block for 3-5 minutes until they exhibited decreased movement, whereas approximately P21 and P60 *Prnp*$^{-/-}$ mice were anesthetized with isoflurane. All mice were intravenously injected with 5 x 10^{11} genome copies (GC) per ml of AAV.PHPeB.hSyn1-PrnpWT or AAV.PHPeB.hSyn1-Prnp92N (P1 via the superficial temporal vein and P21 and older via the retrobulbar sinus)(AAV construct was bicistronic and included NeonGreen). AAV-injected animals were monitored for clinical signs daily and euthanized upon exhibiting severe signs of neurological disease (see “Mouse clinical characterization and brain collection” for list of terminal clinical signs). Survival time was calculated from the day of inoculation to the day of terminal clinical disease.

P2 and approximately P60 *Prnp*$^{-/-}$ mice were inoculated intravenously with 5 x 10^{11} GC of AAV.PHPeB.gfaABC1D-PrnpWT and AAV.PHPeB.gfaABC1D-Prnp92N. Brain was collected from each injected mouse (P2 cohort) at P16 to assess for biochemical and histologic alterations.

Memantine treatment trial

Memantine hydrochloride (Sigma M9292) was dissolved in sterile injectable 0.9% sodium chloride at 1 mg/mL, sterile filtered, and stored protected from light at 4C. Starting at P7-8, *Prnp*^{92N} and *Prnp*^{WT} were weighed once daily and received either memantine 10 mg/kg or an equivalent volume of 0.9% sodium chloride intraperitoneally twice daily (N = 15 memantine-treated *Prnp*^{92N}, n = 17 saline-treated *Prnp*^{92N}, n = 6 memantine-treated *Prnp*^{WT}, and n =5 saline-treated *Prnp*^{WT}). Animals were monitored twice daily for clinical signs and collected upon reaching terminal disease (see “Mouse clinical characterization and brain collection” for specifics on monitoring for clinical signs, definition of clinical disease, and collection of brain regions). Lifespan was determined based on when animals reached terminal disease.

Infectivity assay

Female WT (C57BL/6) mice (n = 5) were anesthetized with ketamine and xylazine and inoculated into the left parietal cortex with 30 µl of 1% brain homogenate (in PBS) prepared from a terminally ill *Prnp*^{92N} mouse. Inoculated mice were monitored three times weekly for the development of terminal prion disease, including ataxia, kyphosis, stiff tail, hind leg clasp, and hind leg paresis or other neurologic signs, and were euthanized after 500 days of age with no clinical signs. The brain was halved, and one hemisphere was immediately fixed in formalin. Fixed brains were treated for 1 h in 96% formic acid, post-fixed in formalin, cut into 2 mm transverse sections, and paraffin-embedded for histological analysis. The remaining brain tissue was frozen for biochemical studies.

Western blot of *Prnp*^{WT} and *Prnp*^{92N} whole brain homogenate

To compare total PrP^C expression and glycoform distribution in *Prnp*^{92N} and *Prnp*^{WT} brains, brain homogenate was lysed in 2% N-lauryl sarcosine containing protease (cOmplete™, Mini Protease Inhibitor Cocktail, Roche 11836170001) and phosphatase inhibitors (Pierce™ Phosphatase Inhibitor Mini Tablets, Thermo Scientific™ A32957). To assess brain for PK-resistant PrP^{Sc}, brain homogenate was lysed in 2% N-lauryl sarcosine and incubated in PBS or digested with 0.5 or 1 µg/ml PK for 30 minutes at 37 °C prior to western blotting. Membranes were probed with the anti-PrP antibody POM1 or POM19 (epitope in the globular domain) (92) and developed using chemiluminescent substrate.

The hippocampi from 10-11, 15-16 and 20 day old (n = 5 - 7) *Prnp*^{WT} or *Prnp*^{92N} mice were immediately dissected and snap-frozen in liquid nitrogen. Hippocampi were homogenized in ice-cold RIPA lysis buffer containing protease and phosphatase inhibitors and a nuclease (Benzonase) to create a 5% weight to volume (w/v) lysate (50 mM Tris HCl pH 7.4, 150 mM NaCl, 1 mM EDTA, 1% NP40, 0.5% DOC, 0.1% SDS) using a Beadbeater (BioSpec). Samples were lysed on ice for 30 min, centrifuged at 2000 x g for 5 min, and the supernatants assessed for protein concentrations by BCA assay. Samples (DTT-reduced for the 10-11 and 15-16 day old) were analyzed via western blot using the following antibodies: anti-pGluN2B-S1303 (CST 71335 and Millipore 07-398), -GluN2B (CST 14544), -pGluN2B-Y1472 (CST 4208), -GluN2A (CST 4205), -GluN1 (CST 5704), -pGluA1-S831 (CST 75574), -GluA1 (CST 8084), -pCaMKII-T286 (CST 12716), -CaMKII (CST 4436), -p(Ser) PKC substrates (CST 2261), -PKCα (CST 2056, -PKCɣ (Santa Cruz SC-211 and SC-166385) -pERK1/2 (CST 4370), -ERK1/2 (CST 4695), -EAAT2 (CST 3838), -GAPDH (Genetex GTX627408-01), -NPAS4 (Activity Signaling), -GFAP (DAKO Z0334), and -vinculin (CST 4650) and developed as described. The $sPrP^{G228}$ antibody was a gift from Drs. Markus Glatzel and Hermann Altmeppen, University of Hamburg, Germany. The chemiluminescent signals were captured and quantified using the Fuji LAS 4000 imager and Multigauge V3.0 software.

PNGase assay

To deglycosylate PrP^C, brain homogenate was incubated in a denaturing solution, heated for 10 min at 95 °C, cooled to RT, and incubated with PNGase for 24 hr at 37 °C as per instructions (New England Biolabs, 0704). Samples were then heated in LDS sample buffer at 95 °C and assessed by western blot for PrP^C (anti-PrP^C antibody, POM19 antibody).

PrP solubility assay

To measure soluble and insoluble PrP, brain homogenate having equal protein concentrations of protein in 2% N-lauryl sarcosine (final) was centrifuged at 100 g for 1 min. The supernatant was incubated at 37 °C for 30 min to solubilize membranes, and then ultracentrifuged at 200,000 x g for 1 h at 4 °C. The supernatant and pellets were collected for western blotting, with the proteins concentrated in the supernatant first by methanol-acetone precipitation.

Flotation assay

Cortices from P20 $Prnp^{WT}$ or $Prnp^{92N}$ (mixed sex) mice were homogenized with a Dounce homogenizer in TNE (25 mM Tris-HCl [pH 7.46], 150 mM NaCl, 5 mM EDTA) with protease and phosphatase inhibitors, briefly centrifuged, and the supernatant collected. To disrupt membranes, Triton X-100 (final 1%) was added to 1 mg total protein, and samples were rotated for 120 minutes at 4 °C. Extracts were mixed with two volumes of 60% OptiPrep™ (Serumwerk Bernberg AG 1893) to reach a final concentration of 40%. All lysates were loaded at the bottom of ultracentrifuge tubes (Beckman Coulter 326819) and overlayed with a 5 and 30% Optiprep® step gradient in TNE with protease and phosphatase inhibitors. Tubes were centrifuged for 24 h at 4 °C in a TLS-55 swinging bucket rotor (Beckman Coulter 346936) at 100,000 x g. Fractions (350 µl) were collected from the top of the tube and processed for immunoblotting and visualization with anti-PrP antibody POM19 (20), anti-flotillin 1 (BD Transduction Laboratories, 610820), anti-GluN2B (CST, 14544), anti-GluN1 (CST, 5704), anti-GluA1 (CST, 8084), anti-PSD95 (CST, 3450), and anti-transferrin receptor (Invitrogen 13-6800).

Flow cytometry

N2a and PrP^C-deficient RK13 cells were obtained from ATCC and cultured in low glucose DMEM with 10% FBS. RK13 cells were transfected using Lipofectamine 2000 with pcDNA3.1(+) (Invitrogen) derivative vector encoding WT-PrP^C, 92N-PrP^C, 92Q-PrP^C, or empty vector (5µg) and co-tranfected with a pEGFP-N1 (Takara) which encodes EGFP to determine transfection efficiency (0.5µg). Neuro2A cell transfected with GFP were used as positive control for PrP surface levels. After 48 hrs, cells were detached using 2.9mM EDTA (pH 6.14) and resuspended in FACS buffer (PBS + 0.1% BSA). Prior to surface staining cells were incubated with Fc blocking anti-CD16/32 antibody (BioXCell Cat#BE0307; 1:50). Cells were stained with POM1 to detect surface PrP^C or a mouse IgG Isotype control (PeproTech Cat# 500-M00). APC goat anti-mouse IgG was used as secondary antibody (Biolegend Cat# 405308) according to manufacturer's recommendation. FACS was performed using a BD FACSCanto II and data were analysed using BD FAC SDiva software version 9.0. The mean fluorescence intensity of the cells was normalized to expression of levels of WT-PrP^C, 92N-PrP^C, and 92Q-PrP^C to control for transfection efficiency across biological replicates.

Cycloheximide chase

To compare protein turnover and half-life of PrP^C-WT and PrP^C-92N, RK13 cells (which lack endogenous PrP^C expression) were transfected (Lipofectamine 3000, Thermo Fisher Scientific) with a plasmid encoding either a wild-type or 92N-mutated PrP^C protein (pCDNA3.1 backbone). 24 h after transfection, cells were treated with cycloheximide (355 uM) for 0, 0.5, 1, 2, 4, 6, 8, 10, 12, or 24 h before lysis in ice-cold RIPA buffer (50 mM Tris pH 8.0, 150 mM NaCl,

0.5% NP40, 0.5% DOC, 0.1% SDS, 1 mM EDTA, protease inhibitors, phosphatase inhibitors). Samples were analyzed via SDS-PAGE and immunoblot using the anti-PrP antibody POM19, and anti-vinculin as a loading control.

PIPLC cleavage of surface PrP^C

To quantify the cell surface expression of WT- or 92N-PrP^C, RK13 cells overexpressing WT or PrP^C-92N or primary cortical neurons isolated from *Prnp*^{92N} or *Prnp*$^{/WT}$ littermates were subjected to cleavage of surface GPI-anchored proteins using phospholipase C (PIPLC). Cortical neurons (DIV 14-16) were washed twice with PBS, then incubated in PIPLC cleavage buffer for 60 min at 37 °C (33% Opti-MEM, 66% PBS, 0.5 U/mL PIPLC). The media containing cleaved proteins was collected and centrifuged at 500 x g. The adherent cells were washed three times with PBS before lysing in ice-cold RIPA buffer (50 mM Tris pH 7.4, 150 mM NaCl, 0.5% NP40, 0.5% DOC, 0.1% SDS, 1 mM EDTA, protease inhibitors, phosphatase inhibitors). Equal volumes of PIPLC cleavage buffer and lysis buffer were used. Equal volumes were then analyzed via SDS-PAGE and immunoblot using the anti-PrP antibody POM19.

RT-QuIC analysis for PrP seeding activity

RT-QuIC reaction mix was composed of 10 mM phosphate buffer (pH 7.4), 300 mM NaCl, 0.1 mg/ml recombinant mouse PrP (residues 23-231; $rPrP^{Sen}$), 10 µM thioflavin T (ThT), 1 mM ethylenediaminetetraacetic acid tetrasodium salt (EDTA), and 0.001% SDS. Aliquots of the reaction mix (98 µl) were loaded into each well of a black 96-well plate with a clear bottom (Nunc) and seeded with 2 µl of brain tissue homogenates at different dilutions, starting at 10^{-2}. The plate was sealed (plate sealer film, Nalgene Nunc International) and incubated at 42 °C in a BMG FLUOstar Omega plate reader at cycles of 1 minute shaking (700 rpm double orbital) and 1 minute rest. ThT fluorescence measurements (450 +/- 10 nm excitation and 480 +/- 10 nm emission; bottom read) were taken every 45 minutes. ThT fluorescence threshold for a positive result was calculated as the mean of all values from negative eye tissues plus three standard deviations. For quantification, end-point dilution assays were performed using Spearman-Kärber analyses to estimate the seeding dose (± S.E.) giving ThT positivity in 50% of technical replicate wells (SD_{50}) (93).

Mass spectrometry

Prnp^{WT} or *Prnp*^{92N} littermates were euthanized at 20 days old. For whole-brain and hippocampi phosphoproteomics, brains were immediately collected and snap-frozen in liquid nitrogen for analysis. Brains were homogenized in 10 mM Tris pH 8, 150 mM NaCl, 1 mM EDTA, 2.5 mM $MgCl_2$, with protease and phosphatase inhibitors (Pierce) using a manual grinder. After homogenization, detergents were added at a final concentration of 0.1% SDS, 1% Triton-X100, 1% sodium deoxycholate. Homogenates were incubated overnight at 4 °C while rotating, then centrifuged at 4 °C for 30 min at 21,000 x g. BCA protein assay (Pierce) was performed on the supernatant and 200 µg of supernatant was precipitated using methanol and chloroform.

The precipitated proteins were digested with trypsin as previously described (94). The digested peptides were desalted, then dried in a speed-vac. Each peptide sample was labeled with a unique TMT 10-plex isobaric label (Thermo Scientific) according to a published method (95).

After the TMT labeled samples were combined into one tube, 100 µg were removed for unmodified analysis and the remaining was used for phosphorylation enrichment. Phosphopeptides were enriched using sequential ferric nitrilotriacetate (Fe-NTA) and titanium dioxide (TiO_2) using a Phosphorylation Enrichment Kit (Thermo Scientific). The two phosphorylation enrichments were combined into one sample.

The TMT unmodified and phosphorylated peptides then each fractionated off-line by high pH reverse-phase spin columns (Thermo Scientific). The TMT labeled samples were analyzed on an Orbitrap Fusion Lumos Tribrid mass spectrometer (Thermo Scientific). Samples were injected directly onto a 25 cm, 100 µm ID column packed with BEH 1.7 µm C18 resin (Waters). Samples were separated at a flow rate of 300 nL/min on an EasynLC 1200 (Thermo). Buffer A and B were 0.1% formic acid in water and 90% acetonitrile, respectively. A gradient of 1–25% B over 120 min, an increase to 40% B over 40 min, an increase to 100% B over 10 min and held at 100% B for 10 min was used, for a 180 min total run time. Peptides were eluted directly from the tip of the column and nanosprayed directly into the mass spectrometer by application of 2.5 kV voltage at the back of the column. The Lumos was operated in a data dependent mode. Full MS1 scans were collected in the Orbitrap at 120k resolution. The cycle time was set to 3 s, and within this 3 s the most abundant ions per scan were selected for CID MS/MS in the ion trap. MS3 analysis with multinotch isolation (SPS3) was utilized for detection of TMT reporter ions at 60k resolution. Monoisotopic precursor selection was enabled and dynamic exclusion was used with an exclusion duration of 10 s.

Prolucid was used to search the MS/MS data to identify peptides, phosphorylation sites were determined by the localization tool LuciPHOr (96), quantification and statistical analysis were performed using Census (97). The MS spectra was searched using the Uniprot mouse protein database with isoforms (version 2018-04-24) and a common contaminant proteins list. The decoy database was the reverse of this Uniprot database to filter identifications to a 1% FDR. The peptides were allowed to have a maximum of two miscleavages. The static modification searched were TMT on lysine and peptide N-terminal (+229.162932 Da) and cysteine carbamidomethylation (+57.021464 Da). Phosphorylation (79.9663) was searched as a differential modification on serine, threonine, and tyrosine. Reporter ion distributions specific to the lot number of the TMT reagent were employed as correction factors.

Mass spectrometry data analysis and visualization

Mass spectrometry data was further analyzed and visualized in R. Data was imported as tandem mass tag intensity values associated with Uniprot accession numbers and descriptions. Total protein intensities were determined from multiple peptides mapping to individual proteins. Phosphopeptide intensities were determined from the tandem mass tag signal from the individual peptide. Uniprot accessions associated with replicate data were removed. Data containing zero intensity values in one or more groups were removed. Individual phosphopeptide intensities were normalized to total protein abundances within individual samples. Phosphopeptides without corresponding total protein abundances were removed. Significant differences in both total protein and phosphopeptide abundances were determined using Welch's t-test and average log2 fold changes (92N/WT). A protein or peptide was considered significantly different if it had an absolute log2(Fold Change) greater than log2(1.2) and an unadjusted p-value less than 0.05. Volcano, GO ontology, and KEGG pathway plots were generated using the R package "ggplot2" (version

3.4.4). For the GO analysis, all mouse genes indexed in the R-package *org.Mm.eg.db* (Bioconductor v. 3.19) were used as the reference. Chord plot was generated using the R package "GOplot" (version 1.0.2) with manual labeling to indicate phosphopeptide phosphorylation sites. Heatmaps were calculated and generated using the R package "pheatmap" (version 1.0.12) using to default kmeans clustering. Gene symbols extracted from Uniprot accession descriptions were mapped to entrez IDs using the R package "clusterProfiler" (version 4.10.0) for use in GO ontology and KEGG pathway analysis. GO ontology analysis was performed on significantly differentially abundant proteins/peptides using the R package "clusterProfiler" (version 4.10.0). GO ontology analysis was performed on significantly differentially abundant proteins/peptides using the R package "clusterProfiler" (version 4.10.0).

Neuron culture

Primary hippocampal or cortical neurons from P0 $Prnp^{WT}$ or $Prnp^{92N}$ littermates were cultured in neurobasal plus media (Gibco) containing 2% B27 plus Supplement (Gibco) and 1X GlutaMAX™ (Gibco). The cerebral cortices or hippocampi were dissected in dissection medium (EBSS containing 6 mM MgCl2, 0.25 mM CaCl2, 0.9% glucose, 10 mM HEPES pH 7.4) and dissociated with 0.25% trypsin at 37 °C for 15 min. Cells were treated with DNase before trituration, passed through a 100 µm cell strainer, centrifuged at 150 xg for 10 min and resuspended in culture media. Neurons were grown on plates coated with poly-L-lysine. Half media changes were done every 3-4 days.

For the MK801-treated neuron experiment, primary cortical neurons (DIV 21-28) from P0 $Prnp^{WT}$ or $Prnp^{92N}$ littermates were grown on glass coverslips coated with poly-L-lysine. Cells were treated with 10 µM MK-801 (Millipore-Sigma) or with vehicle (water) for 72 h prior to fixing and staining for MAP2-positive dendrites.

Ca^{2+} imaging

To quantify cytosolic Ca^{2+}, mouse cortical neurons (DIV 14-16) were incubated with Fura-2 AM (Molecular Probes) in cell growth medium for 30 min in the dark at 37 °C, followed by incubation in fresh medium lacking Fura-2 for 15 min at 37 °C. Cells were washed twice with imaging medium magnesium free Hanks' balanced salt solution (HBSS) containing 1 mM $CaCl_2$) prior to imaging. Data were collected every 10 sec through a 10% neutral density filter. Images were collected using a 350/10 nm and a 380/10 nm excitation filter, a 450 nm dichroic mirror, and a 535/45 nm emission filter. Baseline images were acquired for 5 min before NMDA addition at 5 or 100 µM, and 10 µM glycine. All data were normalized to the baseline 350/380 of each individual neuron. Data is graphed as average +/- SEM based on individual neurons from at least six independent experiments per group. Neurons that did not respond to NMDA (relative 350/380 increase upon stimulation with 100 µM NMDA did not exceed 2) were interpreted as not being excitatory neurons expressing appropriate NMDA receptors and were excluded. Area under the curve was calculated using Graphpad Prism.

Immunofluorescent staining

Primary hippocampal and cortical neurons (DIV 21-28) from P0 $Prnp^{WT}$ or $Prnp^{92N}$ littermates were grown on glass coverslips coated with poly-L-lysine. Cells were fixed and stained with antibodies against MAP2 (green) and DAPI (blue) using the following methods: cells were

washed twice in ice cold PBS and fixed for 15 minutes in 4% paraformaldehyde. Cells were permeabilized for 5 minutes with PHEM-T buffer (60 mM PIPES pH 6.9, 25 mM HEPES, 10 mM EGTA, 2 mM $MgCl_2$, 0.5% Triton X-100). Cells were then washed three times in wash buffer (60 mM PIPES pH 6.9, 25 mM HEPES, 10 mM EGTA, 2 mM $MgCl_2$, 0.1% Triton X-100). Cells were blocked for 1 h in blocking buffer (20 mM Tris-HCl pH 7.4, 150 mM NaCl, 0.1% Triton X-100, 2% BSA, 0.1% NaN_3). Primary antibody against MAP2 (1:1000; Millipore MAB378) was incubated with fixed cells overnight at 4°C, or for 1 h at room temperature. Cells were then washed 4 times in wash buffer before adding secondary antibody (1:250; 488 anti-mouse) for 1 h at room temperature. Cells were washed 4 times in wash buffer. Coverslips were mounted on glass slides and imaged on an Olympus IX81 microscope. Dendritic beading was measured using Image J software (98). Briefly, the total length of each MAP2-positive neurite was measured, as well as the length that was visibly beaded. The beaded length was divided by the total length to obtain the percent of the projection that exhibited beaded morphology. An average percent beaded was obtained for each individual cell. Data is graphed as average +/- SEM, N = 51 - 69 from at least three biological replicates.

Neurons transfected with *Prnp*

Rat dissociated cortical neurons from P1 pups of either sex were plated at a density of 45,000 cells/cm^2 onto poly-d-lysine-coated coverslips at approximately 500,000 cells per well and maintained in B27 supplemented Neurobasal media (Invitrogen) until more than 14 d in vitro (DIV). Cells were transfected with mCherry tagged $Prnp^{WT}$ or $Prnp^{92N}$ using CalPhos™ Mammalian Transfection Kit (Takara Bio USA, Inc., 631312) for 48 h, and then immunolabelled. Neurons were fixed with a 4% PFA/sucrose solution for 10 min and permeabilized with 0.2% Triton X-100 and 2% BSA in PBS for 20 min, followed by a 1 h block in 5% BSA in PBS. Primary and secondary antibodies were diluted into 2% BSA in PBS and applied to neurons, overnight at 4°C for primary and 1 h at room temperature for secondary.

Electrophysiology

Organotypic hippocampal slices were prepared from P6-7 $Prnp^{WT}$, $Prnp^{92N}$ or $Prnp^{-/-}$ mice as described previously (99) and used at 4-7 DIV for electrophysiological recording. A surgical cut on the CA3 region was done on organotypic slices to prevent stimulus-induced bursting. Slices were transferred into the recording chamber with a continuous flow of oxygenated artificial cerebrospinal fluid (aCSF), containing 119 mM NaCl, 2.5 mM KCl, 26 mM $NaHCO_3$, 1 mM NaH_2PO4, 10 mM glucose, 4 mM $CaCl_2$, 4 mM $MgCl_2$, 10 µM gabazine and 4 µM 2-chloroadenosine (pH 7.4). The bath aCSF was perfused at a rate of 1.5-2.0 ml/min and gassed with 5% CO_2/95%O_2 at 28-32 °C. In some experiments, 3 µM Ro-25 (GluN2B inhibitor) was added to the aCSF directly. The slices were allowed to rest in the chamber for 5-10 min before all recordings. The stimulating electrode (contact Pt/Ir cluster bipolar electrodes (Frederick Haer) was placed in stratum radiatum about 300 µm down the apical dendrite of CA1 pyramidal neurons. Borosilicate glass pipettes (outer diameter: 1.5 mm, Warner, Hamden, CT) were pulled from a micropipette puller (Model P-97, Sutter). The recording electrodes had a resistance of 2-5 MΩ when filled with an internal solution containing 115 mM cesium methanesulfonate, 20 mM CsCl, 10 mM HEPES, 2.5 mM $MgCl_2$, 4 mM Na_2ATP, 0.4 mM Na_3GTP, 10 mM sodium phosphocreatine (Sigma), and 0.6 mM EGTA (Amresco), at pH 7.25. A MultiClamp 700B amplifier, an Axon Digidata 1550B, and Clampex 11 software (Molecular Devices, San Jose, CA, USA) were used

for data acquisition, digitized at 2-10 kHz, and filtered at 2 kHz. CA1 pyramidal neurons were identified under differential interference contrast microscopy. The liquid junction potential was compensated for prior to forming the cell-attached mode for all recordings. A minimum of 2 GΩ seal resistance was required before rupturing the membrane for whole-cell configuration. Evoked AMPAR or NMDAR mediated excitatory post-synaptic currents (EPSC) were recorded under voltage clamp at a holding potential of −60 mV or +40 mV, respectively. The series resistance was tested by "membrane test" in Clampex 11, and the recordings with series resistance larger than 30 MΩ or with variations larger than 30% were excluded from data analysis. Evoked responses of each recorded cell were averaged from 30–100 sweeps; amplitude and decay time were analyzed using Clampfit 11 software (Molecular Devices, San Jose, CA, USA). The AMPA to NMDA ratio was calculated by dividing the peak current amplitude recorded at a holding potential of -60mV (AMPA current) by the peak current amplitude recorded at +40mV (NMDA current).

Approval of animal studies

All animal studies were performed following procedures to minimize suffering and were approved by the Institutional Animal Care and Use Committee at UC San Diego. Protocols were performed in strict accordance with good animal practices, as described in the Guide for the Use and Care of Laboratory Animals published by the National Institutes of Health.

Data analysis, statistics and reproducibility

The number of data points and experiments performed were determined by the laboratory's previous experience and by the literature in which similar experiments were conducted (70, 100). Animals were randomly assigned, with consideration of maintaining a similar male – female balance. Pooled neurons were randomly divided and plated for treated and control groups. One-way and two-way ANOVA with Tukey's multiple comparison post-hoc test was used to compare normally distributed continuous data (PrP^C expression and glycoforms, excitatory postsynaptic currents, soluble and insoluble PrP in brain lysate from uninfected and prion-infected $Prnp^{WT}$ mice, in vitro cortical and hippocampal neuronal beading, flotation assay). Two-way ANOVA with Šidák's multiple comparisons test was used when comparing the weights of $Prnp^{WT}$ and $Prnp^{92N}$ mice. Log-rank (Mantel-Cox) test was used to assess the survival differences between AAV-transduced mice and memantine-treated and vehicle-treated mice. Means of two groups were compared using an unpaired, two-tailed *t*-test [Fura-2 experiments (cytosolic Ca^{2+} concentrations, the area under the curve, and the 340/380 ratio) and RK13 cells expressing mutant or WT *Prnp*] with Welch's correction added when group sizes varied (protein levels by western blotting, immunohistochemistry, and immunofluorescence staining). Data (summary) are shown as mean ± SEM. Statistical analysis was performed using Prism 10 (GraphPad Software). For all analyses, $p \leq 0.05$ was considered significant.
