# Supplementary material for: Mutant prion protein enhances NMDA receptor activity, activates PKC, and triggers rapid excitotoxicity in mice
Source: J Clin Invest. 2025 Apr 4;135(10):e186432. doi: 10.1172/JCI186432 (PMC12077891; doi:10.1172/JCI186432)
Supplement: Unedited blot and gel images [file jci-135-186432-s207.pdf]

Figure 1B

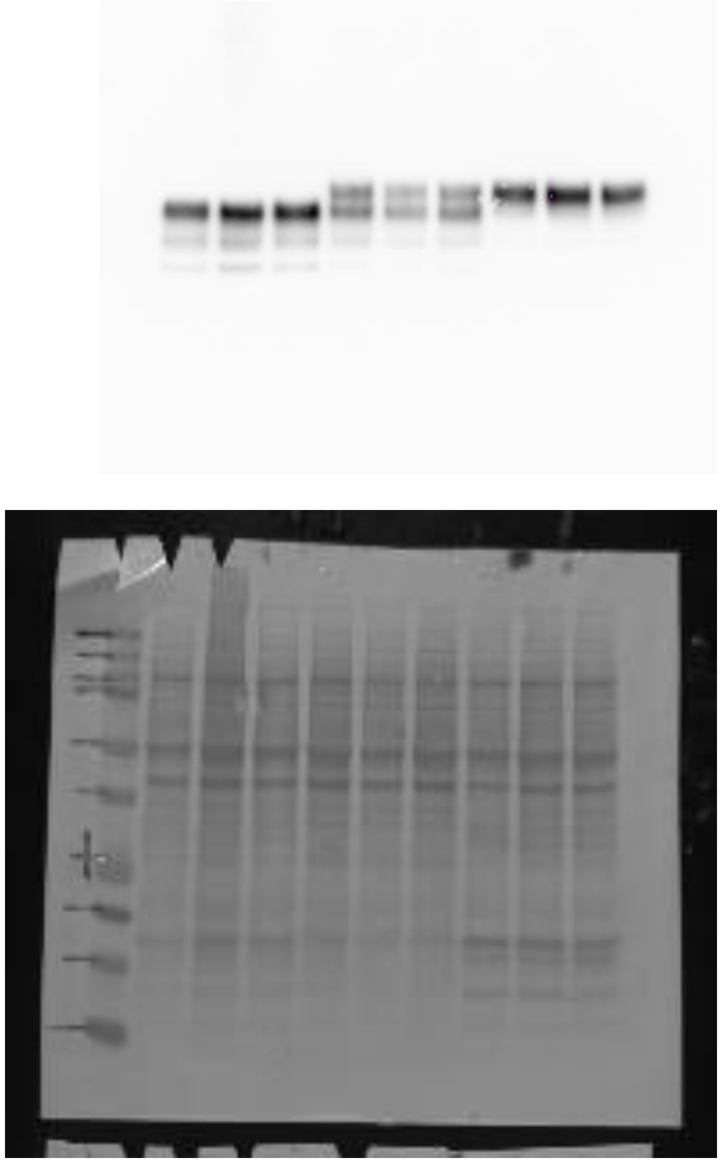

PrPC

Ponceau

Figure 2B

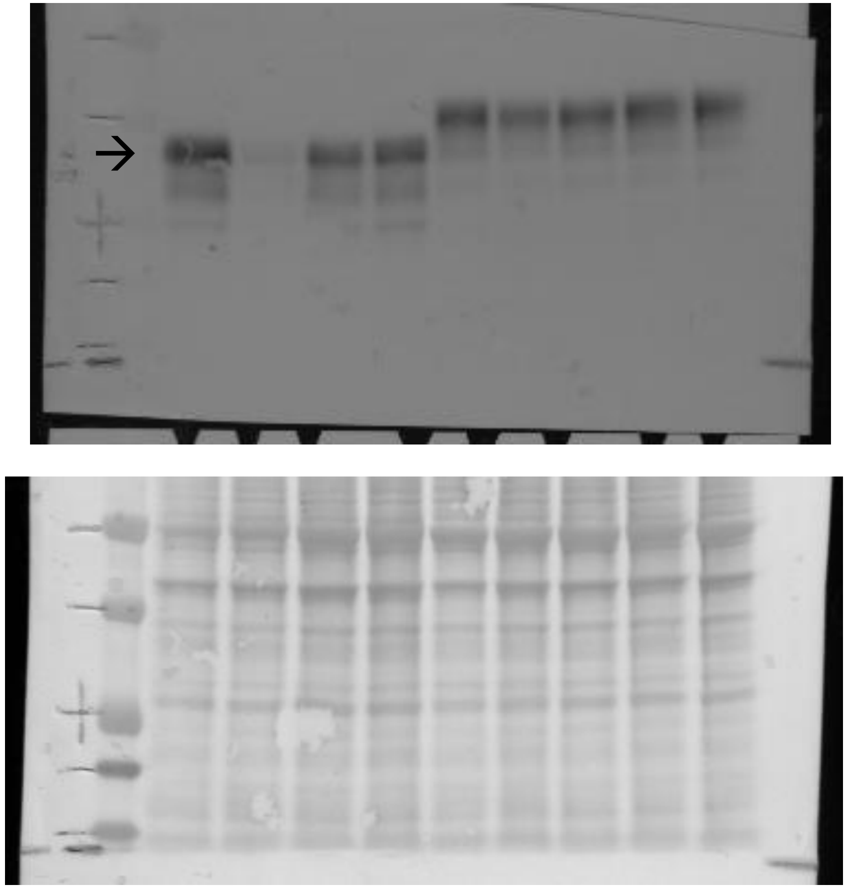

PrPC
marker superimposed

Ponceau

Figure 4A

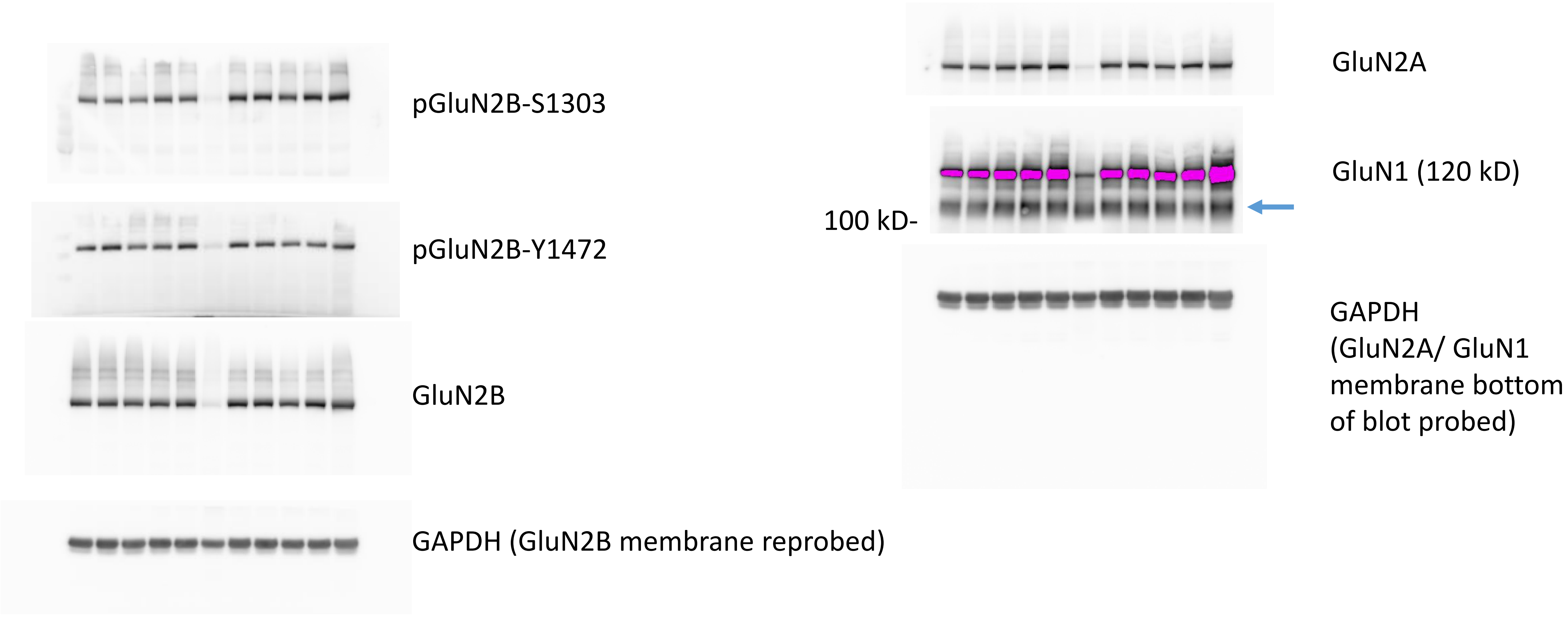
pGluN2B-S1303
pGluN2B-Y1472
GluN2B
GAPDH (GluN2B membrane reprobed)
GluN2A
GluN1 (120 kD)
100 kD-
GAPDH
(GluN2A/ GluN1
membrane bottom
of blot probed)

Figure 4B

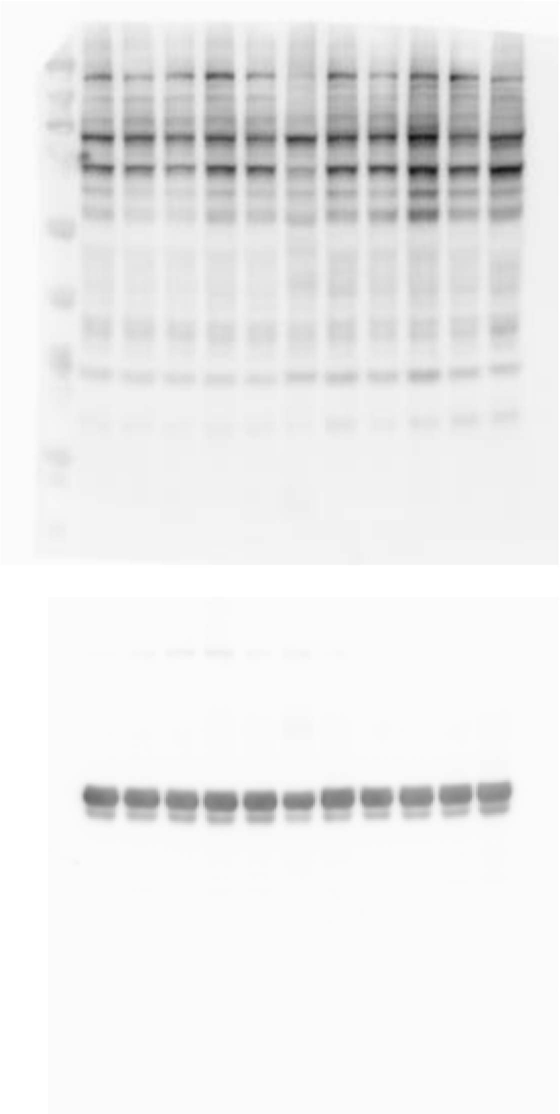

pSer PKC
Substrate

GAPDH

Figure 4C

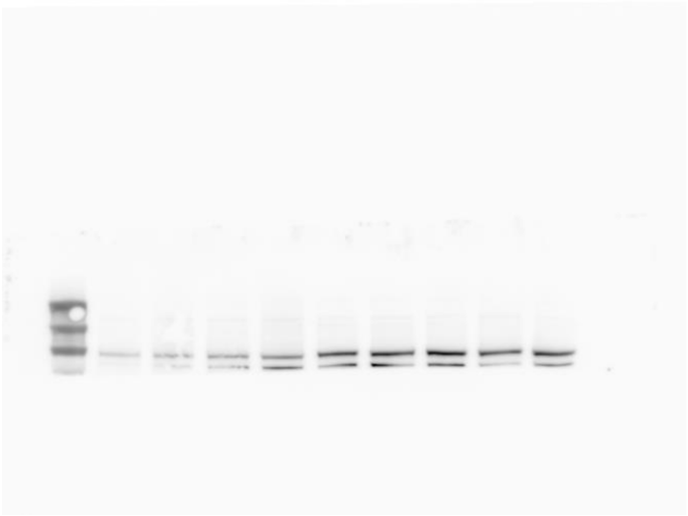

NPAS4

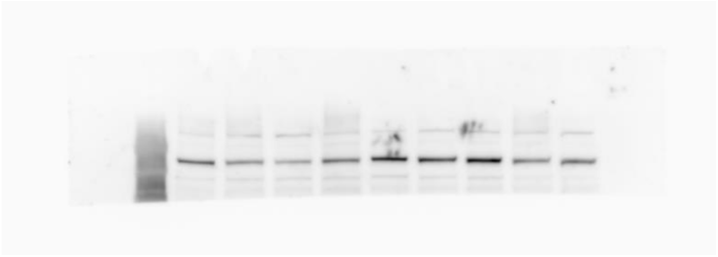

p1303-GluN2B

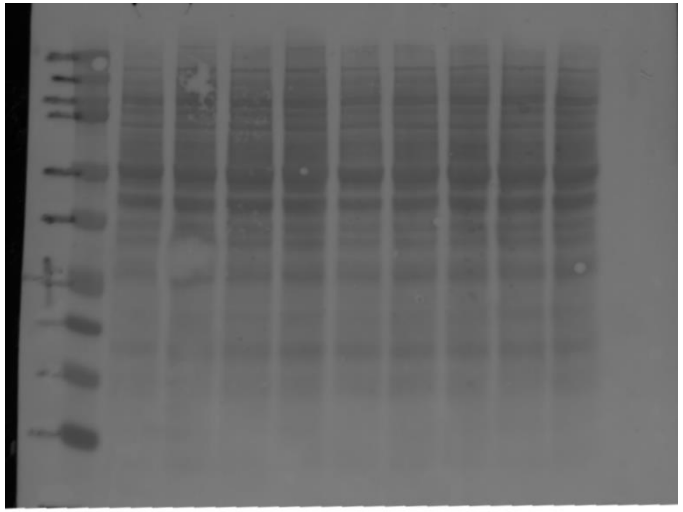

Ponceau

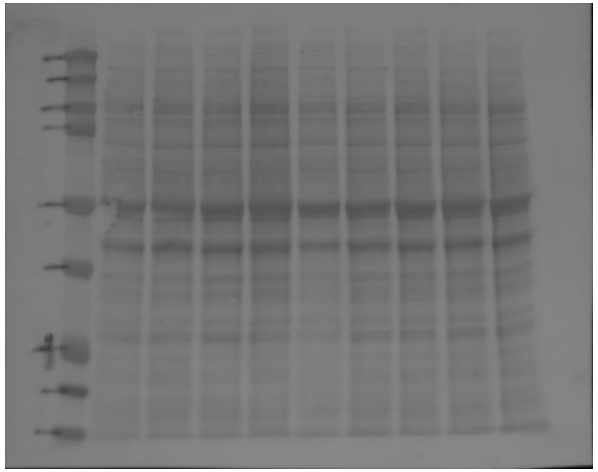

Ponceau

Figure 4C

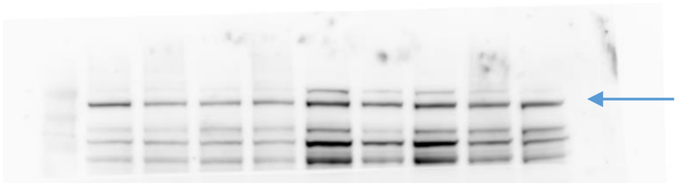

p1472-Glun2B

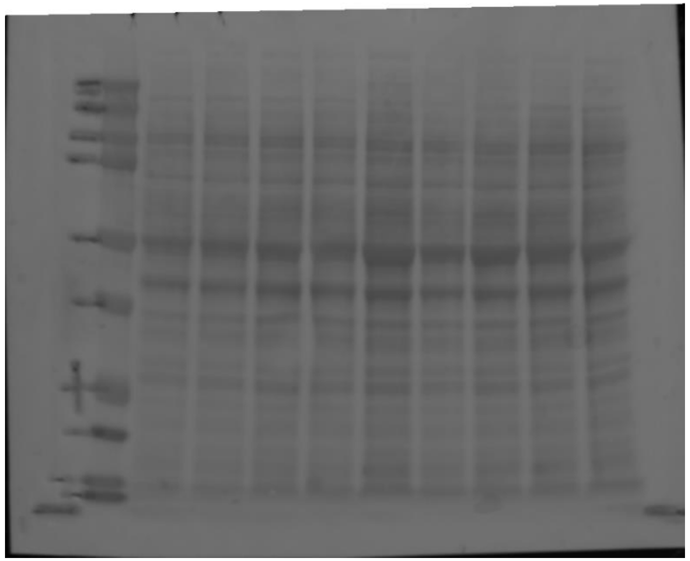

Ponceau

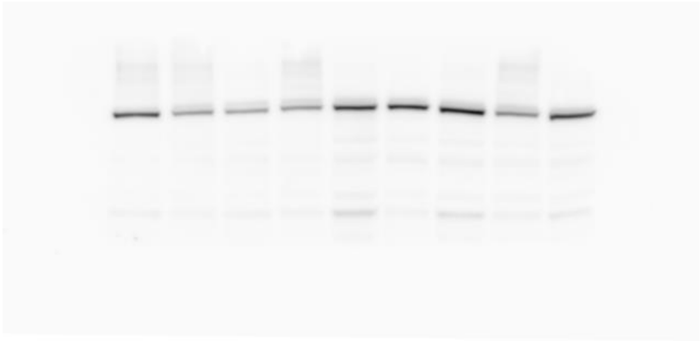

GluN2B

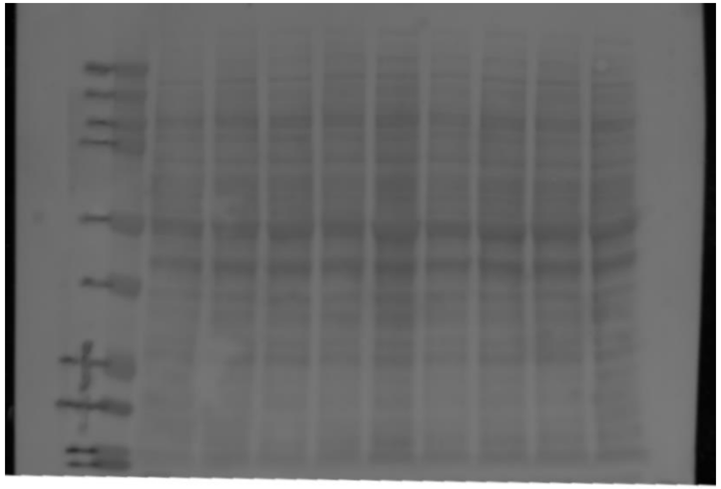

Ponceau

Figure 4C

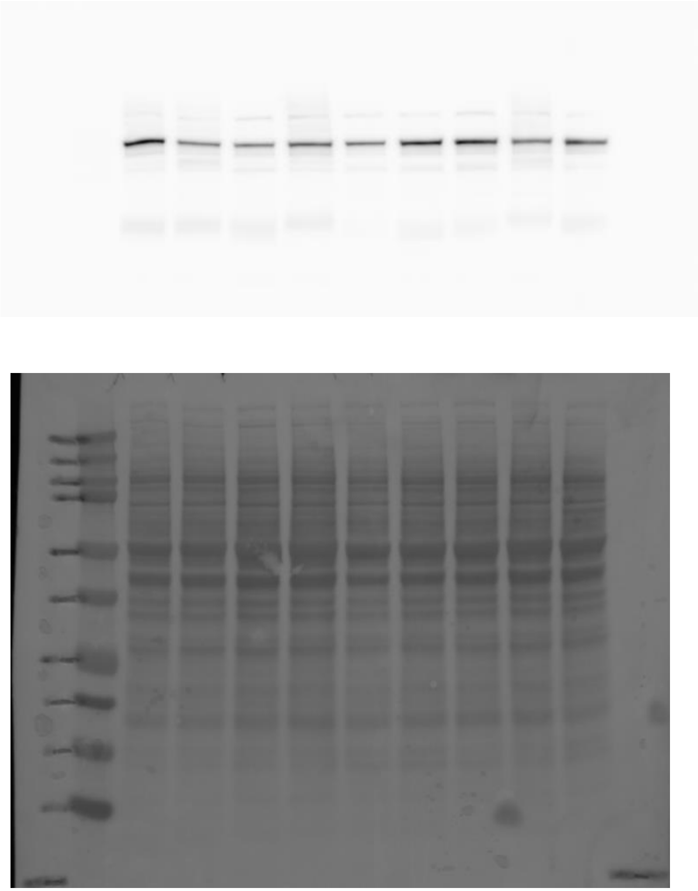

GluN2A

Ponceau

Figure 4D

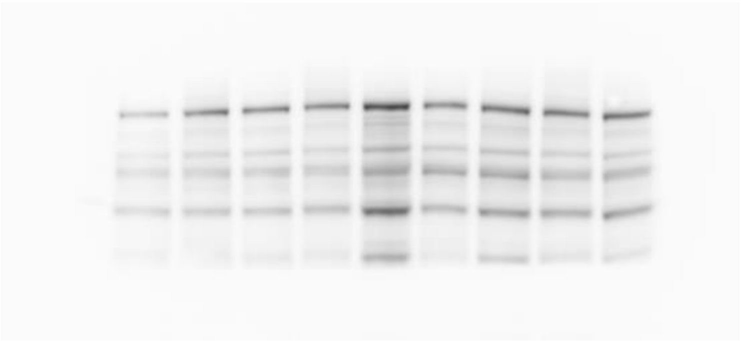

PKC substrate

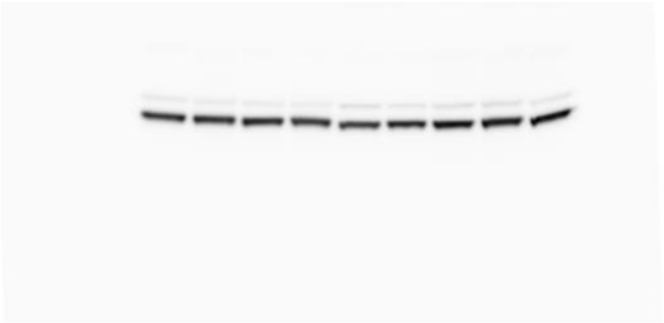

ERK (P44/42 MAPK)

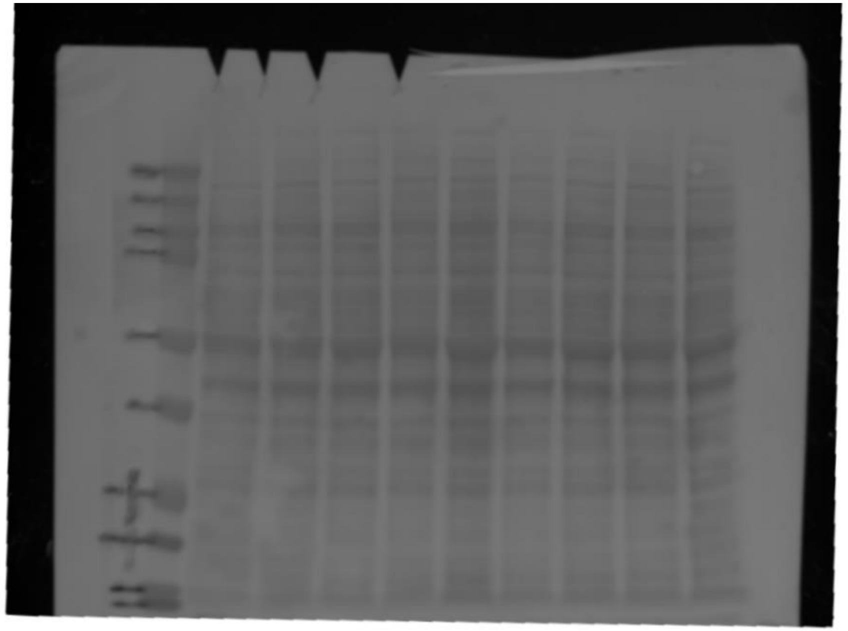

Ponceau

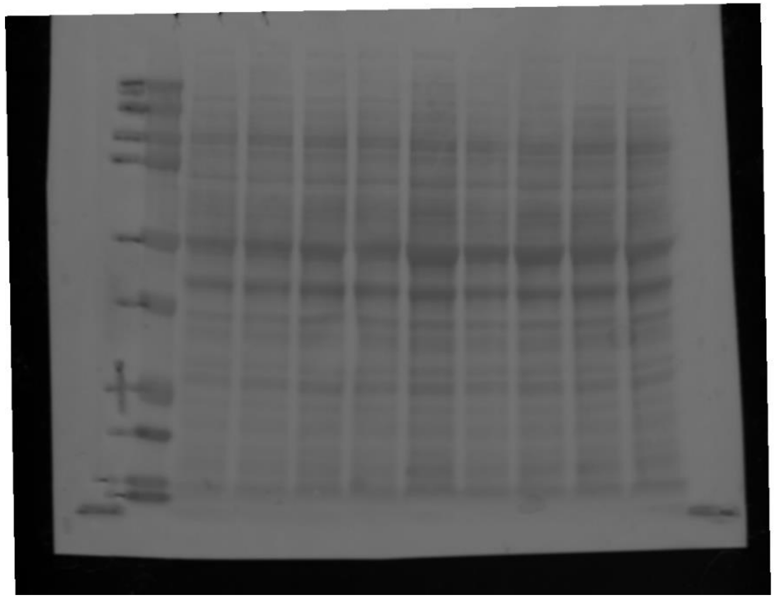

Ponceau

Figure 4D

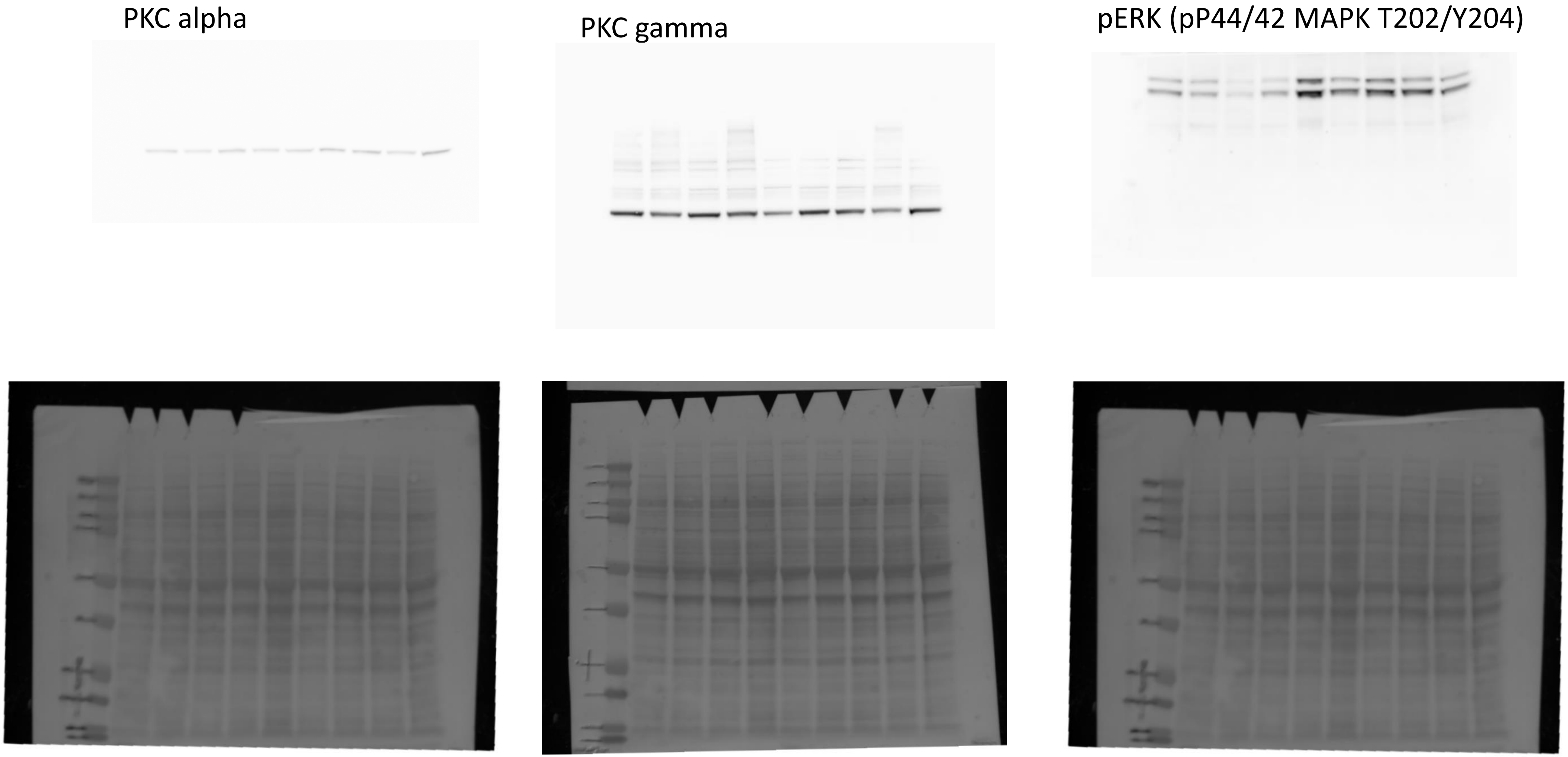

Figure 5A, WT

Higher fractions not shown in figure as denoted in the figure legend.

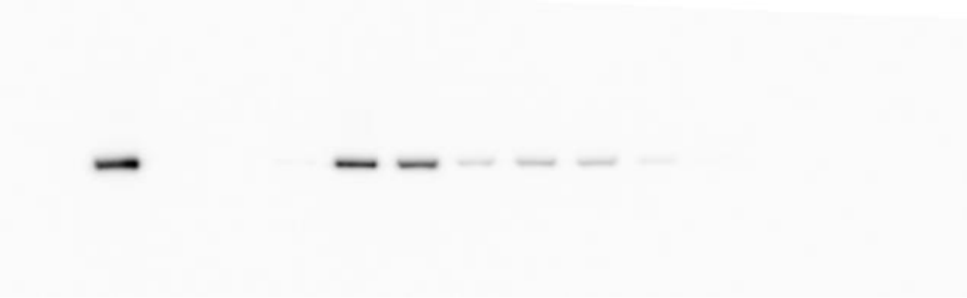

GluN2B

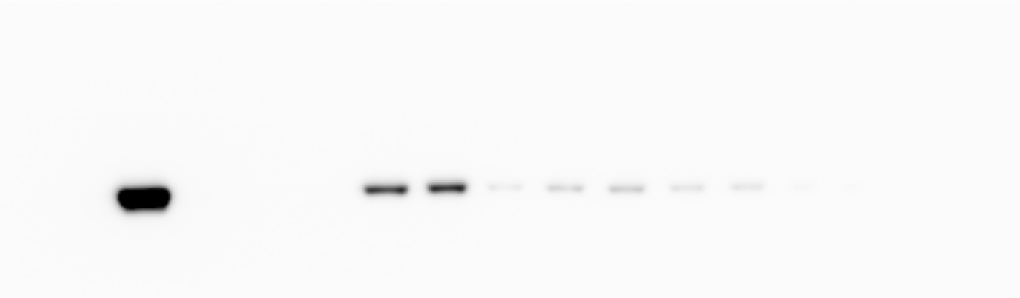

PSD95

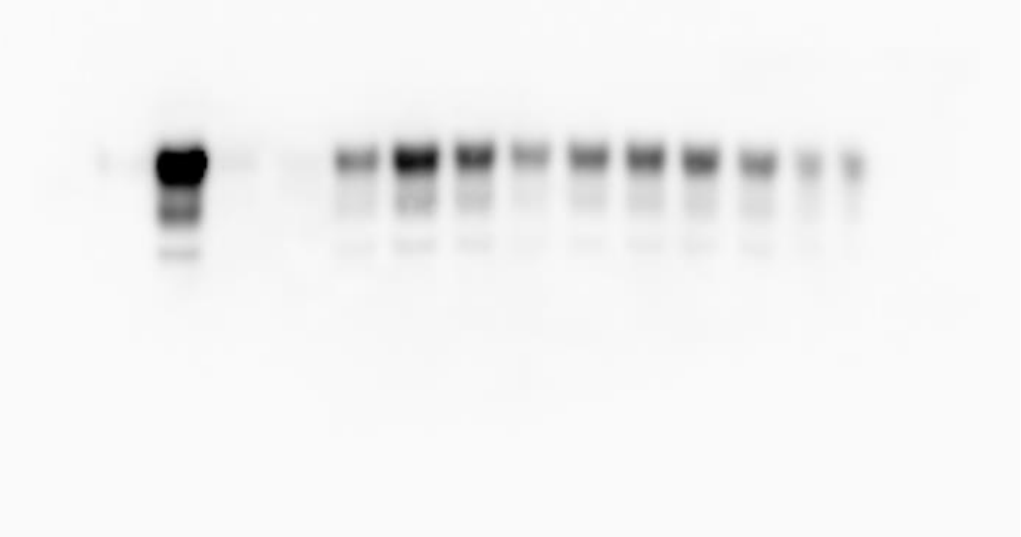

POM19

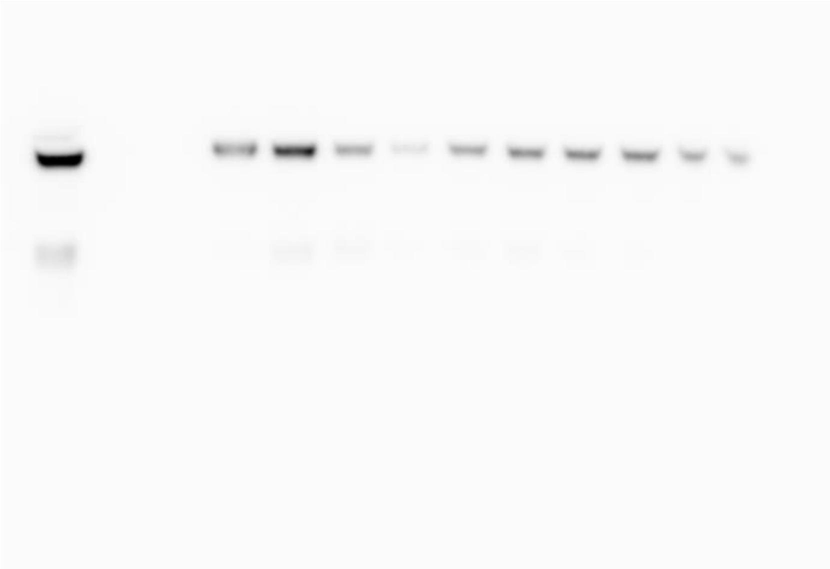

Flotillin-1

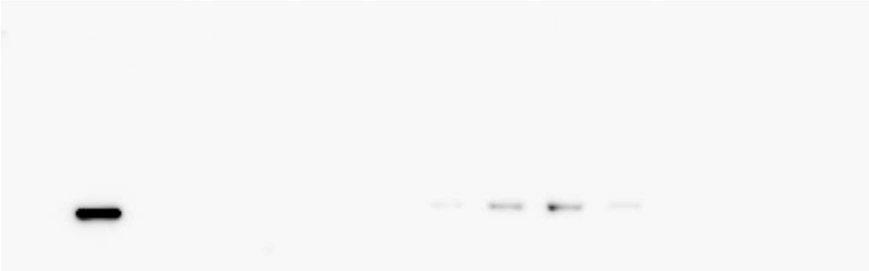

Transferrin receptor

Figure 5A, 92N

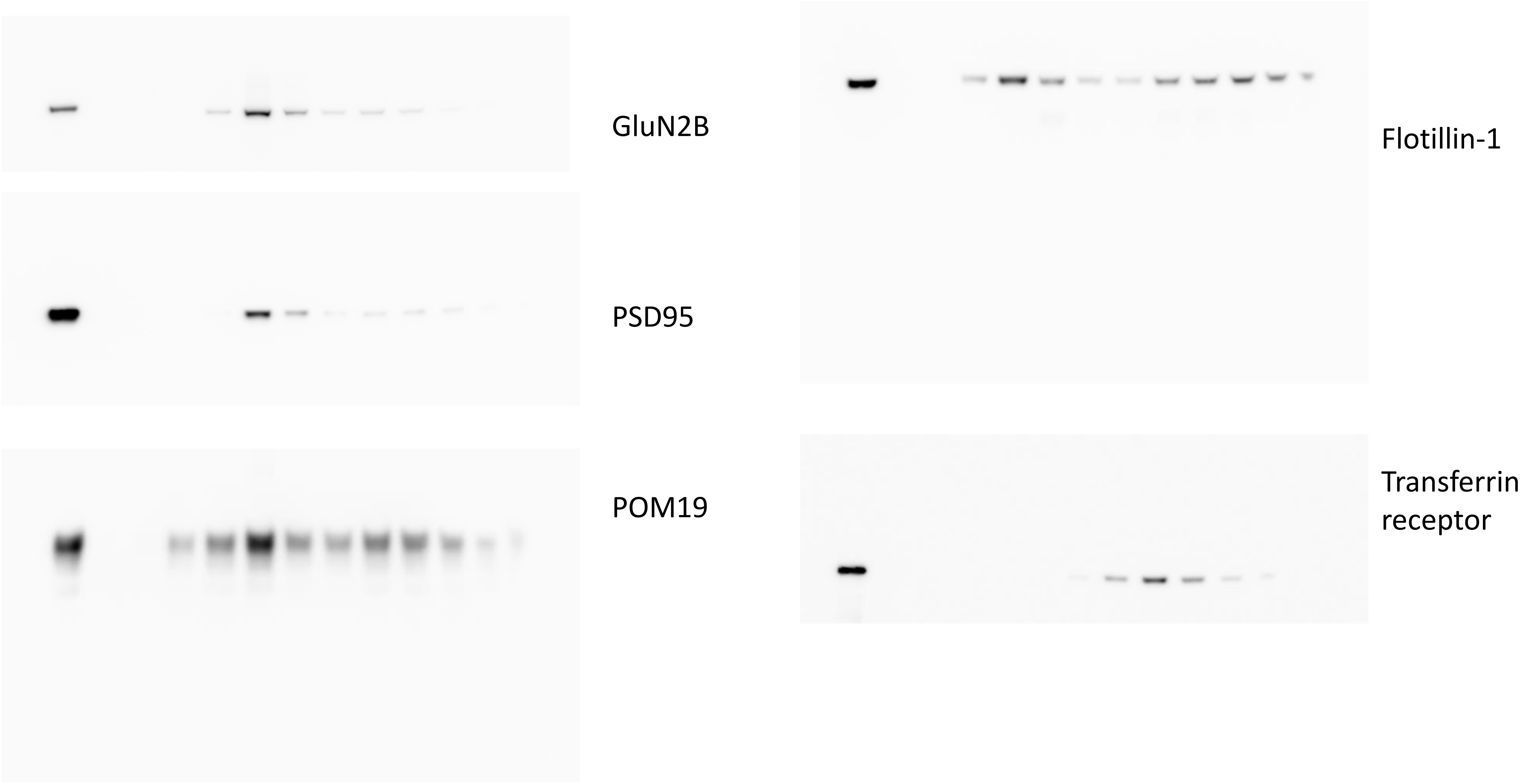

Supplemental Figure 1A

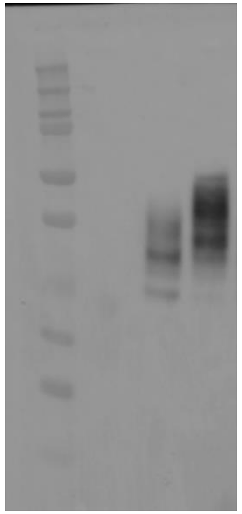

Supplemental Figure 1B

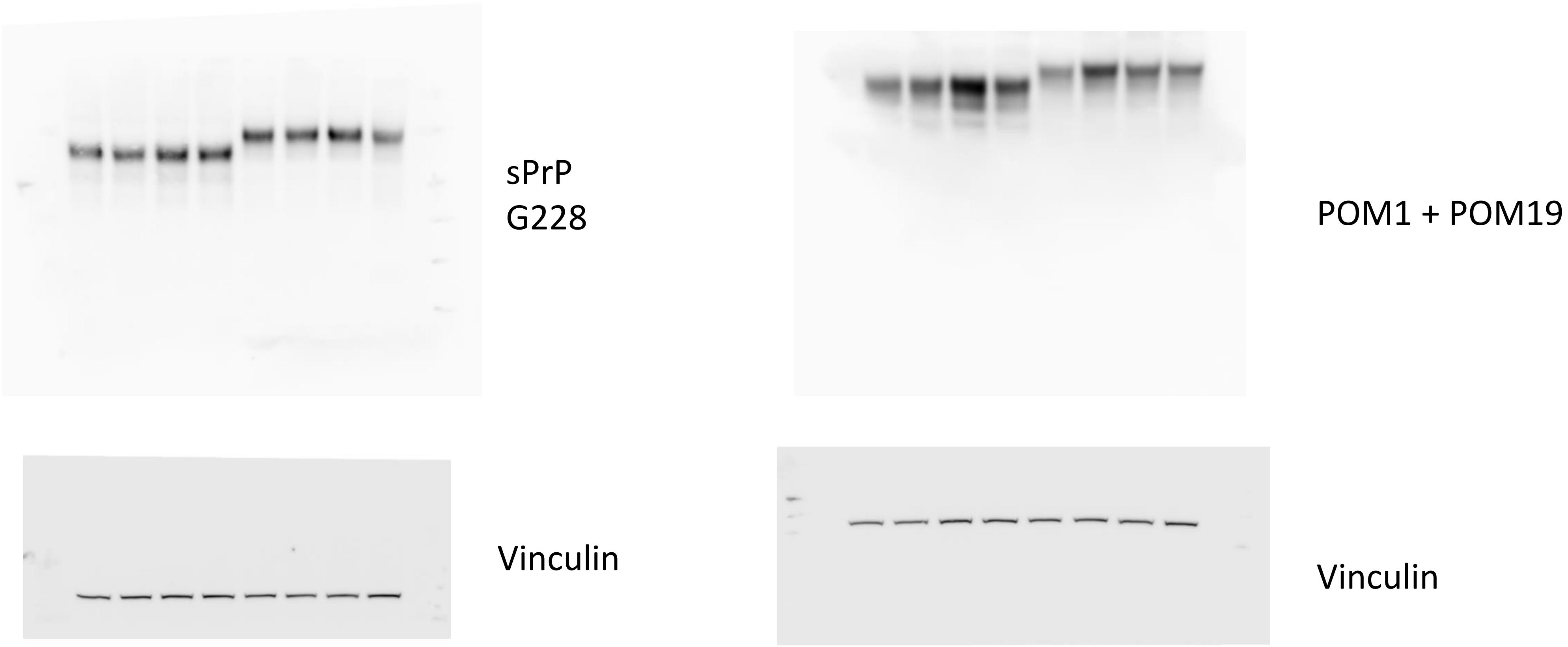

Supplemental Figure 1C

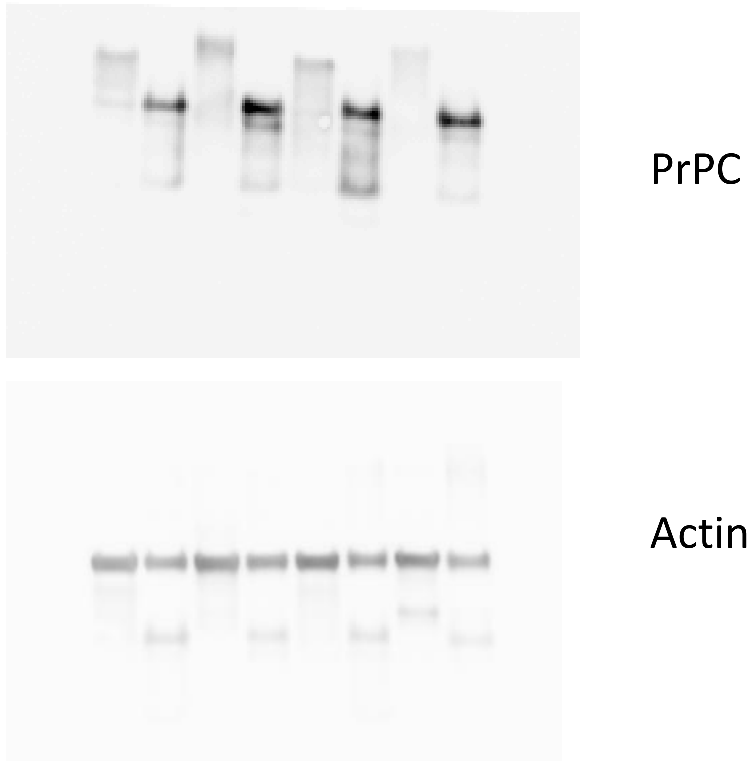

Supplemental Figure 1E

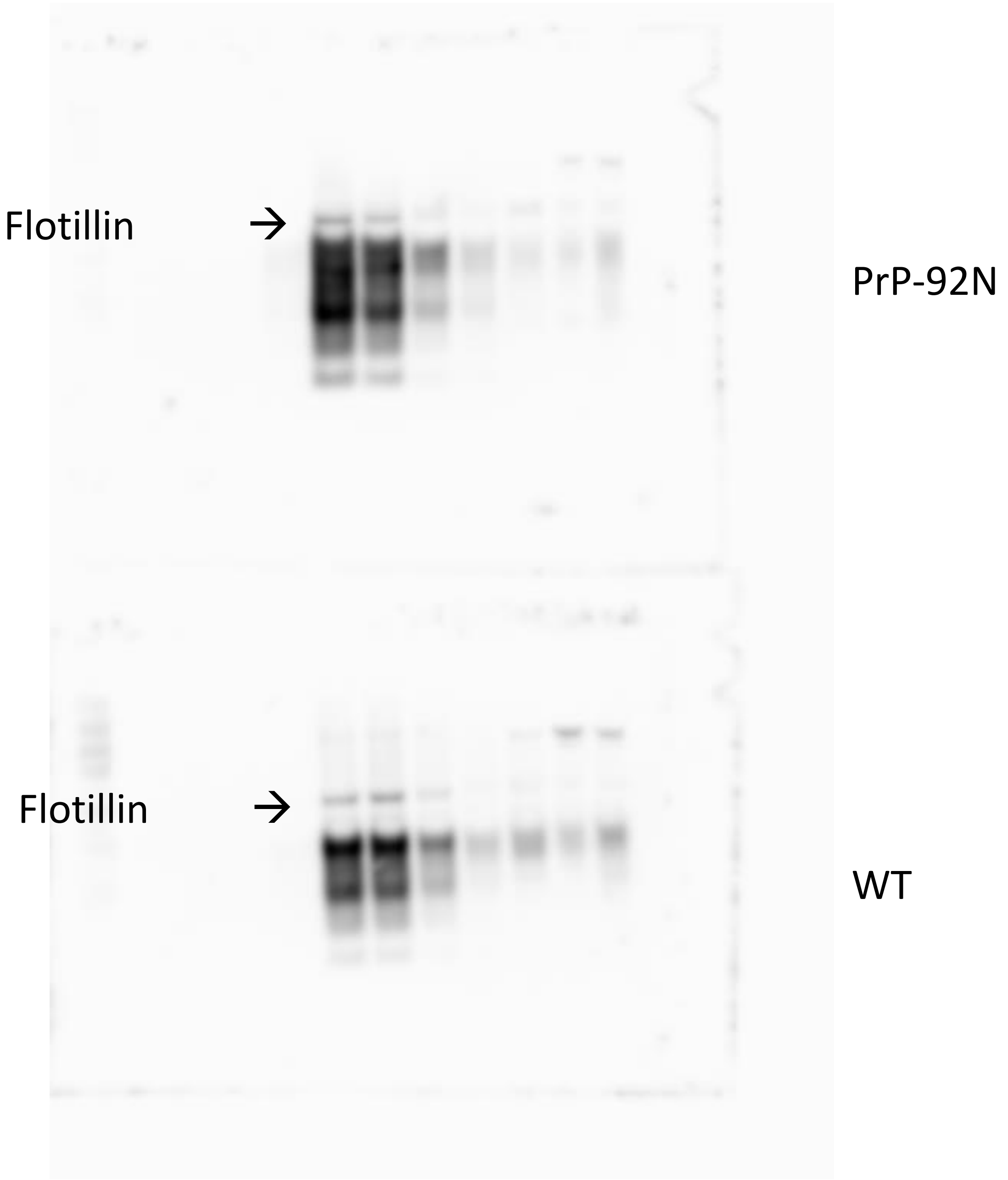

Supplemental Figure 1F

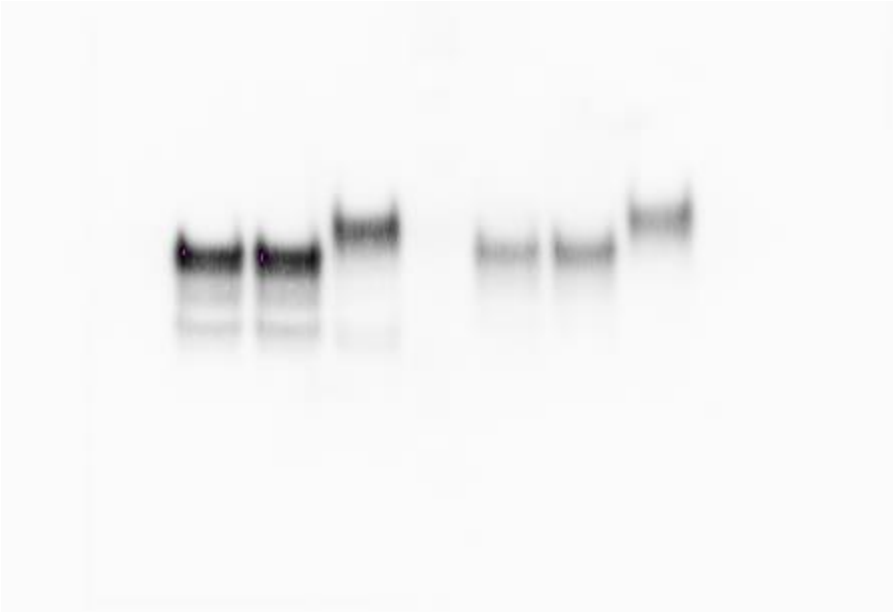

Supplemental Figure 1G

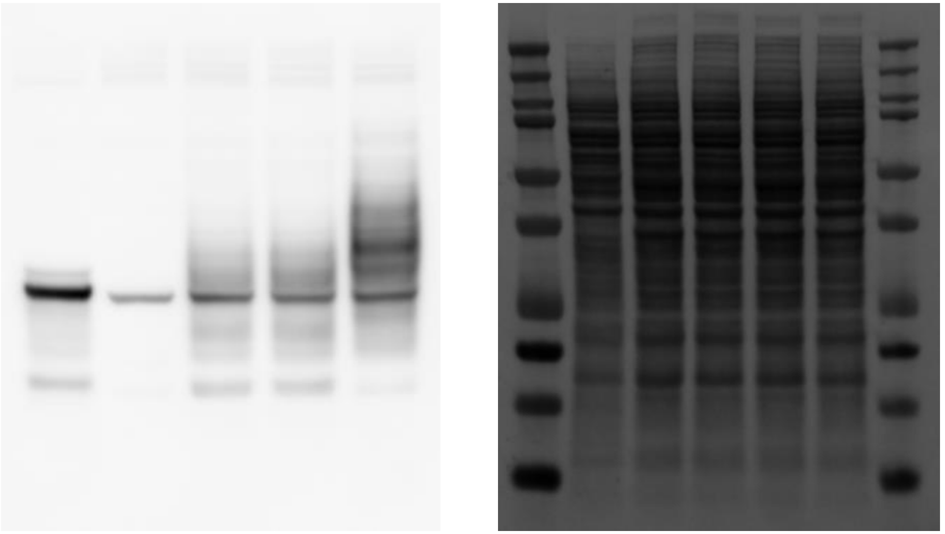

Supplemental Figure 1H

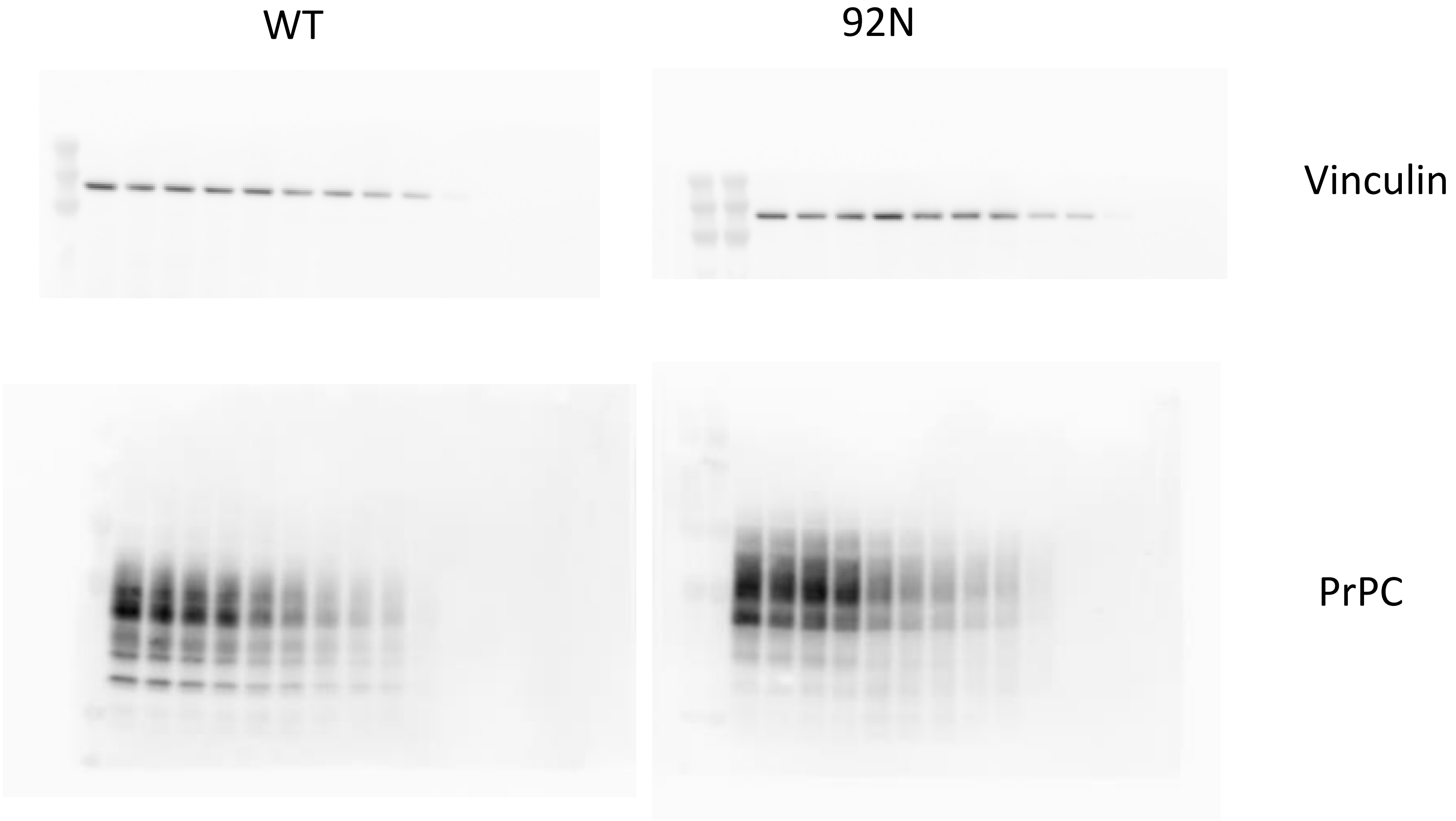

Supplemental Figure 3A

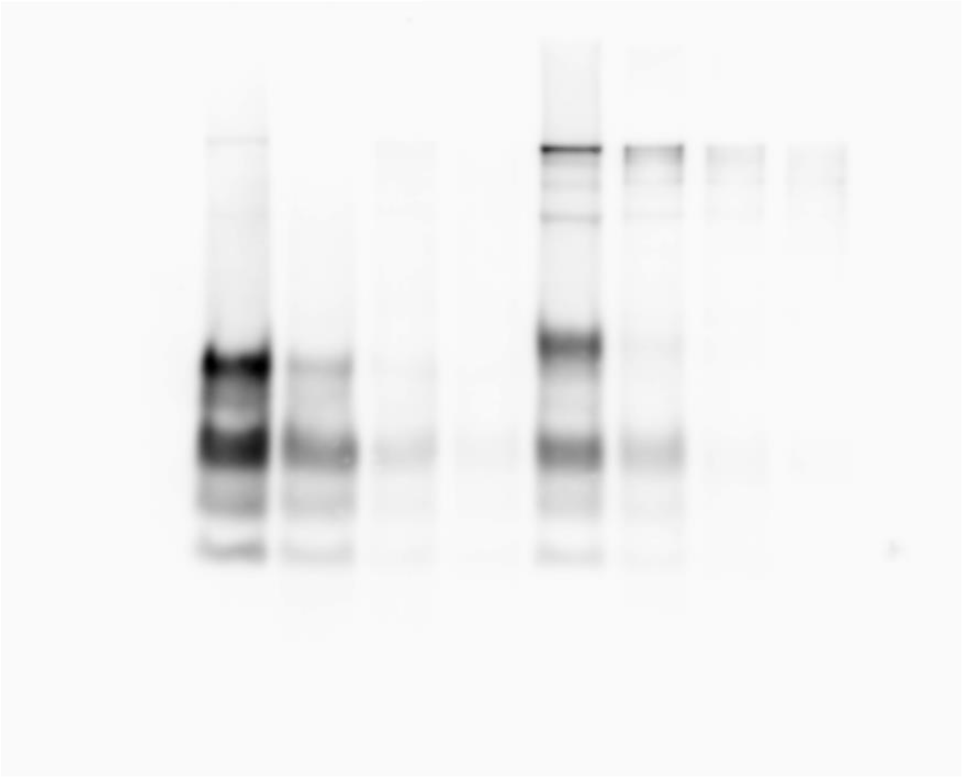

Supplemental Figure 3B

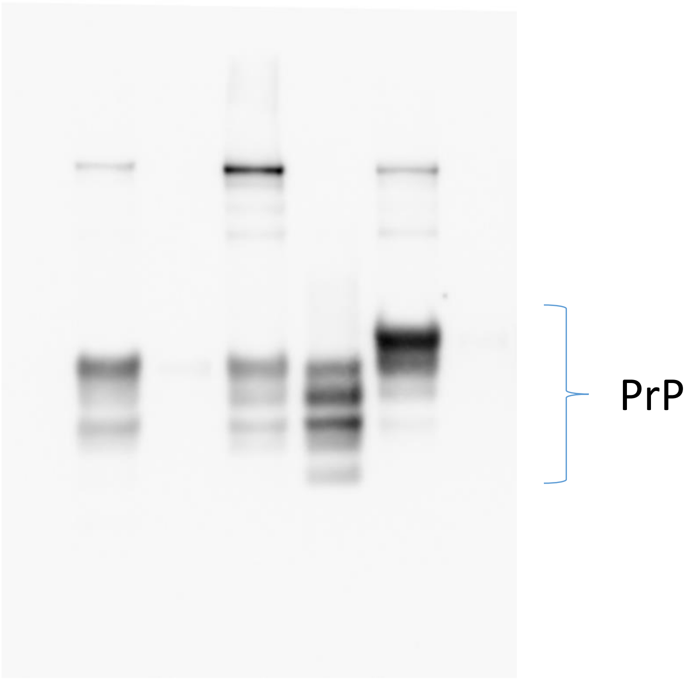

Supplemental Figure 3C

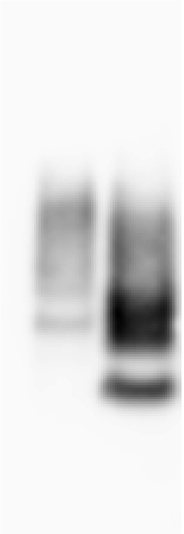

no seed and RML

Left panel

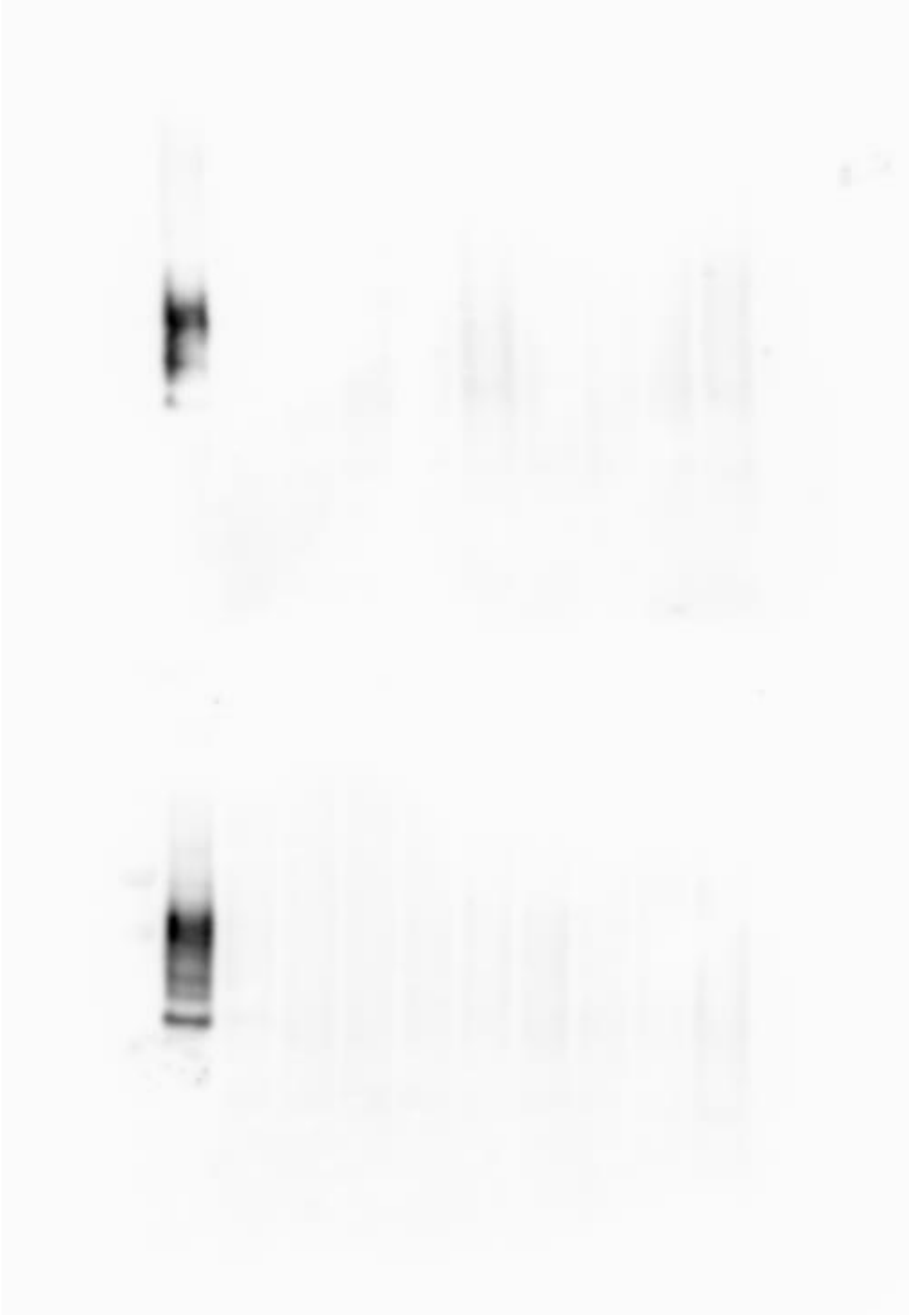

Middle panel

Right panel

Supplemental Figure 3E

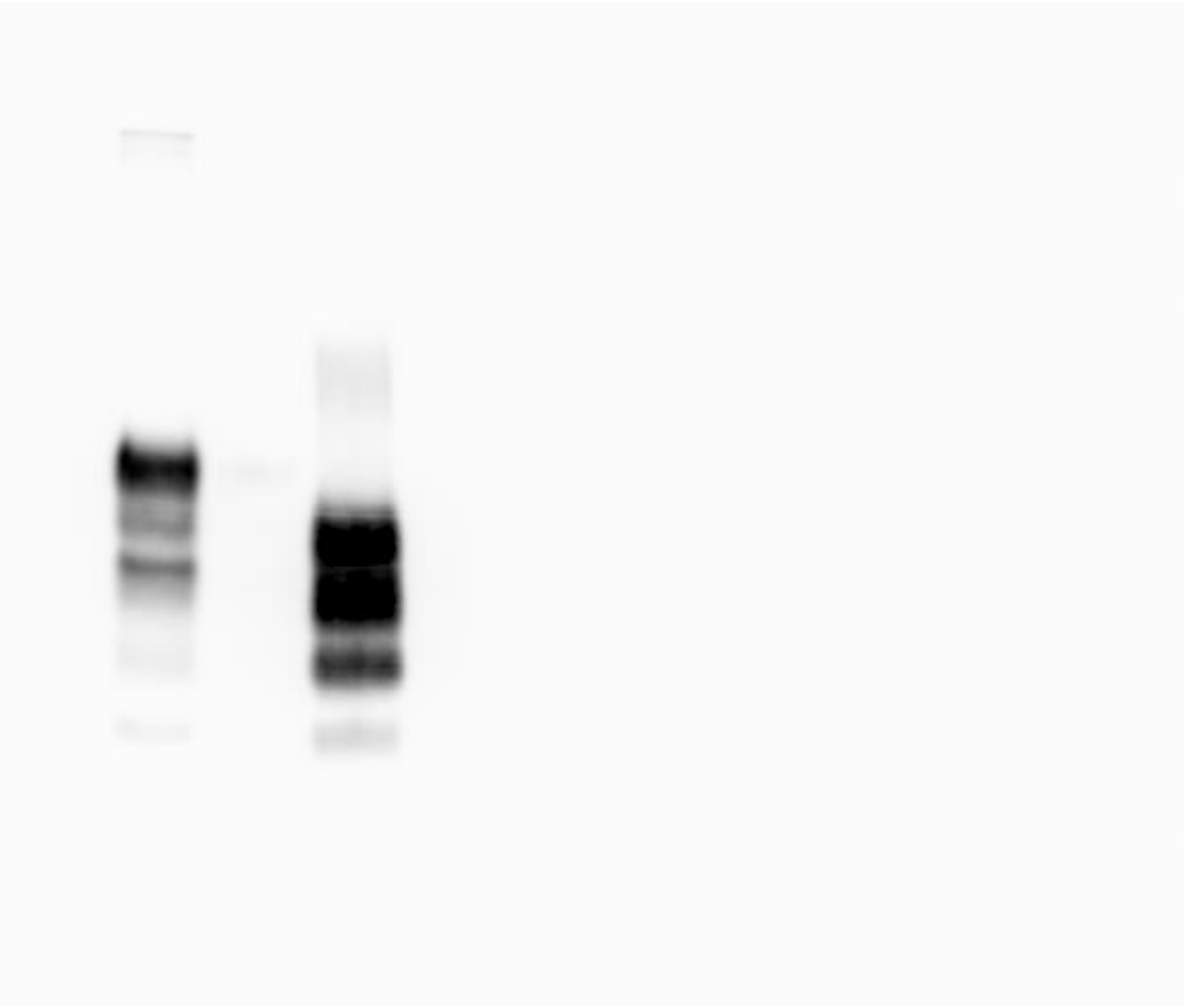

Supplemental Figure 8

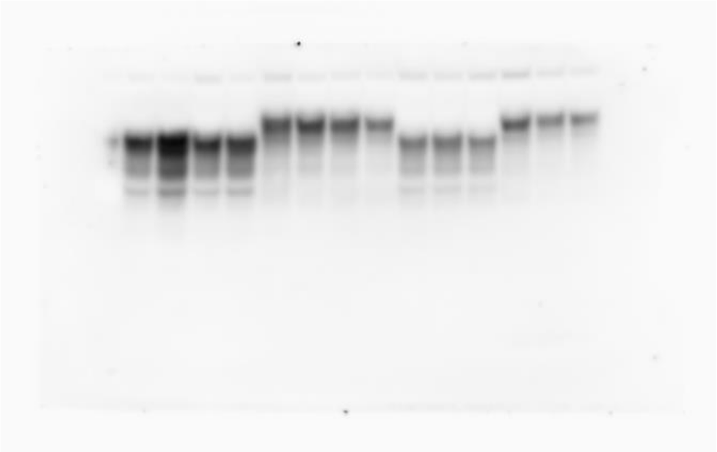

PrPC (POM1)

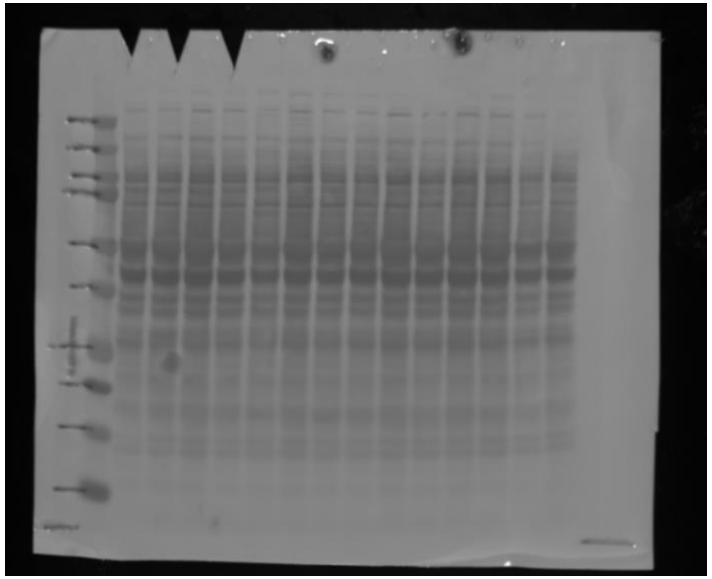

Ponceau

Supplemental Figure 9

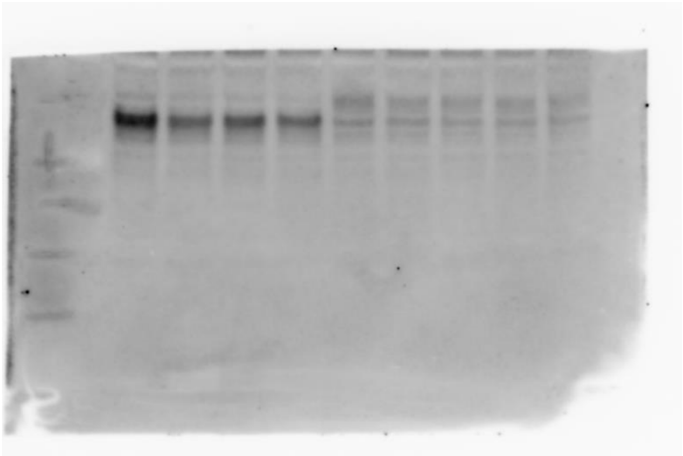

PrPC (POM1 + POM19)

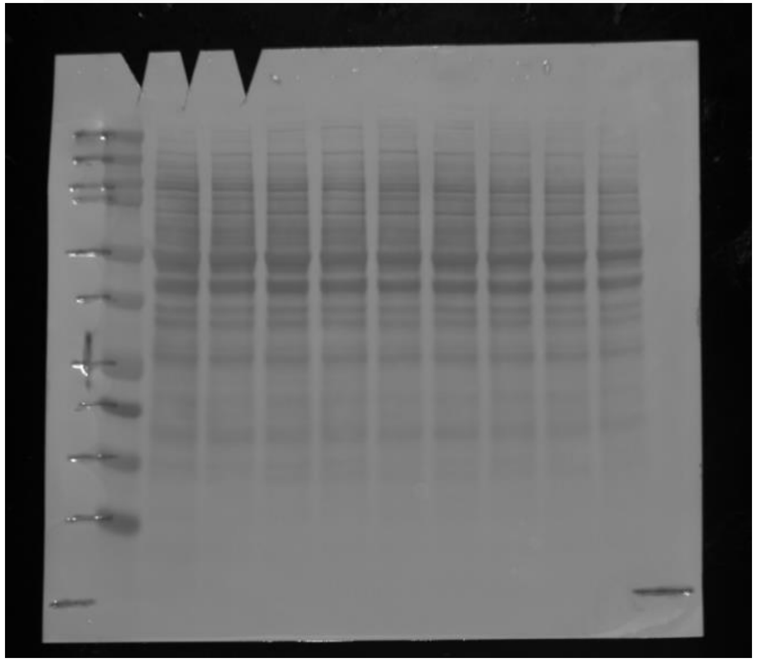

Ponceau

Supplemental Figure 15

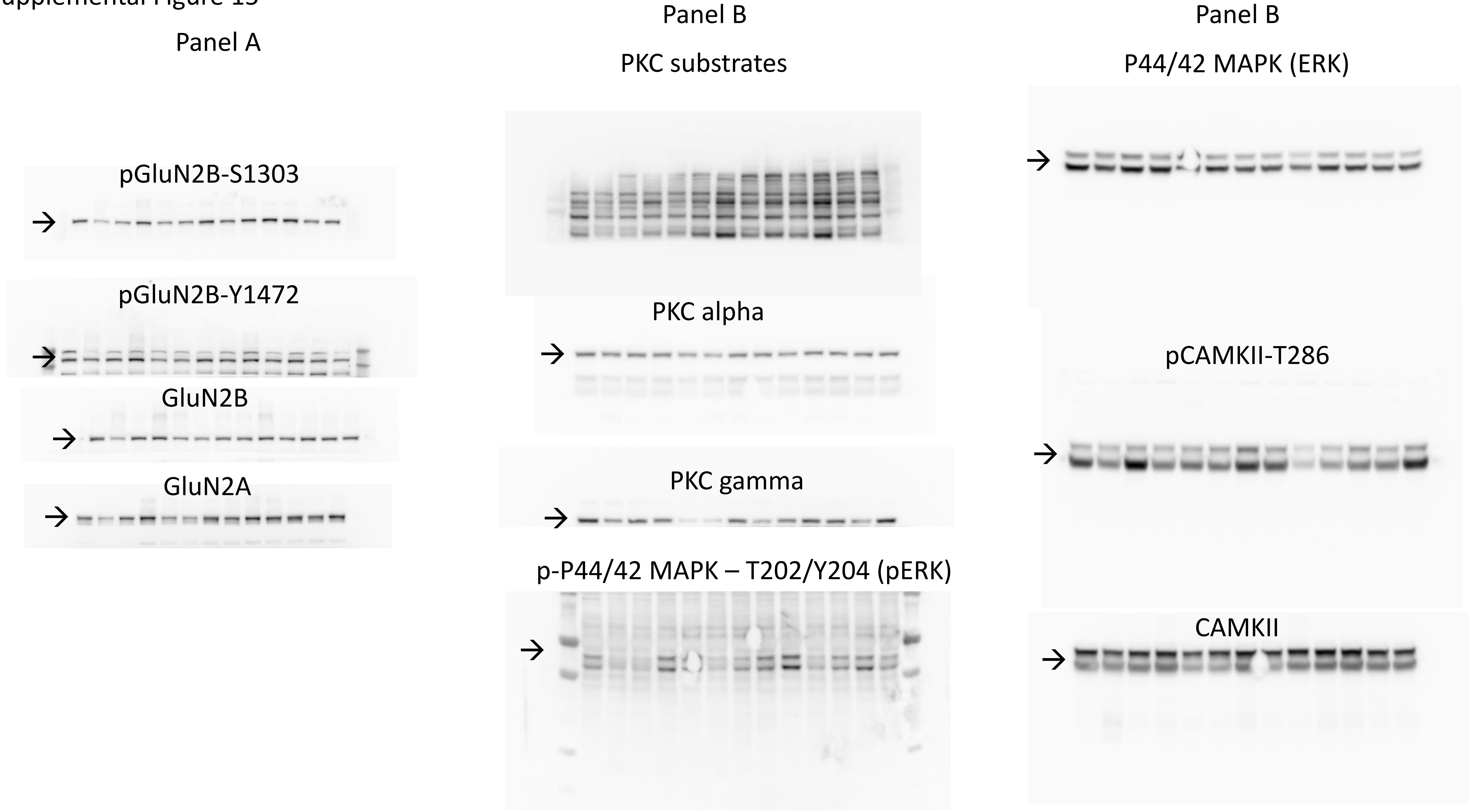

Supplemental Figure 15

Panel C

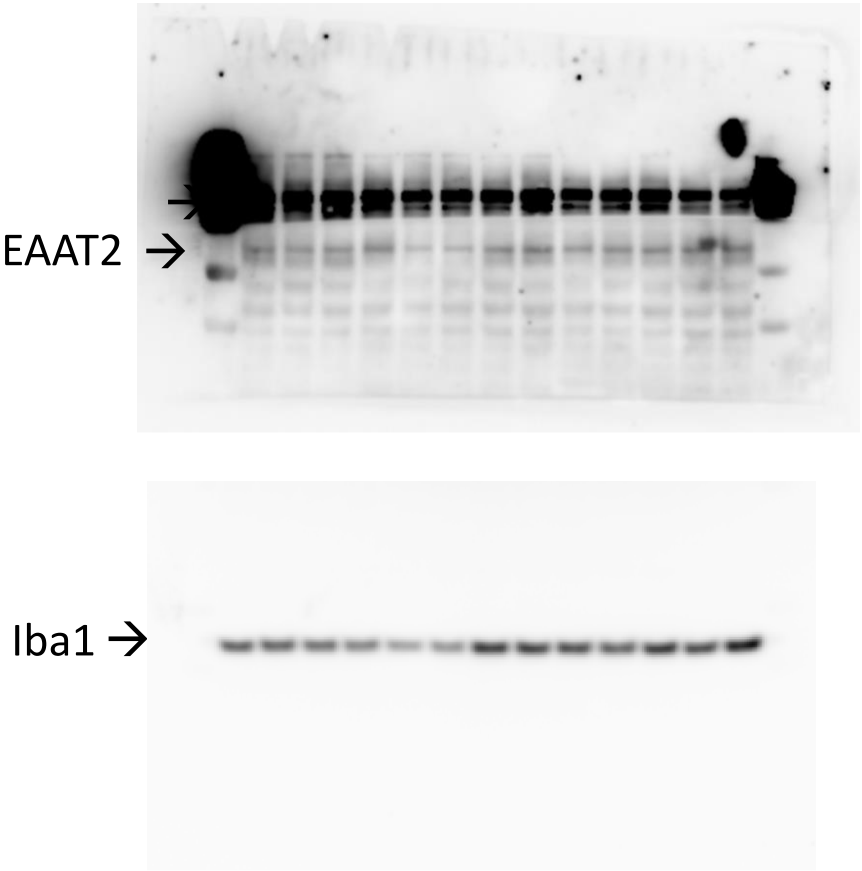

Panel D

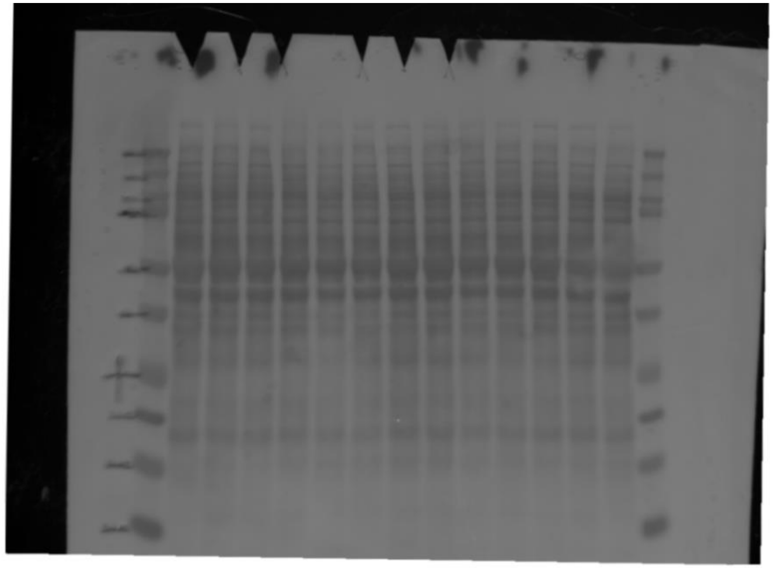

Supplemental Figure 16

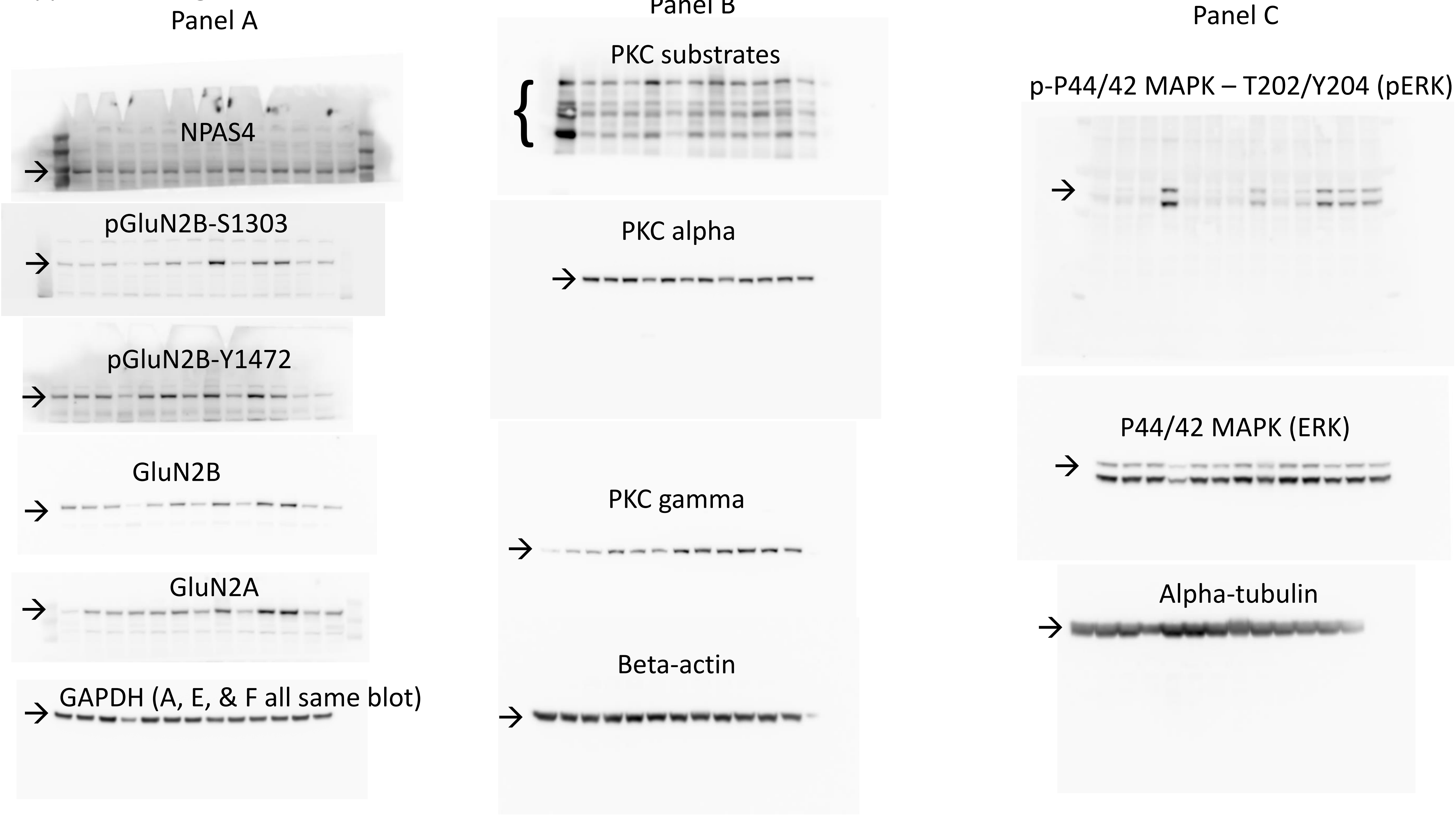

Supplemental Figure 16, con.

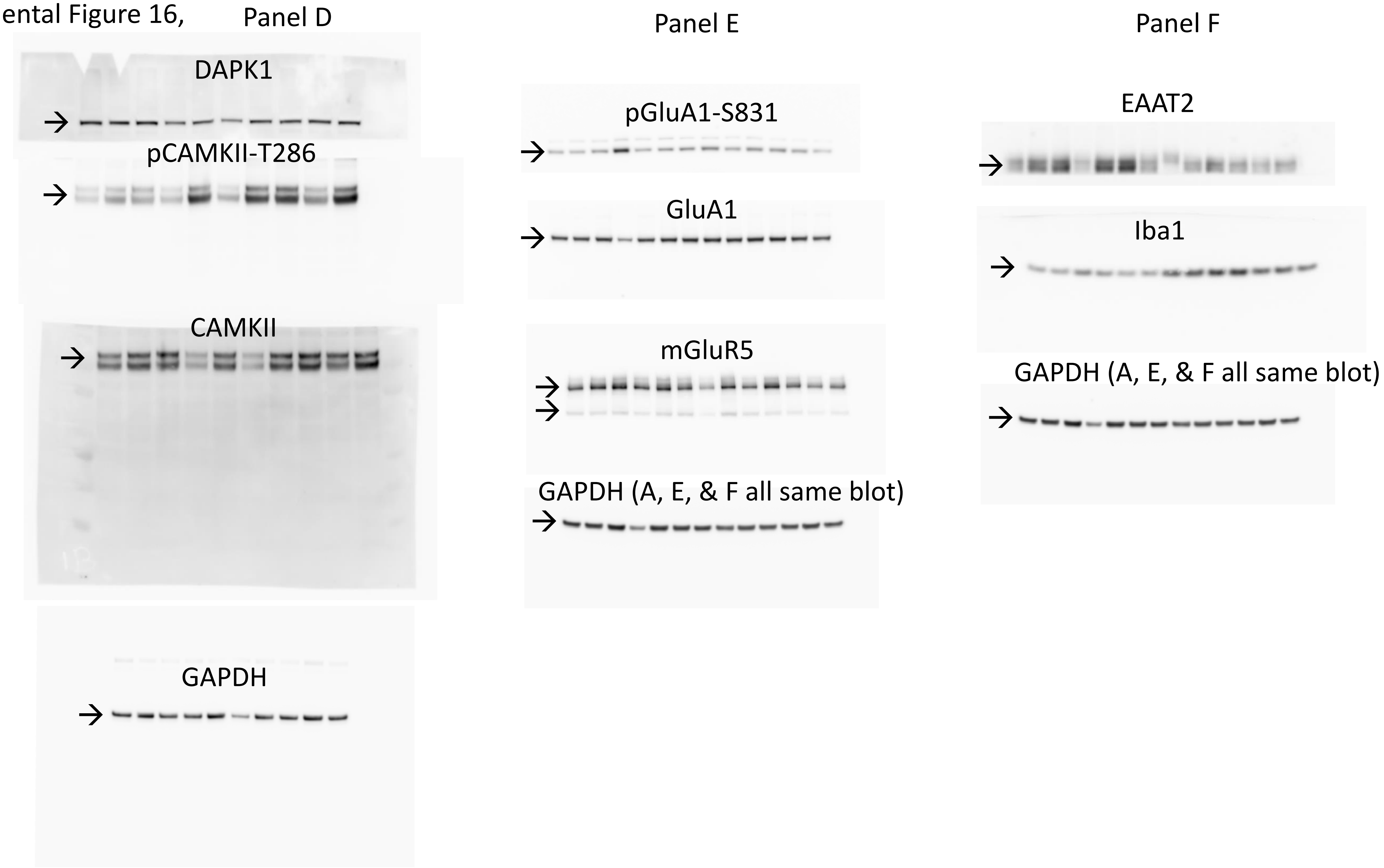

Supplemental Figure 17

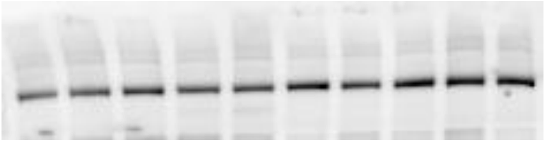

pGluN2B(1303)

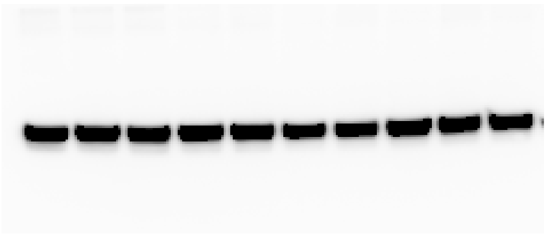

GAPDH FOR pGluN2B(1303)
(not shown in figure)

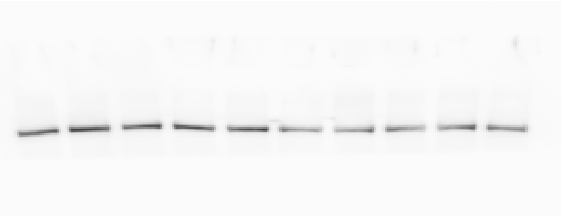

Total GluN2B

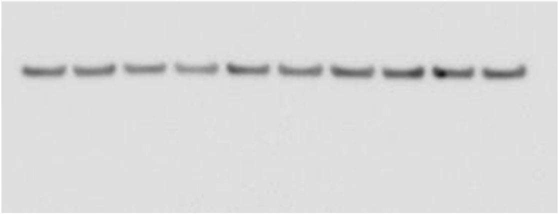

GAPDH for Total GluN2B
(in figure)

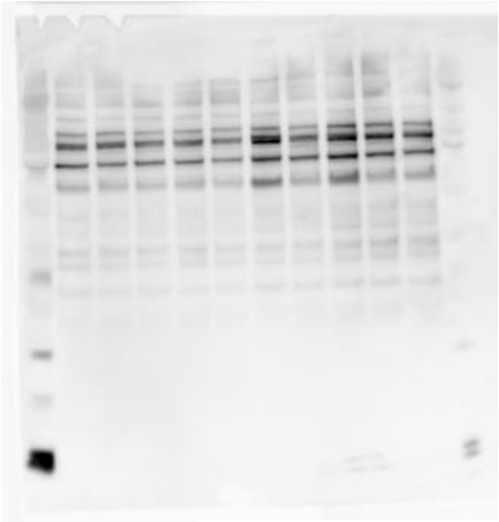

pPKC substrates

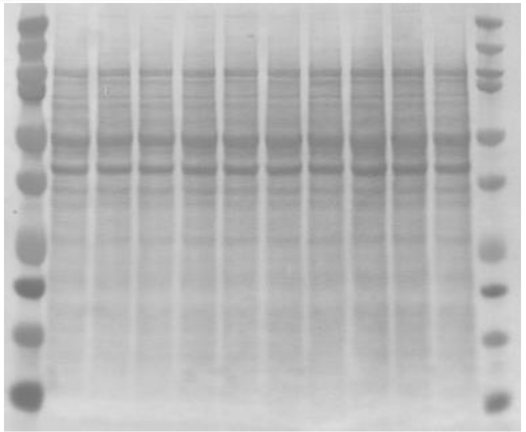

Ponceau
